# DNA as Drug Carrier: A Hydrophobic β‐Cyclodextrin Channel in a Supramolecular Structure

**DOI:** 10.1002/chem.202502895

**Published:** 2026-02-15

**Authors:** Giuseppina Raffaini

**Affiliations:** ^1^ Department of Chemistry, Materials, and Chemical Engineering “Giulio Natta”, Politecnico di Milano Italy

**Keywords:** adsorption, aggregation, cyclodextrins, DNA, drug carrier, supramolecular complexes

## Abstract

The adsorption of drugs and their carriers onto double‐stranded DNA is potentially significant for their transport and release within cells, and β‐cyclodextrins (βCDs) complexed with therapeutic compounds represent promising drug delivery systems. However, the molecular mechanisms governing the formation and behavior of these supramolecular complexes on DNA structures remain poorly understood. Therefore, we employed molecular mechanics and molecular dynamics simulations at an atomistic level to examine the formation of inclusion complexes between βCDs and quercetin (an antioxidant flavonoid with anticancer properties), and their subsequent adsorption and self‐aggregation on DNA structure. The adhesion process results from intermolecular interactions between the hydrophobic drugs included in βCD cavities, which create a uniquely ordered hydrophobic channel that wraps along the DNA grooves. This structure is further stabilized by hydrogen bonds between βCDs, forming a long‐range ordered supramolecular structure over time, akin to a thread enveloping the DNA. This study provides insights into understanding supramolecular complexes involving DNA for potential drug delivery applications.

## Graphical abstract

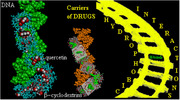

**Abbreviations**
H‐bonds: hydrogen bonds
IC: inclusion complex
MD: molecular dynamics
MM: molecular mechanics
NVT: number of molecules, volume and temperature constant
Q: quercetin
SASA: surface‐accessible surface area
βCD: β‐cyclodextrin

## 1 Introduction

In recent years, significant progress has been made in designing advanced drug delivery systems through nanomedicine development [1, 2, 3]. Nanomedicine formulations aim to optimize the balance between drug efficacy and toxicity [4, 5, 6, 7, 8]. As a relatively new offshoot of nanotechnology, nanomedicine offers promising opportunities in biomedical research for the development new treatment strategies [9, 10, 11, 12, 13]. Many active pharmaceutical ingredients exhibit low oral bioavailability due poor solubility and physical and chemical instability. Materials such as polymers, peptides, biomolecules, antibodies, lipids, and nanocomposites have been used to conjugate with drug molecules, enhancing pharmacokinetic properties and delivery efficiency [14, 15, 16, 17, 18, 19, 20, 21, 22, 23].

In recent years, DNA has garnered considerable attention as a promising biocompatible drug carrier, capable of enhancing bioavailability, and minimizing adverse effects during the drug release process [24, 25, 26]. Typically, DNA intercalation is employed to load chemotherapy drugs, such as doxorubicin, into DNA nanostructures. However, this method does not permit precise control over drug distribution patterns [27]. In a recent communication, Zhao et al. [28] demonstrated that using an amphiphilic system improves cellular uptake efficiency compared to DNA nanostructures, while also providing water solubility for hydrophobic drugs.

The solubilization of hydrophobic drugs with anti‐inflammatory or cancer therapy properties by forming host–guest inclusion complexes with cyclodextrins (CDs) or CD‐based polymers, is of great interest in pharmaceutical applications. These amphiphilic macromolecules are notable for their unique porous structure and the presence of multiple hydroxyl groups [29, 30, 31, 32, 33]. CDs, considered effective drug carriers, are cyclic oligosaccharides composed of *D*‐glucose units linked by α‐1,4‐glucosidic bonds. This structure gives them a 3D torus‐like shape, which is crucial for encapsulating poorly soluble drugs in their hydrophobic cavities [34, 35, 36]. Among the different types of CDs, β‐cyclodextrin (βCD), which consists of seven *D*‐glucose units, is one of the most common CDs [37]. According to the literature, Pawar et al. reported that a β‐cyclodextrin polymer can encapsulate quercetin and doxorubicin in vitro, enhancing their intracellular availability. This delivery method induces apoptosis and morphological changes, thereby confirming their enhanced anticancer effect in resistant cancer cells [38].

Quercetin (Q) is a hydrophobic polyphenolic compound recognized for its potent antioxidant, anti‐inflammatory, and anticancer properties. These attributes make it effective in combating cancer cells, reducing inflammation, protecting against heart disease, and regulating blood sugar levels [39, 40, 41, 42]. Due to its poor solubility and rapid metabolism, developing stable and biocompatible nanocarriers to improve its solubility and intracellular delivery is crucial [43]. Research on the interaction between quercetin and DNA has also produced significant findings [44, 45, 46, 47, 48, 49, 50]. Beyond its antioxidant capabilities, quercetin reduces DNA damage by inhibiting damage responses to induce apoptosis [51]. It promotes cytotoxicity in cancer cells by inducing double‐strand DNA breaks [52] and affects the DNA methylation pattern in tumor therapy [48]. Yoshiba et al. reported an intriguing study on the adsorption dynamics of quercetin with electrospun konjac glucomannan fabric containing double‐stranded DNA [49]. Konjac glucomannan (KGM) is a water‐soluble polysaccharide consisting of randomly linked β‐1,4‐*D*‐glucose and β‐1,4‐D‐mannose of the main chain, with a short‐branched chains connected through β‐1,6‐glycoside linkages [50, 51]. Experiments with KGM‐based electrospun fabric incorporated with DNA showed that the equilibrium adsorbed amount of quercetin increased with increasing DNA content. These experimental results indicate quercetin's strong affinity for DNA structures, with its adsorption–desorption process influenced by DNA content and demonstrating favorable interactions among DNA, quercetin, and polysaccharides.

To enhance the efficacy and bioavailability of quercetin, and regulate its binding to plasma proteins; β‐cyclodextrins (βCDs); βCD‐based nanosponges; and βCD‐based polymers can be employed to form nano‐inclusion complexes (NICs) [38, 52, 53, 54, 55]. Rajamohan et al. reported on the preparation and characterization of a quercetin NIC with β‐cyclodextrin, and assessing its potential in cancer cells applications [54]. These inclusion complexes formed through aggregation enabled by intermolecular hydrogen bonds and *π‐π* interactions among hydrophobic encapsulated drugs, as will be explored in this theoretical work. The enhanced solubility of quercetin NICs with βCD improved cell survival rates in MCF‐7 cells. Zhao et al. emphasized the role of a biocompatible hydrophobic cross‐linked cyclodextrin‐based metal–organic framework as a quercetin nanocarrier, citing enhanced stability and controlled release for nutritional delivery system in the food and biomedical sectors [55]. Pradhan et al. developed and optimized chitosan nanoparticles with quercetin–β‐cyclodextrin inclusion complex (QNPs) using nanoprecipitation [56]. Analyses of these nanoparticles confirmed the successful integration of quercetin within the β‐cyclodextrin complex and a reduction in crystallinity. In vitro drug release studies demonstrated a controlled release profile for QNPs compared to free quercetin and the inclusion complex, suggesting that QNPs may enhance quercetin's therapeutic efficacy, particularly in neurological disorders such as epilepsy. Mondal et al. investigated an anticancer drug (6‐MP) with βCD and its DNA binding properties, discovering enhanced antibacterial activity and photostability without chemical modification [57]. In vitro studies revealed that βCD/6‐MP inclusion complexes, in a 1:1 ratio, were more effective than pure 6‐MP. Furthermore, significant in vitro cytotoxic activity against the human kidney cancer cell line (ACHN) was observed for these inclusion complexes. These findings underscore the significance of βCD inclusion complexes in improving the solubility of hydrophobic drugs and enhancing their cytotoxic and antibacterial properties via favorable DNA interactions. Rocha et al. reported that polymerized βCD exhibits increased binding affinity to DNA, a property that could be utilized to modulate cyclodextrin binding to double‐stranded DNA [57]. In this theoretical work, βCDs adhere to double‐stranded DNA as partially encapsulated quercetin molecules engage in van der Waals interactions along the DNA axis [58]. Alghmandi et al. examined the binding behavior of curcumin (CUR) with DNA alone and in the presence of β‐CD, finding that CUR binds to DNA via intercalation. CUR also binds to β‐cyclodextrin in 1:1 and 1:2 stoichiometry at various concentrations [59]. Further analysis is needed to evaluate in vivo release, as even well‐binding drug complexes could face. Supramolecular complexes with high‐interaction drug carriers could generate a problem from the pharmacokinetic and pharmacodynamic challenges due to slow or incomplete drug release.

Studies on the interaction between DNA and small molecules, such as drugs [60, 61, 62, 63, 64, 65], underscore the significance of intermolecular interactions, varying interaction geometries within DNA grooves, and intercalation processes, with important biological implications [66, 67, 68, 69, 70]. Theoretical investigations employing DFT, molecular docking, and molecular mechanics (MM), and molecular dynamics (MD) methods describe stable interaction geometries involving drugs and DNA [71, 72, 73]. Continually developed and force fields are crucial for understanding biological processes involving DNA, as well as organic and inorganic systems. Parmbsc1, a refined force field for DNA simulations, effectively describes stable and metastable DNA structures and DNA flexibility [74]. In this theoretical study on the adsorption of ICs at various concentrations on a double‐stranded DNA architecture, cyclodextrins, quercetin drug molecules, and fixed DNA are modelled using CVFF, a pivotal force field important in biochemistry, peptide, and protein studies [75, 76, 77]. The initial interaction stage involves the adsorption and self‐aggregation of ICs, facilitated by favorable noncovalent interactions with the external surface of the DNA architecture.

This theoretical study examines the interactions between quercetin molecules and βCDs forming various inclusion complexes, with differing stability levels and stoichiometries. The adsorption and self‐aggregation processes of these βCD/Q inclusion complexes (ICs) on double‐stranded *B*‐DNA are analyzed across different βCD/Q IC concentrations. The investigation aims to elucidate the DNA double helix's role, the impact of hydrophobic quercetin molecules in ordering adsorption along the DNA helix axis, the aggregation processes among pure βCDs and ICs in the same and different concentrations as previously considered, and the adsorption of pure βCDs or quercetin molecules on the DNA surface. The interaction energy between drug and DNA significantly drives the adsorption process of βCD ICs, with IC–DNA interaction energy proving more favorable than that of pure βCDs. Notably, the ICs exhibit more ordered interactions along a preferential direction due to *π‐π* interactions in the presence of quercetin molecules. This theoretical analysis is detailed in the *Results and Discussion* section.

## 2 Materials and Methods

**FIGURE 1:**
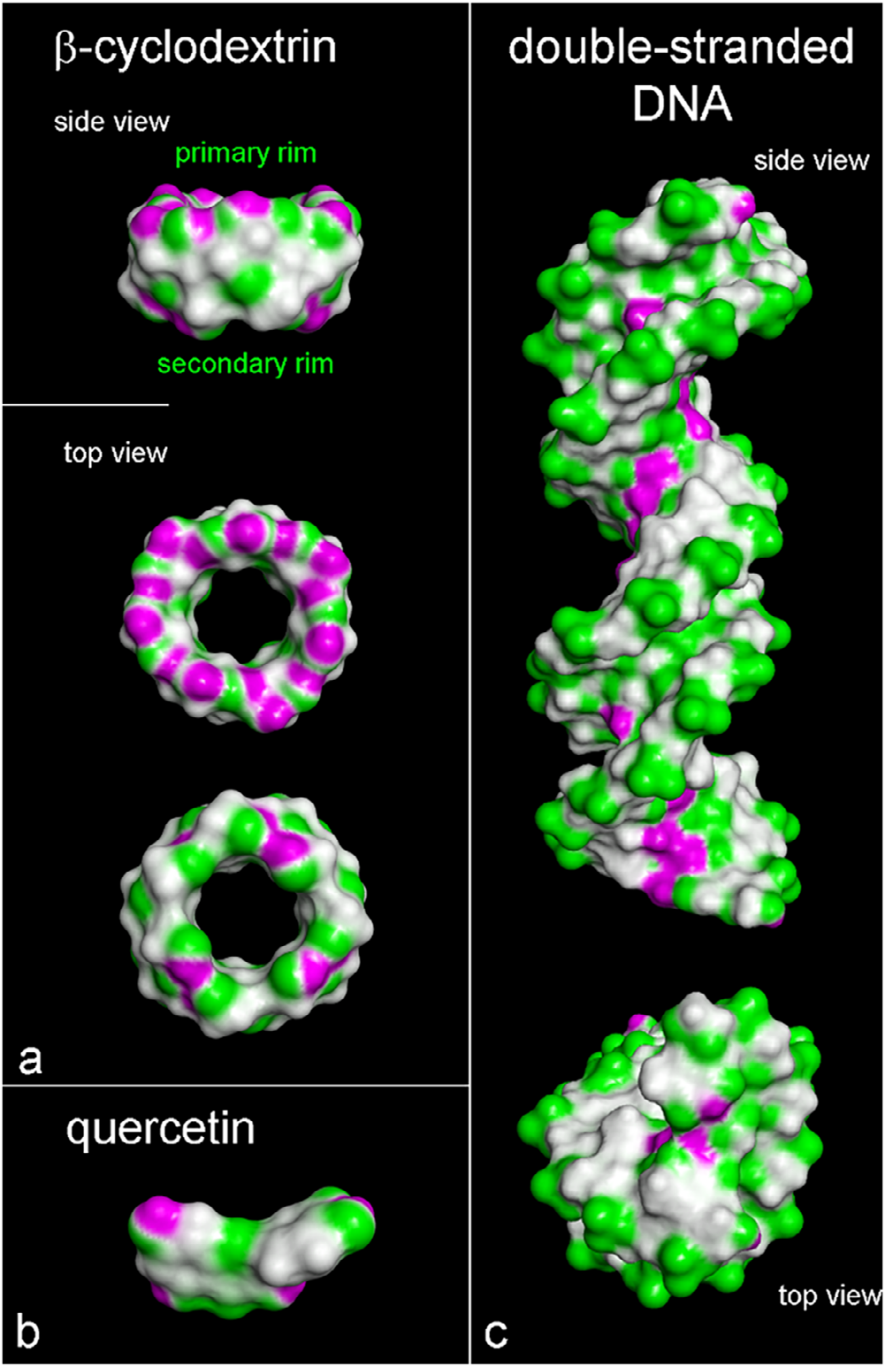
Side view and top view from the secondary and primary rim, respectively, of the optimized geometry of β‐cyclodextrin (Panel **a**). Panel **b** shows the optimized geometry of the quercetin molecule. Panel **c** shows the side view and the top view of the double‐stranded DNA structure. All representations show the surface‐accessible surface area (SASA) colored by hydrogen bond donor–acceptor properties (donor in fuchsia, acceptor in green). The chemical structure of these molecules is shown in Figure S1.

The structure of β‐cyclodextrins (βCDs), the quercetin (Q) molecule, and the double‐stranded *B*‐DNA fragment, as reported in the Protein Data Bank (3CRO) [78], are depicted in Figure S1. Figure 1 illustrates the solvent‐accessible surface areas of βCD, Q, and DNA in Panels **a**, **b**, and **c**, respectively. These surfaces are colored based on hydrogen bond donor–acceptor properties. This depiction not only reveals steric hindrance within the studied systems but also provides insights into potential intermolecular interactions through hydrogen bonds (H‐bonds) formation; a topic discussed in this work. All calculations were performed using the Materials Studio package 7.0 (BIOVIA, [79]) and the CVFF force field, a pivotal force field important in biochemistry, peptide, and protein studies [75, 76, 77]. Consistent with previous studies [80, 81, 82, 83], simulations were conducted in implicit water with a distance‐dependent dielectric constant and periodic boundary conditions. Energy minimizations proceeded until reaching an energy gradient of less than < 4×10−3 kJ mol−1 Å−1. Molecular dynamics (MD) simulations were executed within an NVT ensemble (canonical ensemble, see Abbreviations) at a maintained temperature of 300 K, regulated by a Berendsen thermostat. The integration of dynamical equations used the Verlet algorithm with a time step of 1 fs, and instantaneous coordinates were saved periodically for subsequent analysis. During the MD runs, we calculated the time evolution of potential energy, van der Waals, and Coulomb contributions. Additionally, we monitored the relative concentration profile (see Supporting Information), observing the initial energy decrease due to favorable noncovalent interactions during the adsorption process, followed by fluctuations around an average value during the self‐aggregation process on double‐stranded DNA.

When investigating noncovalent interactions with DNA, the DNA fragment is positioned centrally in the simulation cell and remains fixed during molecular mechanics or dynamics (MM/MD) simulations. This approach allows for a focused study on the initial intermolecular interactions between DNA and cyclodextrin inclusion complexes (ICs) with quercetin at varying IC concentrations. Further MD simulations were conducted to statistically enhance the description of this initial adsorption and self‐aggregation process on the double‐stranded DNA structure involving ICs at different concentrations, specifically to βCD/Quercetin in an 8:8 and 12:12 stoichiometries, as reported at the end of the specific paragraphs in *Results and Discussion* section.

To further assess potential conformational changes in double‐stranded DNA and the formation and stability of hydrated ICs in an explicit solvent, simulations must also include an aqueous environment without a fixed DNA structure. Such investigations are critical due to the role of hydrophobic interactions in the presence of water molecules [84, 85, 86]. Previous theoretical work by our research group, utilizing an implicit solvent (characterized by the distance‐dependent dielectric constant of water), examined the formation of various inclusion complexes involving cyclodextrins, such as β‐cyclodextrin, γ‐cyclodextrin, or β‐cyclodextrin‐based nanosponges. These findings were compared with subsequent studies in explicit water and were aligned with NMR data reported in the literature [87, 88, 89, 90, 91, 92, 93, 94]. Additional MD simulations were conducted to provide a more comprehensive statistical description of the initial adsorption and self‐aggregation processes involving ICs at the different concentrations detailed in this work.

### 2.1 Intermolecular Interactions Between βCDs and Quercetin Drug Molecules

The simulation protocol consists of three steps [80]. First, the energy minimization of eight initial trial geometries for quercetin drug molecules near the primary and the secondary rim of βCDs is conducted within a cubic simulation cell, as illustrated in Figure S2. Next, eight NVT MD runs are conducted at a constant temperature of 300K, each lasting 5 ns, to achieve equilibrium and study the kinetics of the hydrophobic drug's inclusion into the β‐CD hydrophobic cavity. Lastly, numerous configurations saved during the MD run are optimized, alongside the final geometry reached at the end of these simulations. All MD runs for the analyzing the host–guest ICs employed a distance‐dependent water dielectric constant. Four distinct ICs, differing in stability and/or geometry, were identified and discussed. Further calculations, including those in water and the free energy calculations, will be conducted to expand upon the findings presented in this study. The results are elaborated in the main text.

### 2.2 Intermolecular Interactions Between βCD/Quercetin Inclusion Complexes and Double‐Stranded *B*‐DNA

In line with previous research [81], the periodic boundary conditions and initial random arrangements of different host–guest complexes in a simulation cell containing the double‐stranded *B*‐DNA are considered. All MD runs were carried out in the NVT ensemble, maintaining a constant number of particles, volume, and temperature. The simulations utilized a cubic cell with edges equal to 148.5 Å, positioning the DNA structure centrally within the simulation box. The DNA structure was fixed during energy minimization and throughout all MD runs. The structure of double‐stranded *B*‐DNA fragments is documented in the Protein Data Bank under the accession number3CRO [78]. Four distinct stable and metastable βCD/quercetin ICs, depicted in Figure S4, were randomly inserted into the simulation cell as illustrated in Figure S15.

The simulation protocol involved three main steps: (I) energy minimizations of the initial geometries; (II) NVT MD runs at constant lasting for 50 ns, with an analysis of the numerous conformations assumed by the systems periodically saved during the MD run every 20 ps; (III) geometry optimizations and analysis of the systems at the end of the MD runs when an equilibrium state was achieved, and of different significant conformations assumed by the system during the MD simulations.

During the simulation, the adsorption process onto the DNA and the self‐aggregation of βCDs and quercetin on the DNA structure architecture occurred. Particularly, at larger concentrations of βCDs and quercetin, wrapping behavior along the DNA axis was observed. This same simulation protocol was applied to study interactions between DNA and ICs at increasing concentrations, specifically eight, twelve and sixteen host–guest complexes initially randomly distributed in a simulation box. The findings from this investigation are elaborated upon in the main text.

### 2.3 Intermolecular Interactions Between Quercetin Molecules and Double Stranded *B*‐DNA

Initially, as in a previous study [81], the intermolecular interactions between DNA at the center of a cubic simulation cell with edges equal to 148.5 Å, and a single quercetin molecule was examined. The quercetin molecule started near either a minor or major groove in a parallel or perpendicular orientation relative to the DNA. The adsorption process occurred rapidly during an MD run lasting for 10 ns, with the DNA remaining fixed in the simulation box. The results of this investigation are discussed in the main text.

The adsorption process of quercetin molecules at the same concentration previously studied without βCDs was further investigated using a cubic simulation cell, this time including only the DNA structure in the central part. Adopting the same methodology, we considered four, eight, twelve, and sixteen quercetin molecules positioned near the *B*‐DNA structure. After energy minimization, NVT MD simulations were conducted at a constant temperature equal to 300K for 10 ns. Equilibrium was assumed upon achieving optimized final geometries. The results of this investigation are elaborated upon in the main text.

### 2.4 Intermolecular Interactions Between B‐DNA and βCDs at Different Concentrations

Initially, as outlined in the simulation protocol adopted in previous work [81], we examined the intermolecular interactions between the DNA, located in the center of the cubic simulation cell with edges measuring 148.5 Å, and a single βCD molecule positioned near either a minor or major groove. This interaction was assessed from six different initial configurations: the βCD primary rim parallel to a DNA minor or major groove, the βCD secondary rim parallel to a DNA minor or major groove, and the βCD best‐fit plane perpendicular to the DNA axis near a minor or major groove. The adsorption process proceeded rapidly during an MD run lasting for 10 ns, with the DNA held fixed in the simulation box. The findings of this investigation are detailed in the main text.

The adsorption process of βCD molecules was investigated at the same concentration previously studied without quercetin molecule, using a cubic simulation cell with the DNA structure positioned centrally. The methodology was consistent with earlier experiments. The study considered scenarios involving four, eight, twelve, and sixteen quercetin molecules in proximity to the *B*‐DNA structure. Following energy minimization of the system, NVT MD simulations were conducted at a constant temperature of 300K for 20 ns. The final geometries were optimized once equilibrium was achieved. The findings from this investigation are discussed in the main text.

### 2.5 Intermolecular Interactions Between βCDs at Different Concentrations

To investigate the intermolecular interactions between βCDs within a simulation cubic cell (148.5 Å per edge) at different concentrations, we utilized the same concentrations as previously studied in systems containing double‐stranded DNA, although this time without DNA or drug molecules. The βCDs were initialized arranged randomly, as depicted in Figure S31. The simulation protocol included the following steps: *i*) energy minimization of the system containing four, or eight, or twelve or sixteen βCDs arranged randomly in the simulation cell; *ii*) MD simulations conducted a constant temperature of 300K for a duration of 20 ns; *iii*) energy minimization of the system following the MD runs, including periodic energy minimization of selected frames saved during the MD runs. The findings from this investigation are detailed in the main text.

### 2.6 Intermolecular Interactions Between βCDs and Quercetin Molecules at Different Concentrations

The intermolecular interactions between βCDs and quercetin molecules in a cubic cell (with edges measuring 148.5 Å) at various concentrations were investigated. These concentrations mirror those previously examined in the presence of double‐stranded DNA. In this scenario, however, the double‐stranded DNA was absent, and no preformed ICs existed. Following the strategy adopted in prior study [82, 83] βCDs and quercetin molecules were randomly initialized within the simulation cell, as depicted in Figure S34. The same simulation protocol used for assessing the intermolecular interactions between βCDs at different concentrations was applied, involving energy minimization, MD runs lasting 20 ns, and optimization of system's various conformations. The findings from this investigation are discussed in the main text.

## 3 Results and Discussion

The intermolecular interactions between β‐cyclodextrins (βCDs) and quercetin molecules in 1:1 and 2:1 stoichiometries, as well as within stable 1:1 ICs at a higher concentration, were investigated concerning their adsorption onto double‐stranded DNA [80]. Additionally, the interactions between DNA and quercetin, DNA and βCDs [81], and among βCDs at concentration previously studied, were examined separately [82, 83]. Each of these simulated systems represents a component of a broader analysis, which will be discussed in the Conclusion.

### 3.1 Intermolecular Interactions Between βCDs and Quercetin Drug Molecules in 1:1 and 2:1 Stoichiometries

The study investigated the potential ICs formed between β‐cyclodextrin (βCD) and the quercetin drug molecule in a 1:1 stoichiometry. Subsequently, the research examined a dimer formation involving a stable IC previously obtained through MD simulations. This included the addition of another βCD to explore the formation of stable cyclodextrin dimers with βCDs oriented either two primary rims, by one primary and one secondary rim, or by two secondary rims were investigated [80]. These more intricate host–guest complexes may yield insights into the various possible interactions with two cyclodextrins. They also offer information on the mobility of the drug within an extended hydrophobic internal volume, as discussed in the following paragraphs.

### 3.2 Inclusion Complexes of βCD/Quercetin in a 1:1 Stoichiometry

Initially, the potential ICs between βCD and quercetin in a 1:1 stoichiometry were investigated [80]. The simulation protocol designed to study stable and metastable ICs involves three steps:

*i*) Six different initial geometries of βCD and quercetin (Figure S2) were studied, where the quercetin molecule was positioned close to the outer surface of the cyclodextrin, either parallel or perpendicular to its primary or secondary rim without prior drug inclusion.
*ii*) Following initial energy minimizations (Figure S3 and Table S2), four optimized geometries were identified. These geometries, with the B aromatic ring positioned near βCD's primary or secondary rim, displayed different inclusion and stability characteristics. These configurations served as the starting point of four MD runs lasting 5 ns.
*iii*) Energy minimization was conducted on the conformation assumed at the conclusion of four MD runs, once equilibrium was achieved (Figure S4 and Table S3), as well as on select frames periodically saved during the MD run [38].

The time evolution of potential energy, van der Waals contribution, and Coulomb energy during the 5 ns MD runs for the 1000 frames saved every 1 ps are presented in Figures S5, S6, S7, and S8, respectively. Additionally, the distances between the centers of mass (c.o.m.) of βCD and quercetin over time and the distribution of these distances calculated during each MD run are provided.

According to Table S2, which details the data for the initial optimized geometries shown in Figure S3, the interaction energy and both intramolecular and intermolecular hydrogen bonds (H‐bonds) calculated for the four host–guest complexes obtained after four MD runs are compiled in Table S3. Notably, using this simulation protocol, starting from six distinct initial geometries without preestablished drug inclusion, both stable and metastable complexes formed over time. These complexes exhibited similar potential and interaction energies between βCD and quercetin.

In particular, three geometries with comparable stability featured the B aromatic rim near the βCD's primary or secondary rim. The longest axis of the ellipsoid containing the quercetin molecule was approximately perpendicular to the plane defined by the glycosidic oxygens of the macrocycle, thus minimizing the steric hindrance of the included drug (geometries P1, P2, and S2 in Panels **a1**, **b1,** and **b2** of Figure S4. Another metastable geometry found after the MD run, showed the quercetin included with the B aromatic rim near βCD's usually larger secondary rim, with the ellipsoid's longest axis roughly parallel to the glycosidic oxygen plane (geometry S1 in Figure S4). The flexibility of βCD's cyclic structure, forming intermolecular interactions like hydrophobic interaction within its hydrophobic cavity and H‐bonds involving hydroxyl groups, effectively minimized the steric hindrance of the included drug.

From the energetic perspective, starting from the four geometries depicted in Figures S3, the MD simulations reveal that the βCD/quercetin host–guest complexes reach an equilibrium state characterized by energy fluctuations around an average value (Figures S5, S6, S7 and S8). Geometrically, quercetin is primarily situated within the hydrophobic cavity of βCD for most of the simulation duration. Some larger distances between the centers of mass of βCD and quercetin are calculated have been observed. The literature indicates that cyclodextrins are flexible and can encapsulate drugs; however, this enthalpy‐driven process is primarily due to a weak van der Waals interactions. Consequently, at 300K, the drug may temporarily distance itself from the cyclodextrin's center of mass but can still be incorporated over time. The formation of ICs at 300K is thus a dynamic process.

### 3.3 Inclusion Complexes of βCD/Quercetin in a 2:1 Stoichiometry

Following the study of stable or metastable ICs between βCD and quercetin in a 1:1 stoichiometry, the more stable host–guest complex, denoted as geometry P1 in Figure S4, was further analyzed. A second βCD was subsequently introduced approached, considering three distinct interaction geometries: two primary rims facing each other, one primary and one secondary rim facing each other, and both secondary rims facing each other (Panel **a** in Figures S9, S11 and S13). The initial optimized geometries and both the side and top view of the final optimized geometries of the three different βCD/quercetin dimers in a 2:1 stoichiometry, following a 5 ns MD run, are depicted in Panels **b**, **c1**, and **c2** of Figures S9, S11 and S13, respectively.

Figures S10, S12, S14 present the potential energy, van der Waals contributions, and the Coulomb energy calculated during the MD run in Panels **a** and **b**. The distances between the two βCDs and their time distribution are illustrated in Panels **c** and **d** of these figures. Notably, the dimers formed with two primary rims or one primary rim and one secondary rim have more frequently observed distances of approximately 8.4 and 7.80 Å, respectively. Particularly in the dimer with two secondary rims facing each other, the most common distance is shorter at 7.21 Å, indicating a more compact structure.

Panels **e** and **f** of Figures S10, S12 and S14 display the distances calculated during MD runs between the two βCD centers of mass and the centers of mass of the quercetin drug within the three different dimers. The calculations revealed an anticorrelation regarding the drug's position, as it alternates between proximity to one cavity and the other, allowing interaction over time with both cavities. In the first dimer configuration, involving a primary and the secondary rim, quercetin is nearly equally distributed between the two βCDs with distance distributions centered around 4.1 and 4.3 Å. In the second dimer, which involves one stable complex in a 1:1 stoichiometry and another βCD's secondary rim, the drug's occupancy shifts toward improved interaction with the second CD over time. Consequently, the distributions of the distances are centered at 5.0 Å initially and shift to approximately 2.62 Å for the second CD. Finally, in the third dimer, quercetin transitions from the first βCD cavity to the second, displaying distributions of 4.71 and about 2.4 Å, respectively.

Initially, the mobility of this hydrophobic drug is sterically hindered due to its inclusion in the hydrophobic cavity of βCD. Over time, as two βCDs interact and form a stable dimer, the drug can move freely between them. This freedom of motion arises from the creation of a new hydrophobic region formed by the merging of the two hydrophobic βCD cavities.

### 3.4 Intermolecular Interactions Between *B*‐DNA and βCD/Quercetin Host–Guest Complexes

The study examined the intermolecular interactions between double‐stranded DNA and various 1:1 βCD/Q ICs, which had been obtained from MD simulations and demonstrated varying degrees of stability (Figure S4). Initially, these complexes were randomly arrangement within a simulation cell at different concentrations [81]. The investigation began with four specific 1:1 βCD/Q ICs, designated as geometries P1, P2, S1, and S2 (Table S3 and Figure S4). Subsequently, the study expanded to include eight, twelve, and finally sixteen host–guest ICs.

The adsorption process on the DNA surface and the self‐aggregation involving host–guest inclusion complexes occurred as follows: β‐cyclodextrins (βCDs), with quercetin drug molecules encapsulated in their hydrophobic cavities adsorb onto the external double‐stranded DNA structure. The stabilization of adsorption was facilitated by favorable hydrogen bonds between βCDs. Initially, hydrophobic interactions between the encapsulated drugs were beneficial, but the drugs’ mobility within the βCD cavities allowed for increasing connectivity over time, which could lead to a more ordered adsorption on the DNA structure. Notably, at higher concentrations, 1:1 βCD/Q inclusion complexes formed a more discernible hydrophobic channel aligned with the DNA.

After the MD simulation, the host–guest complexes arranged themselves along the DNA surface into an ordered long‐range hydrophobic channel, which was more pronounced at higher concentrations. Hydrogen bonds provided stability over time to this supramolecular structure around the DNA grooves, functioning much like a thread. The mobility of the quercetin drug molecules allowed for partial exposure from the βCD rim, leading to their partial release onto DNA grooves. This is likely the initial step of intercalation, which could be explored in future research.

**FIGURE 2:**
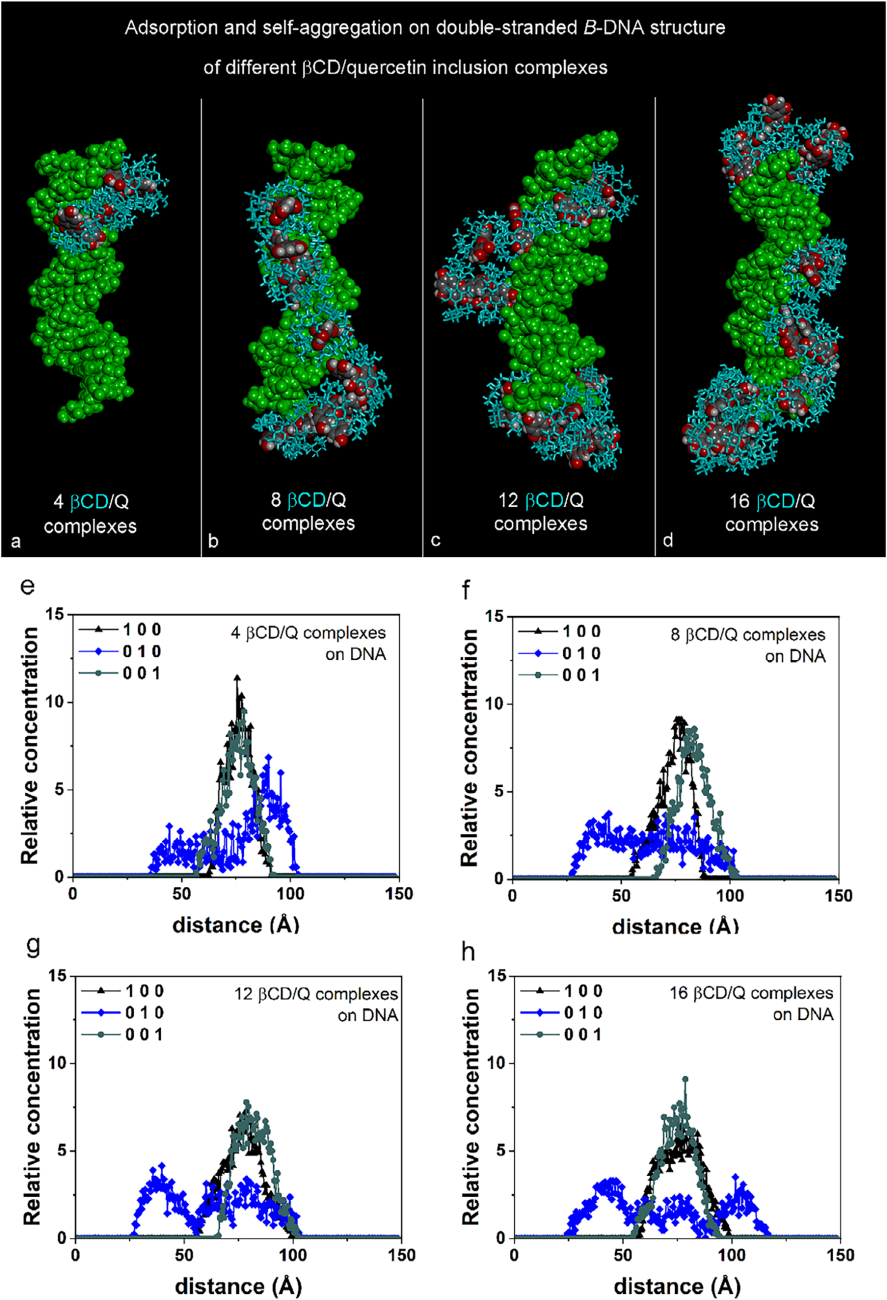
Side view of the optimized geometries obtained after MD runs lasting 50 ns related to the adsorption and self‐aggregation processes of four different kinds of βCD/Q inclusion complexes (P1, P2, S1, and S2 geometries in Table S3); in particular, four, eight, twelve, and sixteen βCD/Q host–guest complexes in Panels **2a**, **2b**, **2c**, and **2d**, respectively. The DNA is colored in green and the quercetin molecules (carbon atoms in gray, oxygen atoms in red, hydrogen atoms in white) are in CPK representation. All βCD carriers are in light blue sticks. Panels **2e**, **2f**, **2g**, **2h**: Relative concentration as a function of the distance calculated for the optimized geometries obtained at the end of MD runs lasting for 50 ns starting from four, eight, twelve, and sixteen βCD/Q inclusion complexes initially in a random arrangement in the simulation cell containing the DNA in the central part (see Figures S15, S18, S22 and S26).

The concentration of drugs and their carriers influences the adsorption and self‐aggregation processes. The following sections discuss these processes concerning DNA, involving four, eight, twelve, or sixteen 1:1 βCD/Q ICs, each originating from four initial types of inclusion complexes previously identified. Figure 2 presents the final optimized geometries that will be discussed in the following (Panels **a**, **b**, **c**, **d**). Additionally, Figure 2 includes the relative concentration as a function of the distance, calculated from the optimized geometries obtained at the conclusion of MD runs (Panels **e**, **f**, **g**, **h**).

### 3.5 Intermolecular Interactions Between *B*‐DNA and βCD/Quercetin Host–Guest Complexes in a 4:4 Stoichiometry

The intermolecular interactions between double‐stranded DNA and four distinct 1:1 βCD/Q inclusion complexes—each exhibiting varying stability and differing drug inclusion geometries (Figure S4)—were investigated using the simulation protocol outlined in *Materials and Methods* section [81].

Figure S15 illustrates the initial, nonoptimized geometry under investigation. During a 50 ns MD run, preferential adsorption on DNA and the self‐aggregation process involving four distinct 1:1 βCD/Q ICs occurred within the major groove of the DNA. Figure S16 presents the final, optimized geometry obtained when the MD run reached equilibrium. Figure S17 details the potential energy, including van der Waals contribution (S17a) and Coulomb contributions (S17b), calculated during the 50 ns MD run. The lack of significant conformational changes from 20 to 50 ns indicates favorable adsorption with localized changes over time. The concentration profiles for all atoms in the simulation cell, shown from 0 to 20 ns and from 20 to 50 ns in Figures S17c and S17d, were nearly indistinguishable, reflecting an equilibrium state. Only minimal local motion occurred in the resulting stable structure. Figure S16b provides a detailed view of interactions between two ICs: two βCDs interacting via their primary rim and the secondary rims and a *π*‐*π* interaction with B aromatic rings of two quercetin molecules partially exposed to each other. Notably, connecting the centers of mass of the three adsorbed cyclodextrins that substantially face each other by their secondary edges forms a nearly equilateral triangle with side lengths averaging (10.7 ± 12.5) Å.

**FIGURE 3:**
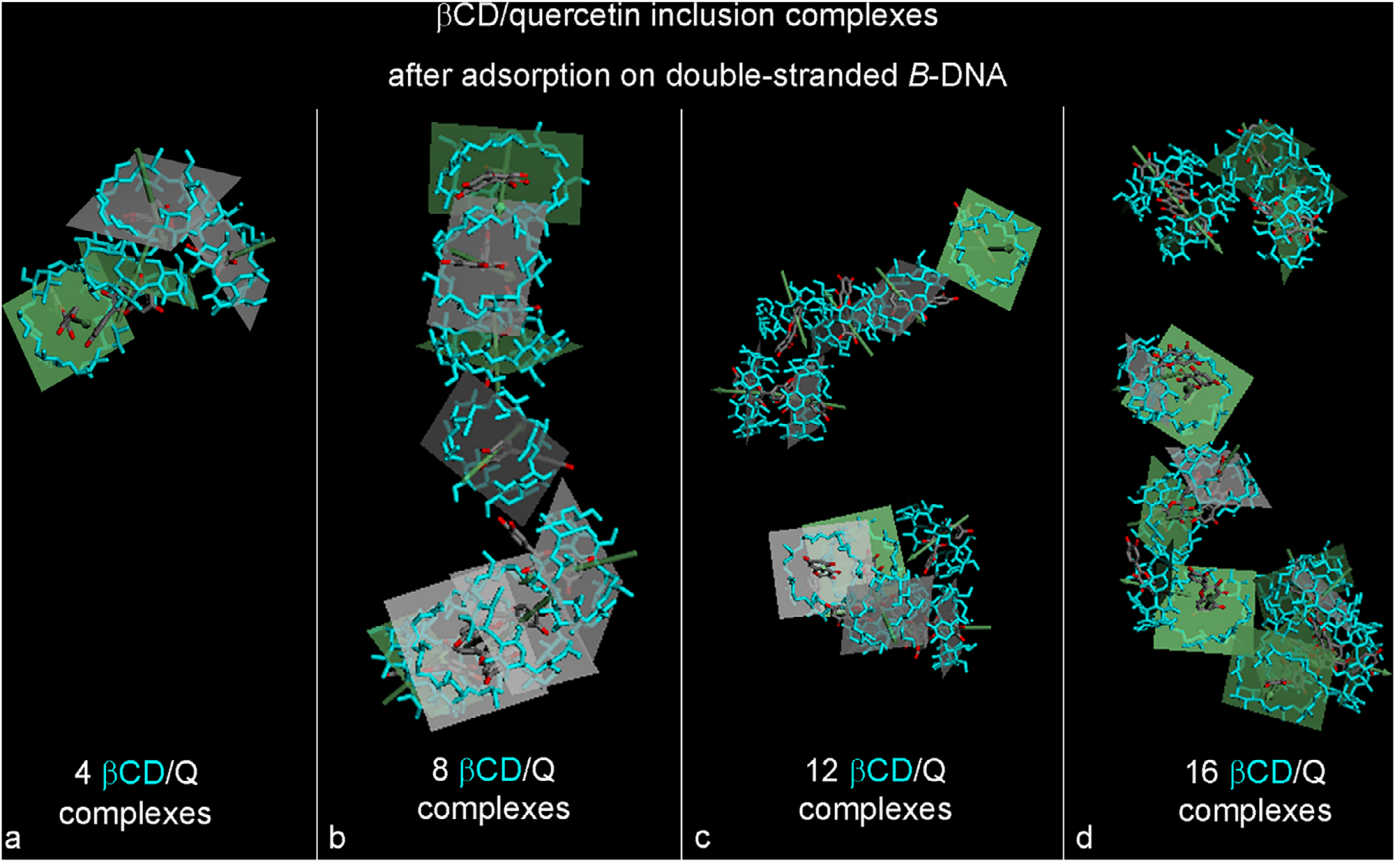
Side view of only the βCD/Q inclusion complexes adsorbed on double‐stranded DNA after an MD run lasting 50 ns as shown in Figure 2, considering four, eight, twelve, and sixteen βCD/Q ICs adsorbed on DNA architecture not shown for clarity in Panels **a**, **b**, **c**, **d** respectively. The DNA structure and the hydrogen atoms are omitted for clarity. The best‐fit planes calculated for all βCD in light blue sticks are represented with an arrow direct to the secondary rim of all βCDs.

This arrangement suggests interesting ordering and adsorption patterns of self‐aggregating complexes on the DNA surface, as depicted in Figure 2a. Figure 2 also displays the concentration profile for all atoms calculated in the final optimized geometry. For all βCDs, Figure 3a shows the best‐fit plane that passing through the seven glycosidic oxygens of each β‐cyclodextrin macrocycle. The plane appears in lighter green when facing the secondary edge of the βCD, with an arrow indicating the rim's direction. Envisioning a continuous channel, this channel, formed by the hydrophobic cavities of the cyclodextrins linked to the hydrophobic drug quercetin, is ideally enclosed in an ellipsoid placed centrally within the CD, with the major axis aligned with the cyclodextrin edges. This hydrophobic channel resembles the continuous layers of azahelicenes on DNA's external surface, which can favorably interact within its grooves [81].

### 3.6 Intermolecular Interactions Between *B*‐DNA and βCD/Quercetin Host–Guest Complexes in an 8:8 Stoichiometry

Figure S18 presents the initial nonoptimized geometry of eight host–guest complexes, two for each of the four ICs previously investigated and depicted in Figure S4 [81]. During an MD simulation lasting 50 ns, as previously observed, these complexes adsorb onto DNA and undergo self‐aggregation. This process occurs along the DNA structure, enveloping the complexes in both the minor and major grooves, as well as at the ends of the DNA.

Figure S19 illustrates all ICs inclusion complexes in their final, optimized geometries at the conclusion of MD run. In this state, equilibrium contributes to forming a channel along the DNA surface. Figure S20 provides information on potential energy, van der Waals contribution (**S20a**), and Coulomb energy (**S20b**), calculated during the 50 ns MD run. As previously noted in the case of the four βCD/Q ICs, no significant conformational changes occurred between 20 and 50 ns, indicating favorable adsorption with some local variations throughout the MD run.

The concentration profiles of all atoms calculated within the simulation cell, displayed in Figures S20c and S20d, are practically superimposable for the periods from 0 to 20 ns and from 20 to 50 ns. This similarity suggests that an equilibrium state was achieved, with only minimal motion due to the specific mobility of quercetin molecules within the stable structure formed. Figures S20b1 and S20b2 detail the arrangement of quercetin around the DNA surface in the final optimized geometry. Notably, the π‐π interaction between drug molecules aligns along an imaginary continuous curved line. Figure S20c further details of the arrangement of βCDs and drugs, highlighting the best‐fit plane for each with an arrow indicating the direction of each secondary edge. Calculating the distance between the centers of mass of the cyclodextrins along the imaginary curved line reveals an average distance of (10.9 ± 2.45) Å.

Figure 2b illustrates the final, optimized geometry, while the concentration profiles of all atoms within the simulation cell were calculated based on this geometry. Figure 3b presents the best‐fit plane for all βCDs, passing through the seven glycosidic oxygens of the macrocycles of each β‐cyclodextrin. One can envision a continuous hydrophobic channel formed by the interacting βCD hydrophobic cavities, in conjunction with the volume occupied by the hydrophobic drug molecules, which are partially included in and partly exposed by the βCD rims. This configuration creates an overall channel where hydrophobic interactions, exerted at varying distances, cooperatively engage with the DNA structure through hydrogen bonds formed by numerous host–guest complexes adsorbed onto it. Notably, the winding direction of these host–guest complexes on the DNA surface contrasts with that of double‐stranded *B*‐DNA.

For a more robust statistical analysis of the adsorption and aggregation of the βCD/Quercetin inclusion complexes discussed above onto double‐stranded DNA, three further MD simulations were carried out under the same conditions (cell dimensions, distance‐dependent dielectric constant of water, NVT ensemble) but with different initial random arrangements of eight ICs around the DNA structure. The DNA was fixed in the center of the simulation box throughout. The three new initial nonoptimized geometries are illustrated in Panels **a1**, **b1**, and **c1** of Figure S21. After initial energy minimization, 50 ns MD runs, and geometry optimization of the systems at equilibrium, the interaction energies between the DNA and eight βCD/Quercetin host–guest complexes were calculated. Considering all four optimized geometries obtained from the four MD runs (the first discussed above and the three additional simulations), the average interaction energy is equal to (−3095 ± 82.38) kJ/mol. In the final optimized geometries, βCD/Quercetin inclusion complexes with 1:1 stoichiometry are more prevalent than those with 2:2 stoichiometry; nevertheless, the interacting drug molecules remain spatially close around the DNA. A common feature of all final optimized geometries is a continuous space occupied by hydrophobic cavities and hydrophobic drug molecules aligned along the DNA structure, forming a new supramolecular complex, as illustrated in Panels **a2**, **b2**, and **c2** of Figure S21.

Of particular interest is the arrangement of the quercetin drug molecules as illustrated in Panel **a3** of Figure S21: the average distance of the center of mass of all eight quercetin molecules in β‐cyclodextrin inclusion complexes around the double‐stranded DNA is equal to 5.698 ± 2.511 Å, confirming the ordering around the DNA structure of hydrophobic drugs in hydrophobic cavities of β‐cyclodextrins in ICs initially randomly distant from the DNA structure.

### 3.7 Intermolecular Interactions Between *B*‐DNA and βCD/Quercetin Host–Guest Complexes in 12:12 Stoichiometry

Figure S22 presents the initial non‐optimized geometry of twelve 1:1 βCD/Q ICs, previously investigated with three each variants of four different kinds of host–guest complexes of varying stability. These were studied within a simulation cell containing *B*‐DNA centrally [81]. During a MD run lasting 50 ns, βCD/Q ICs adsorbed onto the DNA surface and underwent self‐aggregation. Figure S23a illustrates the optimized geometry at the end of the MD run, indicating the system's achievement of equilibrium. Figure S24 provides data on potential energy, van der Waals contribution (**S24a**) and Coulomb energy (**S24b**) contributions, over the 50 ns MD run. Consistent with previous findings, no significant conformational changes occurred between 20 to 50 ns, suggesting stable adsorption with minor local variations throughout the MD process. The concentration profiles of all atoms, calculated from 0 to 20 ns and 20 to 50 ns (Figures S24c and S24d), show nearly identical patterns, confirming equilibrium. Figure S23b details the final optimized geometry, excluding βCDs, highlighting the arrangement of quercetin molecules around the DNA: *π*‐*π* interactions involving B and A aromatic rings, and A alone, occur between two separate quercetin molecules in two different 2:2 βCD/Q ICs formed post‐adsorption. Figure S23c focuses on the drugs and βCDs in the optimized geometry without DNA and drugs. Considering the arrangement of the βCDs around the DNA architecture, the average distance between the centers of mass of six βCDs along an imaginary continuous curved is (12.8 ± 3.45) Å. The average distance between the nearest centers of mass among the six βCDs aggregated at the DNA end is (11.6 ± 2.15) Å.

Figure 2c illustrates the stable final geometry at the conclusion of a 50 ns MD simulation. It depicts the concentration profile of all atoms within the simulation cell, calculated based on this optimized geometry. Figure 3c presents the best‐fit plane for each β‐cyclodextrin (βCD). In this optimized setup, following the adsorption and self‐aggregation processes, the first aggregate aligns with the structure of the DNA, while a second aggregate forms from the aggregation initiated by the DNA's exposed bases. In both instances, combining the volume of each cyclodextrin's hydrophobic cavities with that of the hydrophobic quercetin molecules results in the formation of two hydrophobic channels adsorbed onto the DNA.

Interestingly, with host–guest complexes in 12:12 stoichiometry, the complexes partly aggregate at one end of the DNA, partly in the major groove, following the same winding direction of the double‐stranded *B*‐DNA.

For a more robust statistical analysis, as described in the preceding section, three further MD simulations were carried out under the same conditions (cell dimensions, distance‐dependent dielectric constant of water, NVT ensemble) but with different initial random arrangements of twelve βCD/Quercetin inclusion complexes around the DNA structure, which remained fixed in the simulation box. The three new initial nonoptimized geometries are illustrated in Panels **a1**, **b1**, and **c1** of Figure S25. After initial energy minimization, 50 ns MD runs, and geometry optimization of the final systems, the interaction energies between the DNA and the twelve βCD/Quercetin host–guest complexes were calculated. Considering all four optimized geometries obtained from the four MD runs (the first discussed above and the three additional simulations), the average interaction energy is equal to (−4600 ± 121.1) kJ/mol. As observed previously, βCD/Quercetin inclusion complexes with 1:1 stoichiometry are more prevalent in the final optimized geometries than those with 2:2 or 1:2 stoichiometry. A common feature of all final optimized geometries is a continuous space occupied by hydrophobic cavities and hydrophobic drug molecules aligned along the DNA structure, forming a new supramolecular complex, as illustrated in Panels **a2**, **b2**, and **c2** of Figure S25.

### 3.8 Intermolecular Interactions Between *B*‐DNA and βCD/Quercetin Host–Guest Complexes in a 16:16 Stoichiometry

Figure S26 displays the initial nonoptimized geometry of sixteen 1:1 βCD/Q ICs. These complexes, comprising four types with varying stability (Figure S3), were studied within a simulation cell containing *B*‐DNA at its center [81]. A MD simulation, lasting 50 ns, revealed the adsorption process on the DNA surface and self‐aggregation of βCD/Q ICs. Figure S27a illustrates the final optimized geometry achieved at the conclusion of an MD run, indicating the attainment of equilibrium. Figure S28 provides details regarding the potential energy, with Figure S28a highlighting van der Waals contribution and Figure S28b focusing on Coulomb energy calculations over the 50 ns MD run. Consistent with previous findings, no significant conformational changes occurred from 20 to 50 ns, indicating favorable adsorption with minor local changes over time of the duration of the MD run. The concentration profiles of all atoms calculated in the simulation cell from 0 to 20 ns and from 20 to 50 ns, as shown in Figures S28c and S28d, are nearly identical.

Figure S27b provides a detailed view of the final, optimized geometry without βCDs, illustrating the arrangement of quercetin molecules on the DNA surface. Some *π*‐*π* interactions occur involving B and A aromatic rings, as well as A alone, of two different quercetin molecules. These interactions take place within distinct 2:2 βCD/Q ICs formed after the adsorption process. Figure S27c details βCDs in the final optimized geometry, excluding DNA and drugs. The arrangement of the βCDs, consistent with the DNA architecture, shows that the average distance between the centers of mass of six βCDs, aligned along the imaginary continuous curved line is 12.8 Å. When considering the six βCDs aggregated at the end of the DNA, the average distance between adjacent centers of mass is 11.6 Å.

Figure 2d illustrates the final stable geometry at the conclusion of a 50 ns MD run, highlighting the concentration profile of all atoms within the simulation cell as calculated in the optimized geometry. Figure 3d shows the best‐fit plane for each β‐cyclodextrin. In this final geometry, following adsorption and self‐aggregation processes at the DNA ends, the aggregate partially maintains its elongated structure. The first aggregate of three βCDs exhibits an average distance between their centers of mass of (9.24 ± 1.48) Å. The second aggregate originates from the other DNA end and extends along its grooves, with an average distance between the βCD centers of mass of (14.4 ± 2.95) Å. By conceptually adding the volumes of the hydrophobic cavities of each βCD and the space occupied by the adsorbed hydrophobic drug molecules, a hydrophobic channel forms that extends variably along the DNA.

**FIGURE 4:**
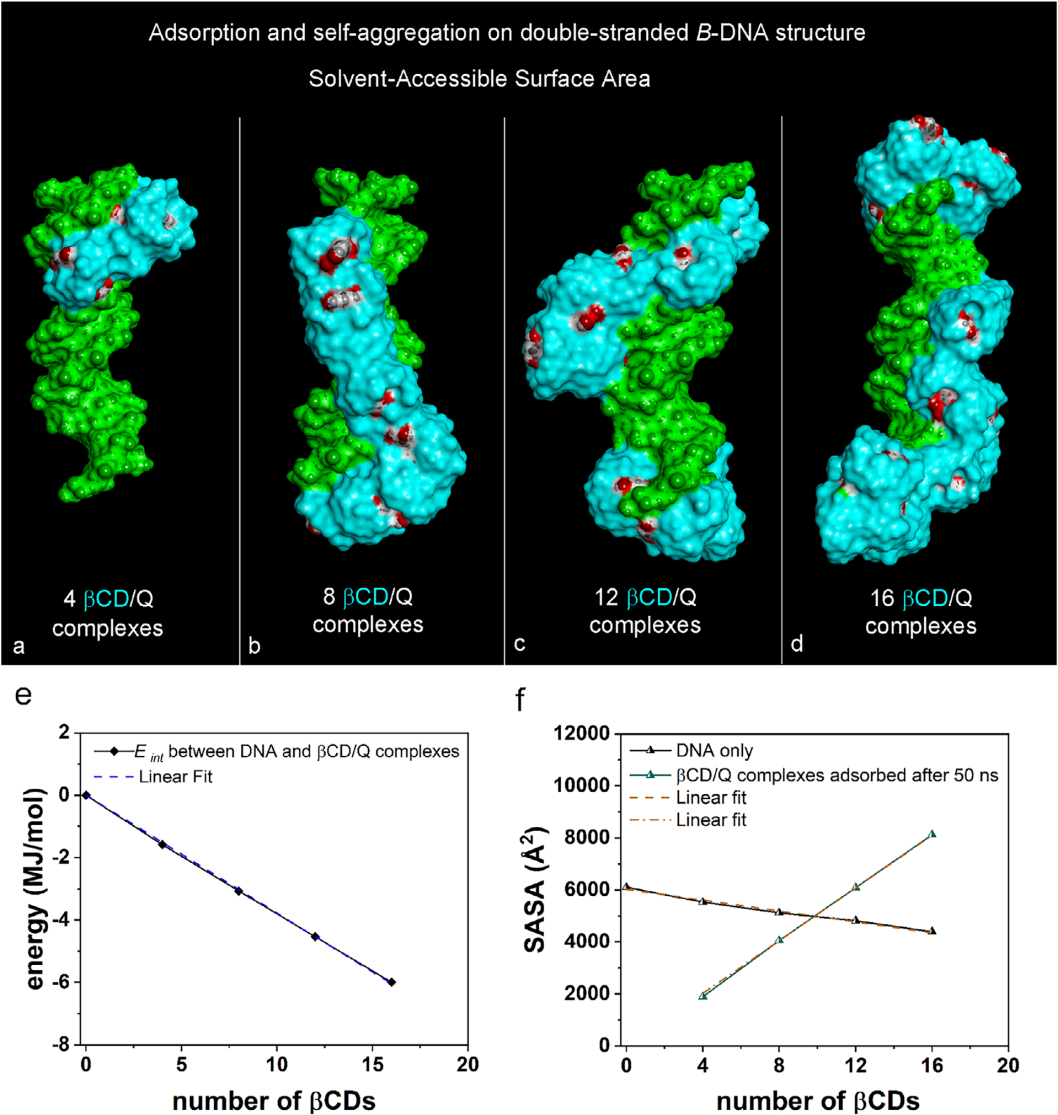
Considering the same geometries as in Figure 2, the solvent‐accessible surface area is shown colored with the color code in Figure 2, considering four, eight, twelve, and sixteen βCD/Q ICs adsorbed on DNA architecture in Panels **a**, **b**, **c**, **d** respectively. Panel **e** shows the interaction energy between DNA and βCD/Q complexes. As shown in the final optimized geometries in Figure 2, this is calculated as a function of the number of βCDs. The best linear fit indicates a linear slope: *E*_int_ = −377.5 ± 2.298 kJ/mol, *R^2^* = 0.9999. Panel **f** shows the solvent‐accessible surface area (SASA) calculated for the DNA only (lines and symbols in black, linear fit with slope equal to −104.5 ± 8.309 Å^2^) or βCD/Q inclusion complexes only (lines and symbols in green, linear fit with slope equal to 506.3 ± 3.541 Å^2^) as a function of the number of βCDs, as shown in the final optimized geometries in Figure 3.

Figure 4 illustrates the solvent‐accessible surface area (SASA) for βCD/Q ICs adsorbed on DNA architecture. Panels a, b, c, and d depict systems containing four, eight, twelve, and sixteen βCD/Q ICs, respectively, optimized after a MD run. The interaction energy between DNA and βCD/Q complexes, depicted in Figure 4e, shows a linear increase with the number of βCDs (*E*_int_ = −377.5 ± 2.298 kJ/mol, *R^2^* = 0.9999). Following the adsorption process, as shown in Figure 2, the accessible‐surface area of the DNA structure decreases linearly with increased βCD adsorption, whereas the SASA for βCD/Q complexes increases linearly (Figure 4f). Notably, systems with twelve, and sixteen adsorbed complexes exhibit a higher SASA than the isolated DNA fragment. In the configurations shown in Figure 2, some encapsulated quercetin molecules are more exposed to the biological environment, as indicated by their red‐highlighted oxygen atoms. It is crucial to emphasize this aspect, as subsequent studies focus on quercetin adsorption on DNA without cyclodextrins. Here, quercetin molecules preferentially adsorb in the major grooves due to favorable intermolecular interactions. They are distributed more uniformly across the DNA double helix and are partially exposed to the environment, facilitated by βCDs. In the host–guest complexes with a 12:12 stoichiometry, the complexes aggregate at the DNA's ends and align along the B‐DNA structure, adsorbing in minor and major grooves and on exposed phosphate groups. Some quercetin molecules also partially intercalate into minor grooves.

The DNA/βCD/Q supramolecular structure formed after MD runs, shows that βCD/Q ICs partially cover DNA's negatively charged phosphate groups. These groups often hinder DNA's passage through plasmid membranes due to electrostatic repulsion with the cell membrane. In gene delivery, introducing exogenous nucleic acids into cells is crucial for controlling cellular behavior. Both viral or nonviral gene delivery vectors can facilitate the deliberate transfection of nucleic acids into eukaryotic cells. Supramolecular complexes, such as polyplexes, play a significant role for this process. In the case studied, the ICs adsorb onto the DNA structure, enabling partial coverage through intermolecular interactions on its external surface. Depending on their concentration, βCD/Q ICs could improve the passage through the plasma membrane, thereby enhancing the drug release within the cell. However, optimizing concentration is always critical to avoid cytotoxic effects.

### 3.9 Intermolecular Interactions Between *B*‐DNA and Quercetin Drug Molecules at Different Concentrations

Following the procedure detailed in the *Materials and Methods* section, we initially investigated the interaction energy between the quercetin and the minor and major grooves of DNA using a strategy previously adopted [81]. We minimized the energy of the final geometries obtained from four MD runs. These runs began with quercetin positioned either parallel or perpendicular to the DNA's minor or major grooves, optimizing all frames saved every 2 ns. This approach enabled the exploration of both stable and metastable interaction geometries.

From these four initial geometries, the MD runs resulted in two final optimized geometries where the quercetin molecule was adsorbed in the major groove of the DNA. The interaction energy for this configuration was notably favorable, calculated at −615.0 kJ/mol (geometry 3 in Figure S29). Additionally, after 10 ns, two geometries were observed with quercetin molecules in the minor groove, yielding an interaction energy less stable about than 26 of −589.0 kJ/mol (geometry 1 in Figure S29).

A favorable DNA/quercetin interaction energy was particularly evident when the drug interacts with the major groove. Notably, quercetin exhibited greater mobility in the major groove compared to the minor groove. Figure S29e illustrates the calculated distance between the quercetin center of mass and the axis along which the DNA fragment unfolds during the four 10 ns MD runs, alongside the corresponding distributions in Figure S29f. This freedom of motion is further displayed in the animations below, Figure S29.

The potential energy and van der Waals contribution calculated during a 10 ns MD run are presented in Figure S30. Adsorption occurred more rapidly in the major groove, facilitated by favorable van der Waals interactions.

**FIGURE 5:**
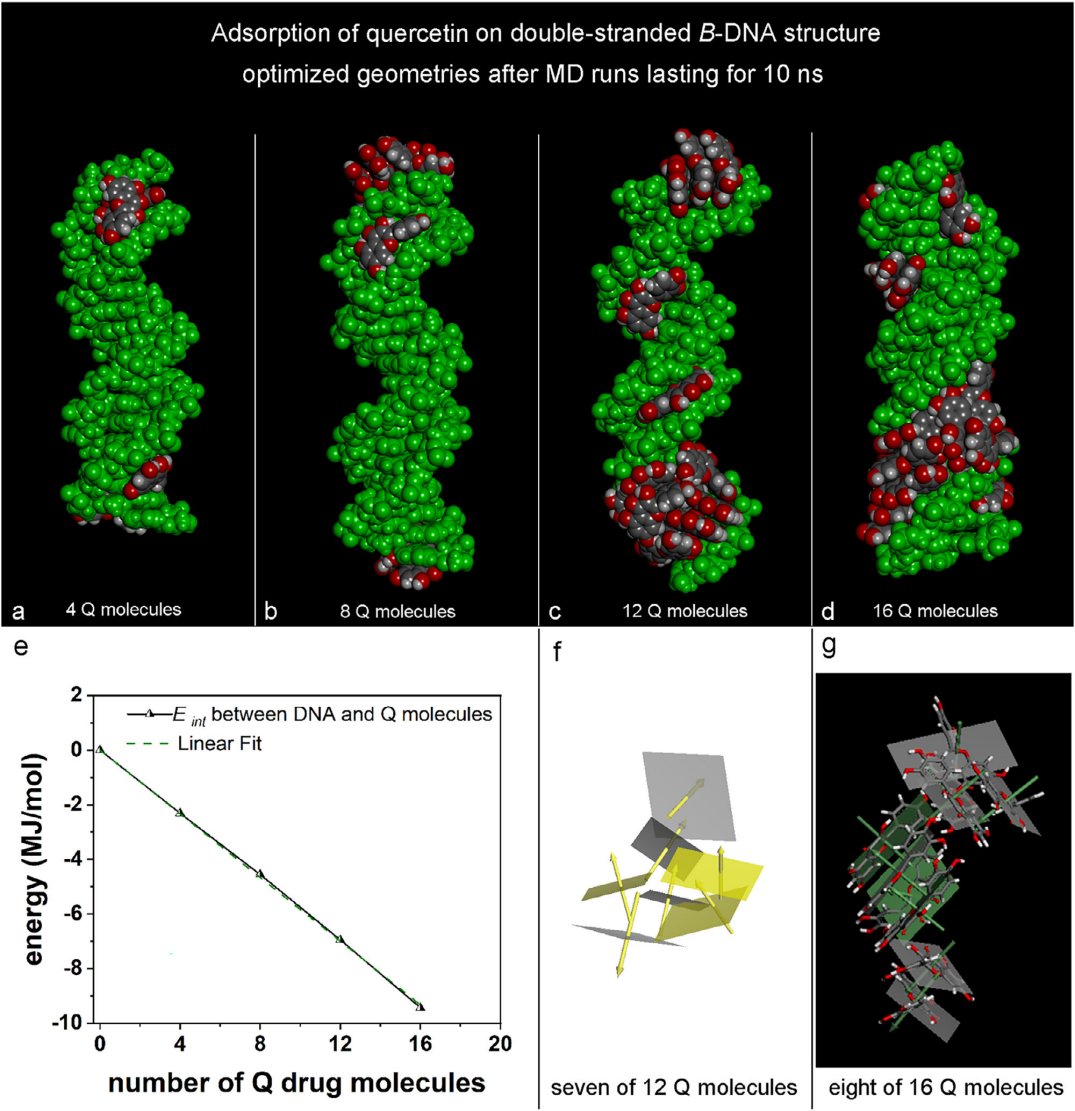
Side view of the optimized geometries obtained after MD runs lasting 10 ns related to the adsorption and self‐aggregation processes of four, eight, twelve, and sixteen quercetin molecules on double‐stranded DNA (Panels **a**, **b**, **c**, and **d**, respectively). The color code is the same as in Figure 2. In Panel **d**, a detail of the quercetin molecules adsorbed in a major groove and aggregated following the helicoidal architecture is shown. The best‐fit plane for each molecule is shown. The interaction energy between the DNA and Q molecules as a function of the number of drug molecules is shown (Panel **e**). The best linear fit indicates a linear slope: *E*_int_ = −583.3 ± 3.546 kJ/mol, *R^2^* = 0.9993. Panel **f** shows a detail of 7 of 12 quercetin molecules with the best‐fit plane for each molecule in yellow. Panel **g** shows eight of sixteen quercetin molecules adsorbed in contact with the major groove of the DNA, with its best‐fit plane and drug molecules in stick representation.

The study investigated the adsorption and self‐aggregation processes of quercetin molecules at higher concentrations within the simulation cell, maintaining previously established concentrations. The research examined four, eight, twelve, and sixteen drug molecules initially randomly around the central DNA segment. Panels **a**, **b**, **c**, and **d** in Figure 5 reveal that at higher concentrations, some quercetin molecules adsorb at the DNA ends, interacting with bases exposed by the DNA fragment. A few quercetin molecules adsorb within the minor groove, while a greater number self‐aggregate in the major groove. The interaction energy is higher in the major groove when considering a single drug molecule, consistent with the DNA's structural features. Figure 5e depicts the interaction energy between of the DNA and quercetin molecules as a function of the number of drug molecules. The best linear fit, which passes through the origin, shows a linear slope of −583.3 ± 3.546 kJ/mol (*R^2^* = 0.9993). Panel **f** highlights seven of twelve quercetin molecules with their respective best‐fit planes marked in yellow. Panel **g** displays eight of sixteen quercetin molecules adsorbed along the major groove of the DNA, depicted in stick representation with their best‐fit planes. Notably, this configuration demonstrates an evident ordering of the quercetin molecules along the helical path of the DNA's major groove.

Comparing the adsorption geometries in Figures 5 and 2, it is possible to consider cyclodextrins as carriers which are capable of dispersing the drug molecules in a more homogeneous and ordered way along the unwinding of the DNA helix, maintaining the included hydrophobic drugs on average further away than in the situation without cyclodextrin carriers.

### 3.10 Intermolecular Interactions Between *B*‐DNA and βCDs at Different Concentrations

Following the procedure outlined to study the intermolecular interactions between a βCD molecule and double‐stranded *B*‐DNA, the final adsorption process was examined, starting from the six different initial geometries described in the *Materials and Methods* section [81]. The final adsorption geometries, obtained after MD simulations of 10 ns, are depicted in Figure S31. The potential energy and the van der Waals contributions calculated during MD runs extending to 20 ns are shown in Figure S32. Interaction energies for the various optimized geometries are presented in Table S4, along with the number of DNA/βCD intermolecular hydrogen bonds and βCD intramolecular hydrogen bonds.

The most favorable interaction occurred when the βCD secondary rim engaged with both the negatively charged oxygen of phosphate groups and the DNA bases in the major groove, as indicated in geometry 6 of Table S4. A metastable geometry, less stable of 22.5 kJ/mol, was found when the βCD primary rim interacts with the DNA in the major groove. During MD simulations, βCD demonstrated considerable freedom of movement around the DNA, located in either the minor or major groove, as illustrated in the animations following Table S4. Notably, over time, not both the hydroxyl groups of βCD and its cavities exhibited strong interactions with the negatively charged oxygen of the phosphate groups along the DNA structure.

**FIGURE 6:**
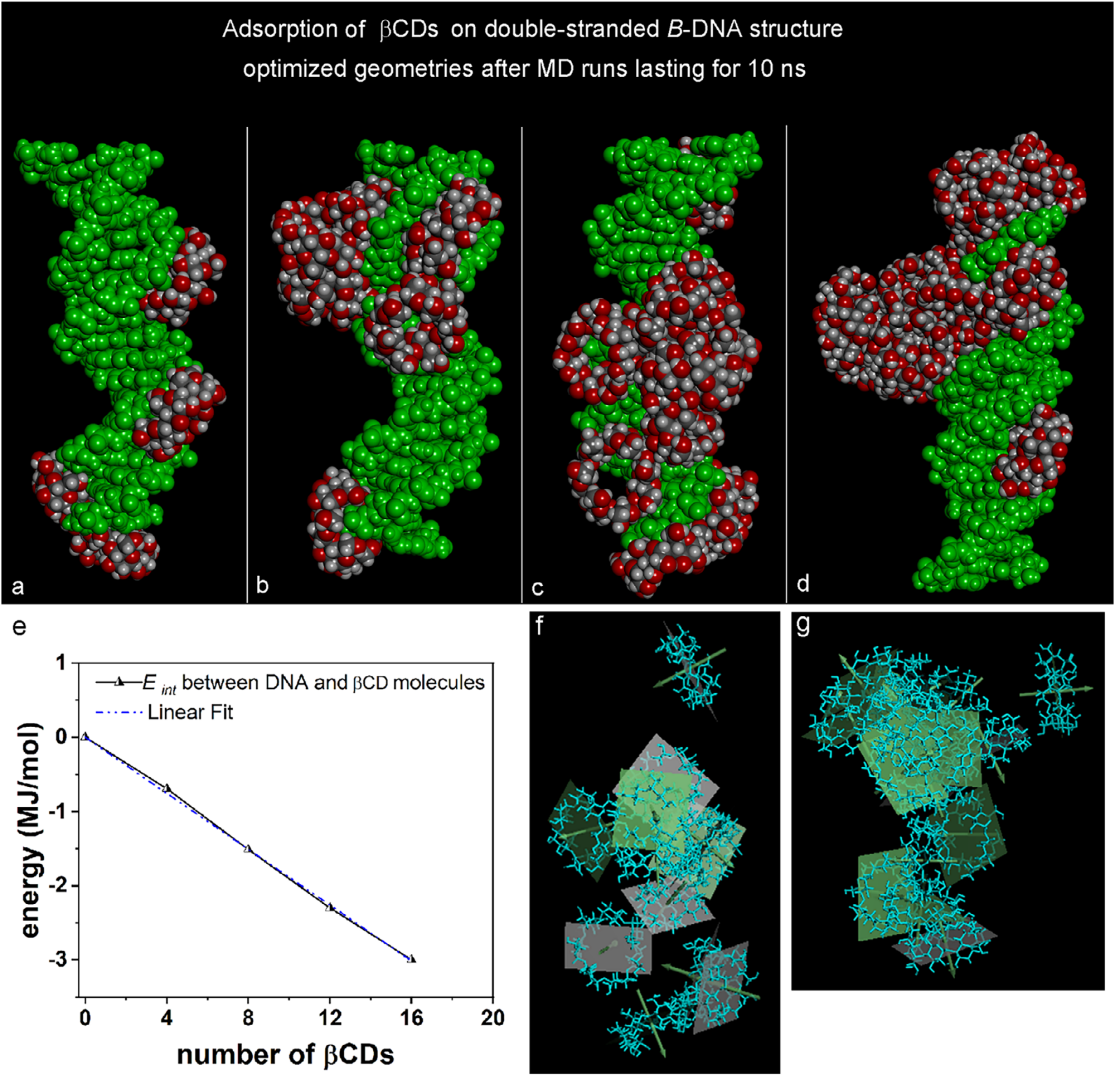
Side view of the optimized geometries obtained after MD runs lasting 20 ns related to the adsorption and self‐aggregation processes of four, eight, twelve, and sixteen βCD molecules on double‐stranded DNA (Panels **a**, **b**, **c**, and **d**, respectively). The color code is the same as in Figure 2. Panel **e** shows the interaction energy between the DNA and βCD molecules adsorbed on the DNA structure calculated as a function of the number of βCDs for the same optimized geometries in Panels **a**, **b**, **c**, and **d**. The best‐fit plane for each molecule is shown. The best linear fit indicates a linear slope: *E*_int_ = −188.5 ± 1.744 kJ/mol, *R^2^* = 0.9996. Panels **f** and **g** show a detail of 12 and 16 βCDs with only the best‐fit plane for each molecule without the DNA surface. βCD molecules and their best‐fit planes are in light blue in stick representation.

Following the simulation protocol previously used for quercetin molecules, we investigated the adsorption and self‐aggregation processes of βCD molecules at higher concentrations within the simulation cell, similar to that was utilized for studying ICs We examined configurations with four, eight, twelve, and sixteen drug molecules, initially arranged randomly around the DNA in its central region. Panels **a** through **d** in Figure 6 illustrate that at higher concentrations, some βCD molecules adsorb at the DNA ends, interacting with exposed bases. Other βCDs molecules adsorb in the minor or major grooves, interacting through their cavities with the phosphate groups exposed by the DNA structure. Notably, with twelve or sixteen βCDs molecules, self‐aggregation occurred along the DNA's axis or its major grooves, resulting in a larger aggregate adsorbed perpendicular to the DNA axis. Figure 6e presents the interaction energy between DNA and βCD molecules as a function of the number of βCD molecules. The best linear fit, which passes through the origin, indicates a linear slope of −188.5 ± 1.744 kJ/mol (*R^2^* = 0.9996). Panels **f** and **g** in Figure 6 provide detailed of twelve and sixteen βCDs, showing only the best‐fit plane for each molecule, excluding the DNA surface for clarity.

The interaction between DNA and β‐cyclodextrins (βCDs) becomes more favorable when quercetin molecules are included within their cavities, creating the aforementioned hydrophobic channel. Panel **f** illustrates twelve βCD molecules enveloping the DNA surface, with the best‐fit plane for each molecule displayed. The DNA structure is omitted for clarity. In panel **g**, sixteen βCD molecules are shown adsorbed along the major groove of the DNA, again without the DNA structure to enhance clarity. This depiction emphasizes the aggregation of βCD within the DNA's major.

### 3.11 Intermolecular Interactions Between βCDs at Different Concentrations

**FIGURE 7:**
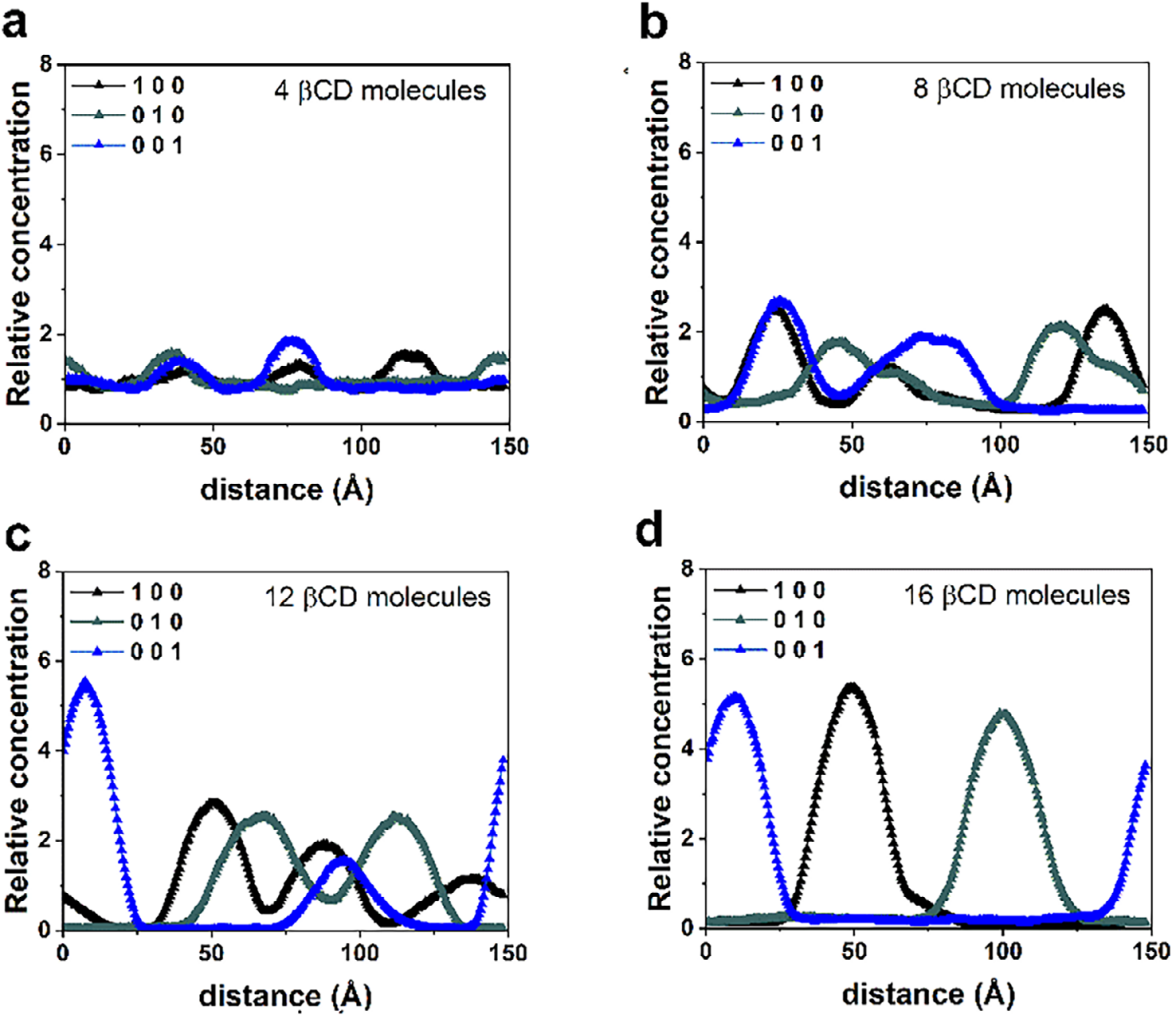
Panels **a**, **b**, **c**, **d** show the relative concentration calculated for the atoms of four, eight, twelve, and sixteen βCD molecules during MD runs lasting 20 ns (animations following Figure S36) starting from the geometries shown in Figure S38.

We investigated the βCD aggregation process using MD simulations, following the procedure outlined in the *Materials and Methods* section [82, 83]. Starting with initial geometries of four, eight, twelve, and sixteen βCDs arranged randomly within a simulation cell (Figure S33), we conducted MD runs for 20 ns. The potential energy and van der Waals contributions calculated during these runs are presented in Figure S34. The relative concentrations of all βCDs in the simulation cell, determined across the four different MD runs, are depicted in Figure 7 Panels **a** through **d**. Figure S35 illustrates the SASA, color‐coded by atoms, of all the βCDs in the optimized geometries obtained after 20 ns MD runs.

During the MD simulations, βCDs interacted and self‐aggregated, forming a supramolecular complex that changed dynamically over time. Increasing the number of βCDs resulted in more stable complexes with an elongated shape. This was attributed to favorable intermolecular interactions between different rims and surface interactions on the βCD macrocycle. In some instances, the hydroxyl groups of primary rims became incorporated into the central cavity of another βCD. At low concentrations, an equilibrium between aggregated and isolated βCD molecule was observed. In contrast, at larger concentrations, aggregation into larger structures occurred dynamically, as demonstrated in the supplementary animation beneath Figure S35. When comparing these βCD aggregates with and without double‐stranded DNA at the same concentration, the DNA aligns the βCD structure. The influence of quercetin on the βCD aggregation process is further explored in the final paragraph.

### 3.12 Intermolecular Interactions Between βCDs and Quercetin Molecules at Different Concentrations

Following the simulation protocol detailed at the end of *Materials and Methods* section, we examined the intermolecular interactions between βCDs and quercetin molecules in identical and varying concentrations, previously studied in the presence of double‐stranded DNA, now without DNA and any preformed ICs. The initial geometries, featuring βCDs and quercetin molecules in a random arrangement within a simulation box, are depicted in Figure S36. The potential energy and the van der Waals contributions calculated during MD runs, are presented in Figure S37. Animation files of the MD runs, which lasted 20 ns, are included in the Supplementary Information below Figure S37.

The favorable van der Waals intermolecular interactions drive the aggregation process, resulting in the formation of elongated, flexible supramolecular structures that stabilize over time by increasing the number of βCD and quercetin molecules. Notably, these aggregates form different βCD/Q ICs with stoichiometries 1:1, 2:1, 1:2, and 2:2 (Figure S38). The SASA of the optimized geometries obtained at the conclusion of the MD runs is presented in Figure S38. Interestingly, the encapsulated quercetin molecules are only partially exposed by βCDs and are arranged specifically within the hydrophobic cavities of βCDs.

Figure S39 provides a detailed view of drug molecules within the optimized aggregate. When analyzing four βCDs and quercetin molecules (Panel S38a), three drug molecules exhibit center of mass positioning that resembles the vertices of an almost equilateral triangle, with edges about 9.65 Å in length. In studies involving eight, twelve, and sixteen βCDs and quercetin molecules (Panels S38b, S38c and S38d), the drug molecules become ordered due to *π‐π* interactions. However, they do not form a crystalline, closely packed, but instead interact in space in an orderly arrangement. Instead, these molecules interact orderly within the hydrophobic cavities of βCD, aligning in a preferred direction akin to a mesophase director. Over time, a new ordered system emerges, where drug molecules are more distantly placed, indicative of their solid‐state arrangement. Weak van der Waals interactions at relatively short distances are enhanced by the βCDs’ hydrophobic cavities, facilitating contact and interaction transmission in a hydrophobic space among drug molecules. The incomplete enclosure of drug molecules permits relative mobility, allowing them to interact with each other, with mobility influenced by the simulation temperature.

**FIGURE 8:**
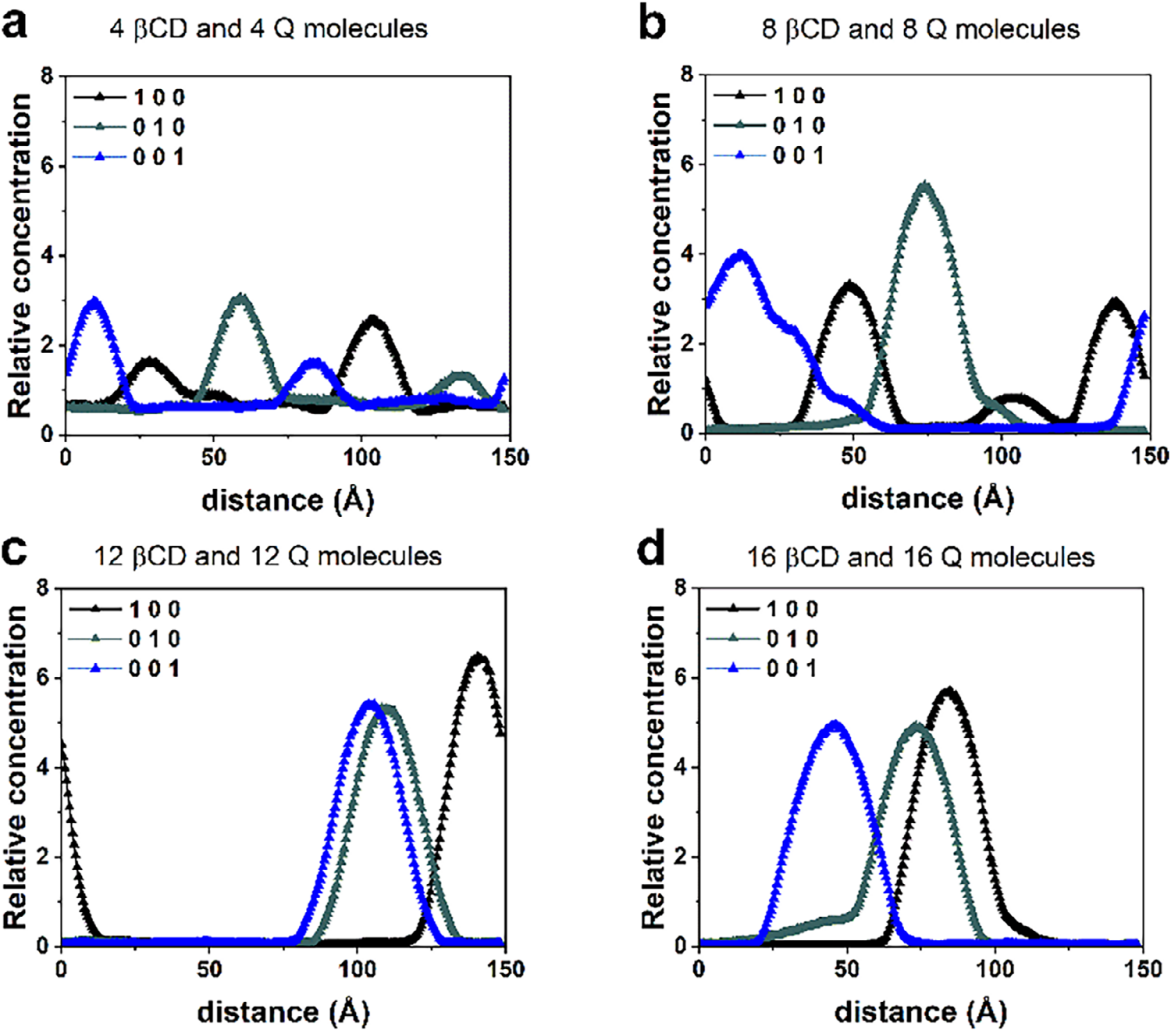
Panels **a**, **b**, **c**, **d** show the relative concentration calculated for the atoms of four, eight, twelve, and sixteen βCD and quercetin molecules calculated during MD runs lasting 20 ns (animations following Figure S36) starting from the geometries shown in Figure S33.

The relative concentration of all βCDs and quercetin molecules within the simulation cell, as determined over four different MD runs, is depicted in Figure 8 across Panels **a**, **b**, **c**, and **d**. When comparing these concentrations to those calculated without quercetin molecules, the role of the drug in this aggregation process is notably distinctive. The formation of stable aggregates over time becomes more favorable not only by increasing the number of βCDs, but also through the inclusion of quercetin molecules. These hydrophobic drugs function as a binding agent for cyclodextrins, leading to stable aggregates that are more or less elongated in one direction.

**FIGURE 9:**
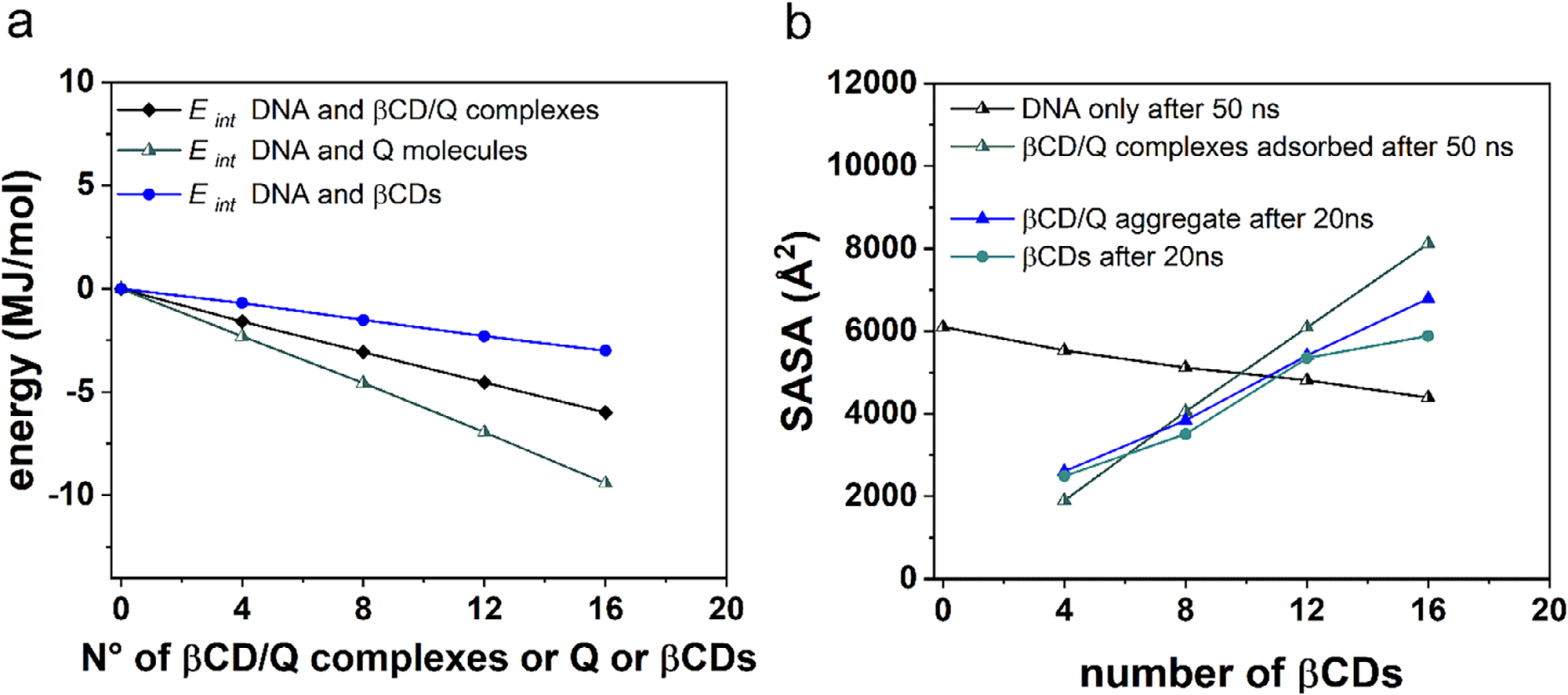
Panel (**a**) Interaction energy between DNA and βCD/Q molecules, DNA and Q drug molecules, DNA and βCD molecules adsorbed on the DNA structure calculated as a function of the number of βCD/Q complexes, or Q drug or βCD molecules, as shown in Panel **6** of Figures 4, 5, and 6, respectively. Panel (**b**) SASA calculated for DNA only and βCD/Q complexes adsorbed on the DNA surface in the same optimized geometries as shown in Figure 2, together with the SASA calculated for the βCD/Q aggregate after an MD run in a simulation cell without DNA (as shown in Figure S38) and for βCDs studied in the same concentration in a simulation cell (as shown in Figure S35).

Figure 9a illustrates the interaction energy calculations for three scenarios: between the DNA and βCD/Q molecules (previously referenced in Figure 4, Panel 6), between DNA and quercetin drug molecules (Figures 5, Panel 6), and between DNA and βCD molecules adsorbed onto the DNA structure (Figure 6, Panel **e**). These interactions are analyzed based on the number of βCD/Q complexes, quercetin drug, or βCD molecules. The results demonstrate a linear relationship. Notably, the interaction energy with double‐stranded DNA decreases when considering an equivalent number of quercetin molecules, βCD/Q ICs, and βCD molecules.

The hydrophobic drug continues to play an important role among cyclodextrins, as well as in the adsorption process on the DNA structure.

In the presence of the hydrophobic quercetin molecule, adsorption occurs due to more favorable van der Waals interaction when considering only the carrier molecule, βCD. Figure 9b illustrates the SASA calculated for the DNA only and βCD/Q complexes adsorbed on the DNA surface in optimized geometries (Figure 2). Additionally, it shows SASA calculations for βCD/Q complexes post‐MD simulations in a cell without DNA (Figure S38) and for βCDs in the same concentrations in a simulation cell (Figure S35). Following the adsorption of ICs, the DNA SASA expectedly decreased. Interestingly, the βCD SASA at identical concentrations, but with encapsulated drugs, expanded due to the presence of the drug itself, increasing surface area exposed to the solvent. This effect was even more pronounced when the complexes were organized on the DNA structure, leading to greater swelling.

It is crucial to highlight the role of β‐cyclodextrins (βCDs) as carriers of hydrophobic drugs, facilitating their solubilization following adsorption onto DNA. The structure of DNA, which presents negatively charged phosphate groups, is partially shielded by hydrophilic aggregates formed through ICs with these hydrophobic drugs. These aggregates are both exposed to the biological environment and intercalated into the DNA's minor grooves. This mechanism is significant for the gene delivery challenge, as it may allow DNA to permeate the cell membrane with its negative charges partially concealed by adsorbed drug molecules. If DNA can traverse the membrane, the drugs could be effectively delivered into the cells.

## 4 Conclusion

β‐cyclodextrins (βCDs) and quercetin drug molecules, a flavonoid with anti‐inflammatory, antioxidant, and anticancer properties, form stable inclusion complexes (ICs) with various stoichiometries. The intermolecular interactions between the hydrophobic cavities of βCDs and quercetin play a crucial role when these inclusion complexes adsorb and self‐assemble on double‐stranded DNA structure. MD simulations show that these host–guest complexes wrap along the DNA surface, forming an ordered, long‐range hydrophobic channel. Hydrogen bonds stabilize this supramolecular structure around the DNA over time.

The concentrations of both the drugs and its carriers influence the process of adsorption and self‐assembly. The ICs adhere via cooperative dynamic effects, where drugs encapsulated or partially included within the βCD cavities exhibit short‐range sliding motions, aided by *π*‐*π* interactions. Consequently, the cyclodextrins align along the DNA structure, their hydrophobic cavities forming long‐range ordered mesogenic clusters in the DNA grooves. The interaction culminates in a new supramolecular structure.

The alignment of DNA, βCD, and quercetin molecules aligned along the DNA axis resembles an ivy vine climbing a tree trunk.

Classical oligomeric and polymeric systems, whether linear or cyclic and liquid or plastic crystalline forms, typically consist of mesogenic groups that establish spatial and temporal order. These systems also contain a flexible spacer, which, due to its flexibility and mobility, tends to disorder and/or dynamically orient the system both spatially and temporally. The hydrophobic cavities of cyclic β‐cyclodextrins (βCDs) can be conceptualized as mesogenic volumes. These volumes are interconnected over time by short‐range drug‐on‐drug sliding motions facilitated by *π*‐*π* interactions, acting as a flexible thread of intermolecular interactions. These interactions occur between hydrophobic drugs that are included (or partially included) in the cavities of the inclusion complexes (ICs). Such hydrophobic interactions organize the supramolecular structure of the ICs when adsorbed onto double‐stranded DNA, aligning according to the DNA's direction, aided by a continuous series of hydrogen bonds among cyclodextrins. In the literature, these noncovalent interactions among CDs have been documented both in solution and in the solid state, with cyclodextrin aggregates forming columnar phases in concentrated solutions [95, 96].

The plasticity of supramolecular nanoaggregates is intriguing, necessitating an analysis over time to assess potential changes in their overall shape, which can vary from spherical and isotropic to ellipsoidal and anisotropic. Additionally, examining the mobility of the molecules that contributing to the formation of these nanoaggregates is of interest. The adsorption process is the initial stage in the interaction between β‐cyclodextrin (βCD) and DNA. This stage involves ordered self‐aggregation on the DNA surface and precedes the intercalation process of the partially encapsulated drug. Over time, this drug migrates from the hydrophobic cavity of the βCD toward the DNA, illustrating the drug release process. Further simulations, conducted in an aqueous environment, will provide insight into the release mechanism over time [84, 85], and the tendency of inclusion complexes (ICs) to cluster in water. As Chandler discusses [86], when the number (n) of solutes—representing in this case the number of ICs or hydrophobic drug molecules—is sufficiently large, these solutes can form a cluster with a large enough volume‐to‐surface ratio. This ratio results in a solvation free energy lower than the overall solvation free energy of the individual solutes, providing a favorable driving force for cluster assembly. Since the interface cost increases linearly with surface area, accurately calculating the surface‐accessible surface area is crucial, as addressed in the present work. Chandler also notes that a cluster can be stable or metastable only if it exceeds a critical size, highlighting the importance of studying IC aggregation at varying concentrations of hydrophobic drug molecules or ICs in general [87, 90, 93]. The geometry of interaction, particularly the arrangement of guest molecules within inclusion complexes, influences the stability of either stable or metastable ICs. Interestingly, metastable ICs may release encapsulated drugs more rapidly than their more stable counterparts, as discussed in previous theoretical and current experimental studies [87, 88, 89, 90, 91, 92, 93, 94].

Supramolecular chemistry facilitates the creation of highly complex chemical systems using molecular components linked by noncovalent intermolecular forces [97]. This study proposes a theoretical procedure to examine the formation and stability of long‐range ordered supramolecular nanostructures along DNA over time [81]. MM/MD simulations offer valuable insights into the dynamic stability, assessed at an atomistic level. This stability arises from the synergy of weak, yet numerous, anisotropic, and spatially ordered intermolecular forces within an extensive network of hydrophobic interactions and hydrogen bonds. In this context, ICs for drug delivery on the DNA architecture create a relatively long hydrophobic channel.

Notably, the theoretical findings align with experimental data presented by Shumyantseva et al. Using electrochemical methods to analyze quercetin interactions with DNA, they determined a binding constant of 4.8 × 10^3^ M^−1^. They identified binding in DNA grooves and the electrostatic interactions as the types of quercetin interactions. The research also emphasized the significance of drug concentration on the intensity of electrochemical oxidation of DNA bases [64]. This theoretical work investigates not only the noncovalent interactions of quercetin with DNA structures but also the adsorption and self‐aggregation of various ICs along the DNA axis. The concluded supramolecular structure theoretically involves interactions between ICs, DNA grooves, and phosphate groups. These intermolecular interactions along the DNA architecture are crucial for the stability of ICs/DNA supramolecular structures. Consequently, they are spatially and temporally organized to form a hydrophobic channel, integrating hydrophobic βCD cavities with well‐ordered hydrophobic interacting drug molecules.

The role of drug and IC concentrations requires further investigation, particularly when there is a strong drug/carrier interaction. Overcoming this interaction in supramolecular complexes in vivo is essential. Alghmandi et al. [59] discuss such challenges in the case of curcumin, which demonstrates the pharmacokinetic and pharmacodynamic issues to its slow or incomplete release from the complex.

One intriguing aspect of cyclic oligosaccharides, such as β‐cyclodextrin (βCD) cavities, is their ability to act as a ‘glue’ between different hydrophobic quercetin molecules. The hydrophobic interactions create a region occupied not only occupied by atoms of these molecules but also by empty, hydrophobic space. Similarly, drug molecules can serve as a ‘glue’ for βCDs. In the presence of quercetin molecules, βCD aggregates form more quickly and remain stable over time than when in the case of pure βCDs. This formation creates a hydrophobic channel aligned along a single line, reminiscent of the hydrophobic cross‐linked cyclodextrin‐based metal–organic framework investigated by Zhao et al. This framework is notable for its potential application in controlled release as a novel nutritional delivery system within the food and biomedical sectors [55].

An ordered hydrophobic continuous channel with drugs on elongated DNA constitutes a novel supramolecular structure. This complex forms following the adsorption and self‐aggregation of βCD/Q complexes on the DNA structure, resulting in a favorable solvent‐accessible surface area with fewer negative charges compared to DNA exposed to a biological environment. Given the significance of polyplexes for gene delivery, it is plausible to hypothesize that this new supramolecular structure could facilitate passage through the plasma membrane by shielding the negative charges of DNA phosphate groups, thereby enhancing drug release within the cell.

Hydrophobic drugs encapsulated in β‐cyclodextrins (βCDs) are promising agents due to their ability to swell, facilitated by cooperative hydrophobic interactions. Host–guest complexes, in particular, hold potential as non‐viral vectors for nucleic acids. Additionally, when these complexes adsorb onto the surface of a double‐stranded DNA, the DNA can function as a vector for hydrophobic drugs encapsulated in the βCD cavities or adsorbed within its grooves. This can be visualized as a train with an additional carriage, complementing its genetic cargo. These components are interconnected through numerous weak and extend across cell membranes in a specific direction.

DNA‐based nanomaterials with advanced nanostructures have been developed, significantly expanding the applications of DNA [24, 25, 26, 27, 28]. This theoretical study offers insights into the concept of a supramolecular hydrophobic channel encasing DNA. Such a channel could enhance applications in drug and gene delivery. The study emphasizes the necessity for multiscale investigations and experiments to fully comprehend these systems.

## Conflicts of Interest

The authors declare no conflicts of interest.

## Supporting information



Supplementary figures, graphs, and tables, related to the βCD/quercetin inclusion complexes in 1:1 and 2:1 stoichiometries.
Supplementary figures and graphs related to the intermolecular interactions of *B*‐DNA and βCD/Q host–guest complexes in 4:4, 8:8, 12:12, and 16:16 stoichiometries.
Supplementary figures, graphs, tables, and animation file related to intermolecular interactions between *B*‐DNA and a single quercetin drug molecule, and between *B*‐DNA and 4, 8, 12, and 16 Q drug molecules.
Supplementary figures, graphs, and animation file related to intermolecular interactions between *B*‐DNA and 4, 8, 12, and 16 βCD molecules.
This material is available free of charge via the Internet at http://pubs.acs.org.
**Supporting File**: chem70786‐sup‐0001‐SuppMat.pdf.

## Data Availability

The data that support the findings of this study are available from the corresponding author upon reasonable request.
