# Supplementary material for: DNA as Drug Carrier: A Hydrophobic β‐Cyclodextrin Channel in a Supramolecular Structure
Source: Chemistry. 2026 Feb 15;32(10):e02895. doi: 10.1002/chem.202502895 (PMC12995853; doi:10.1002/chem.202502895)
Supplement: Supplementary file 1 — Supplementary figures, graphs, and tables, related to the βCD/quercetin inclusion complexes in 1:1 and 2:1 stoichiometries. Supplementary figures and graphs related to the intermolecular interactions of B‐DNA and βCD/Q host–guest complexes in 4:4, 8:8, 12:12, and 16:16 stoichiometries. Supplementary figures, graphs, tables, and animation file related to intermolecular interactions between B‐DNA and a single quercetin drug molecule, and between B‐DNA and 4, 8, 12, and 16 Q drug molecules. Supplementary figures, graphs, and animation file related to intermolecular interactions between B‐DNA and 4, 8, 12, and 16 βCD molecules. This material is available free of charge via the Internet at http://pubs.acs.org. Supporting File: chem70786‐sup‐0001‐SuppMat.pdf. [file CHEM-32-e02895-s001.pdf]

Supplementary Materials

DNA as Drug Carrier: a Hydrophobic β-Cyclodextrin Channel in a Supramolecular Structure

Giuseppina Raffaini†*

† Department of Chemistry, Materials, and Chemical Engineering "Giulio Natta", Politecnico di Milano, Piazza L. Da Vinci 32, 20131 Milano (Italy).
* Correspondence: giuseppina.raffaini@polimi.it

Dedicated to Professor Fabio Ganazzoli and Professor Giuseppe Resnati on occasion of their 70th birthday.

Received: date; Accepted: date; Published: date

Structure of βCD and Quercetin

Figure S1. Panel **a** illustrates the side view and top view of the βCD optimized geometry, with indication of the primary and secondary rims. On the left, the plane of best fit and the arrow pointing towards the secondary rim; below, the βCD c.o.m. Panel **b** displays the optimized geometry of quercetin, with indication of the A, B and C rings (discussed later in the article) and the structure with its c.o.m. Panel **c** shows the double-stranded DNA structure in a CPK representation. βCD and the drug molecule are represented by sticks. Color code: carbon atoms are in gray; oxygen in red; nitrogen in blue; and hydrogen in white……..S8

Table S1. Intramolecular H-bonds in the optimized β-cyclodextrin and quercetin molecule……………….S8

Intermolecular Interactions Between βCDs and Quercetin Drug Molecules

Inclusion Complexes of βCD/Q in a 1:1 Stoichiometry

Figure S2. Side view of the initial *non-optimized* geometries of β-cyclodextrin and quercetin in a 1:1 stoichiometry with the drug molecule initially parallel or perpendicular to the βCD primary rim (Panels **a1**, **b1**, and **c1**, respectively) and to the βCD secondary rim (Panels **a2**, **b2**, and **c2**, respectively), without any inclusion a priori. The color code is the same as in Figure **S1**……………………………………….……S9

Figure S3. Side view of the initial optimized geometries of β-cyclodextrin and quercetin in a 1:1 stoichiometry with the drug molecule initially parallel or perpendicular to the βCD primary rim (Panels a1, b1, and c1, respectively) and to the βCD secondary rim (Panels a2, b2, and c2, respectively). The color code is the same as in Figure S1. When the drug inclusion process takes place, all atoms of βCD are in green……………………………………………………………………………………………………...S10

Table S2. Interaction energy (kJ/mol), intramolecular H-bonds in βCD and quercetin molecules, and βCD/drug intermolecular H-bonds in the **initial optimized geometries** shown in Figure **S3**…………..…S10

Figure S4. Side view of the final optimized geometries after an MD run lasting 5 ns of β-cyclodextrin and quercetin in a 1:1 stoichiometry starting from the geometries in Panels **a1**, **b1**, **a2**, and **b2**, respectively. The color code for the quercetin molecule is the same as in Figure **S1**; all atoms of βCD are in green………...S11

Table S3. Interaction energy (kJ/mol), intramolecular H-bonds in βCD and quercetin molecules, and βCD/drug intermolecular H-bonds in the final optimized geometries after a 5 ns MD run as shown in Figure S4……………………………………………………………………………………………………S11

Figure S5. Panels **a** and **b** show the potential energy, van der Waals contribution and Coulomb energy calculated during an MD run lasting 5 ns starting from the geometry in Panel **a1** of **Figure S4 (geometry P1** in **Table S3)**. Panels **c** and **d** display the distance between the βCD and the quercetin center of mass (c.o.m.), and the distribution of these distances calculated during the MD run, respectively………………S12

Figure S6. Panels **a** and **b** show the potential energy, van der Waals contribution and Coulomb energy calculated during an MD run lasting 5 ns starting from the geometry in Panel **b1** of **Figure S4 (geometry P2** in **Table S3)**. Panels **c** and **d** display the distance between the βCD and the quercetin center of mass (c.o.m.), and the distribution of these distances calculated during the MD run, respectively…………..…S12

Figure S7. Panels **a** and **b** show the potential energy, van der Waals contribution and Coulomb energy calculated during an MD run lasting 5 ns starting from the geometry in Panel **a2** of **Figure S4 (geometry S1** in **Table S3)**. Panels **c** and **d** display the distance between the βCD and the quercetin center of mass (c.o.m.), and the distribution of these distances calculated during the MD run, respectively………………S13

Figure S8. Panels **a** and **b** show the potential energy, van der Waals contribution and Coulomb energy calculated during an MD run lasting 5 ns starting from the geometry in Panel **b2** of **Figure S4 (geometry S2** in **Table S3)**. Panels **c** and **d** display the distance between the βCD and the quercetin center of mass (c.o.m.), and the distribution of these distances calculated during the MD run, respectively……………....S13

Inclusion Complexes of βCD/Quercetin in a 2:1 Stoichiometry

Figure S9. Panels **a** and **b** show the initial non-optimized and optimized geometries, respectively, obtained facing the geometry in Panel b1 of Figure S4 to the primary rim of the second βCD (colored by atoms; color code as in Figure S1 in Panel a, in light blue in the other Panels). Panels **c1** and **c2** show the side view and top view of the final optimized geometries after an MD run lasting 5 ns of βCD and quercetin in a 1:1 stoichiometry starting from the geometries in Panel **a** with two βCDs facing their primary rims………….S14

Figure S10. Panels **a** and **b** show the potential energy, van der Waals contribution and Coulomb energy calculated during an MD run lasting 5 ns starting from the optimized geometry in Panel **b** of **Figure S9**. Panels **c** and **d** display the distance between the c.o.m.s of two βCDs and the distribution of this distance calculated during the MD run, respectively. Panels **e** and **f** display the distance between the c.o.m.s of two βCDs and the c.o.m.s of quercetin molecules, respectively, together with the distribution of these distances calculated during the MD run………………………………………………………………………………S15

Figure S11. Panels **a** and **b** show the initial *non-optimized* and optimized geometries, respectively, obtained facing the geometry in Panel **b1** of Figure **S4** to the primary rim of the second βCD (colored by atoms, color code as in Figure **S1** in Panel **a**, in light blue in the other Panels). Panels **c1** and **c2** show the side view and top view of the final optimized geometries after an MD run lasting 5 ns of βCD and quercetin in a 1:1 stoichiometry starting from the geometries in Panel **a** with two βCDs facing their primary and secondary rims…………………………………………………………………………………………………......S16

Figure S12. Panels **a** and **b** show the potential energy, van der Waals contribution and Coulomb energy calculated during an MD run lasting 5 ns starting from the optimized geometry in Panel **b** of **Figure S11**. Panels **c** and **d** display the distance between the c.o.m.s of two βCDs and the distribution of this distance calculated during the MD run, respectively. Panels **e** and **f** display the distance between the c.o.m.s of two βCDs and the c.o.m.s of quercetin molecules, respectively, together with the distribution of these distances calculated during the MD run……………………………………………………………………………….…S17

Figure S13. Panels **a** and **b** show the initial *non-optimized* and optimized geometries, respectively, obtained facing the geometry in Panel **b2** of Figure **S4** to the secondary rim of the second βCD (colored by atoms, color code as in Figure **S1** in Panel **a**, in light blue in the other Panels). Panels **c1** and **c2** show the side view and top view of final optimized geometries after an MD run lasting 5 ns of βCD and quercetin in a 1:1 stoichiometry starting from the geometries in Panel **a** with two βCDs facing their secondary rims……….S18

Figure S14. Panels **a** and **b** show the potential energy, van der Waals contribution and Coulomb energy calculated during an MD run lasting 5 ns starting from the optimized geometry in Panel **b** of **Figure S13**. Panels **c** and **d** display the distance between the c.o.m.s of two βCDs and the distribution of this distance calculated during the MD run, respectively. Panels **e** and **f** display the distance between the c.o.m.s of two βCDs and the c.o.m.s of quercetin molecules, respectively, together with the distribution of these distances calculated during the MD run…………………………………………………………………….....................S19

Intermolecular Interactions Between *B*-DNA and βCD/Q Host–guest Complexes

Intermolecular Interactions Between *B*-DNA and βCD/Q Host–guest Complexes in a 4:4 stoichiometry

Figure S15. Initial *non-optimized* geometry with four different βCD/Q inclusion complexes in a 1:1 stoichiometry as shown in Figure **S4**, in a random arrangement in the simulation cell (see edge shown in white lines) containing the DNA in the center. Color code: carbon atoms are in gray; oxygen in red; nitrogen in blue; and hydrogen in white. For clarity, all βCD atoms are in green. All atoms are in CPK representation……………………………………………………………………………………………...…S20

Figure S16. Panel **a** shows the final optimized geometry obtained after an MD run lasting 50 ns starting from the initial geometry shown in Figure S15, without the edges of simulation box for clarity.
Panel **b** (top) shows a detail of the only four βCD/Q inclusion complexes as in the final adsorption geometry in a major groove of the DNA. Color code for Q molecules in *ball and stick* representation: carbon atoms are in gray; oxygen in red; and hydrogen in white. For clarity, all βCD atoms in stick representation are in green and all DNA atoms in blue.
Panel **b** (below) shows all four quercetin molecules colored by atoms and, for all four βCDs colored in light blue for clarity, the best-fit planes with the arrow pointing towards the secondary rim of each βCD and for the three βCDs facing the secondary rims. It also shows the three centers of mass of the three CDs defining a nearly equilateral triangle, with sides equal to 10.6 Å, 12.0 Å, 9.50 Å. All inclusion complexes are in a βCD/Q 1:1 stoichiometry. Apart from the three βCDs that face the secondary rims, two CDs face the secondary rim and one primary rim, with a π-π interaction between two B aromatic rings of two quercetin molecules forming, respectively, two host–guest complexes of the P1 and P2 inclusion complex types, as detailed in Table S3…………………………………………………………………………...………S21

Figure S17. Panels **a** and **b** show the potential energy, van der Waals contribution and Coulomb energy calculated during an MD run lasting 50 ns starting from the optimized geometry shown in Panel **a** of **Figure S14**. Panels **c** and **d** display the relative concentration of all atoms in the simulation box calculated during the MD run for all frames, periodically saved every 20 ps from 0 to 20 ns in Panel **c**, and from 20 to 50 ns in Panel **d**, respectively……………………………………………...…………………………………….S22

Intermolecular Interactions Between *B*-DNA and βCD/Q Host–guest Complexes in a 8:8stoichiometry

Figure S18. Initial non-optimized geometry with eight different βCD/Q inclusion complexes in a 1:1 stoichiometry as shown in Figure **S4**, in a random arrangement in the simulation cell (see edge shown in white lines) containing the DNA in the center. Color code of DNA and quercetin drug included in host–guest complexes: carbon atoms are in gray; oxygen in red; nitrogen in blue; and hydrogen in white. For clarity, all βCD atoms of the same kind of inclusion complex (Figure S4) are of the same color, yellow or green or light blue or blue. All atoms are in CPK representation…………………………..……………..S23

Figure S19. Panel **a** shows the final optimized geometry obtained after an MD run lasting 50 ns starting from the initial geometry shown in Figure S18, without the edges of simulation box for clarity. Panels **b1** and **b2** show details of the only DNA and 8 quercetin molecules without the βCD atoms as in the final adsorption geometry. Color code for Q molecules: carbon atoms are in gray; oxygen in red; and hydrogen in white. For clarity, all DNA atoms are in blue and, in Panel **a**, all βCD atoms in stick representation are in green. Panel **c** shows all eight quercetin molecules colored by atoms and, for all eight βCDs colored in light blue for clarity, the best-fit planes with the arrow pointing towards the secondary rim of each βCD. For all eight βCDs, the centers of mass are shown together with their distances in a particular alignment which follows the double-stranded DNA architecture. The distances calculated are equal to 9.878 Å, 9.285 Å, 8.663 Å, 15.497 Å, 12.819 Å, 9.273 Å and 10.729 Å…………………………………………………………...…S24

Figure S20. Panels **a** and **b** show the potential energy, van der Waals contribution and Coulomb energy calculated during an MD run lasting 50 ns starting from the optimized geometry shown in Panel **a** of **Figure S18**. Panels **c** and **d** display the relative concentration of all atoms in the simulation box calculated during the MD run for all frames periodically saved every 20 ps from o to 20 ns in Panel **c**, and from 20 to 50 ns in Panel **d**, respectively…………………………………………………………………..……………..S25

Figure S21. Panels **a1**, **b1**, and **c1** illustrate the three different initial *non-optimized* geometries with eight βCD/Q inclusion complexes in a 1:1 stoichiometry as shown in Figure **S4**, in a random arrangement in the simulation cell (see edge shown in white lines) containing the *B*-DNA in the central part. Color code: carbon atoms are in gray; oxygen in red; nitrogen in blue; and hydrogen in white. All atoms are in CPK representation. Panels **a2**, **b2**, and **c2** show the three final optimized geometries obtained after MD runs lasting 50 ns and the energy minimizations. All DNA atoms are in blue, the β-cyclodextrins are in green. Color code for quercetin drug molecules: carbon atoms are in gray; oxygen in red; nitrogen in blue; and hydrogen in white. Only quercetin molecules are in CPK representation. Panels **a3**, **b3**, and **c3** report the details of the arrangement of drug molecules only…….……………………………..…………………….S26

Intermolecular Interactions Between *B*-DNA and βCD/Q Host–guest Complexes in a 12:12 stoichiometry

Figure S22. Initial non-optimized geometry with twelve different βCD/Q inclusion complexes in a 1:1 stoichiometry as shown in Figure **S4**, in a random arrangement in the simulation cell (see edge shown in white lines) containing the DNA in the center. Color code of DNA and quercetin drug included in host–guest complexes: carbon atoms are in gray; oxygen in red; nitrogen in blue; and hydrogen in white. For clarity, all βCD atoms of the same kind of inclusion complex (Figure **S4**) are of the same color, yellow or green or light blue or blue. All atoms are in CPK representation……………………………..…………….S27

Figure S23. Panel **a** shows the final optimized geometry obtained after an MD run lasting 50 ns starting from the initial geometry shown in Figure S21, without the edges of the simulation box for clarity. Panel **b**

shows a detail of the only DNA and 12 quercetin molecules as in the final adsorption geometry without the βCD atoms. Color code for Q molecules in CPK representation: carbon atoms are in gray; oxygen in red; and hydrogen in white. For clarity, all βCD atoms in stick representation are in green and all DNA atoms in blue. Panel **c** shows all twelve quercetin molecules colored by atoms and, for all twelve βCDs colored in light blue for clarity, the best-fit planes with the arrow pointing towards the secondary rim of each βCD. Note the alignment along the DNA backbone with the formation of some βCD/Q dimers in 2:2 stoichiometry, as well as an aggregation of inclusion complexes at the lower end of the DNA……………S28

Figure S24 ~~**S23**~~. Panels **a** and **b** show the potential energy, van der Waals contribution and Coulomb energy calculated during an MD run lasting 50 ns starting from the optimized geometry shown in Panel **a** of **Figure S22**. Panels **c** and **d** display the relative concentration of all atoms in the simulation box calculated during the MD run for all frames periodically saved every 20 ps from 0 to 20 ns in Panel **c**, and from 20 to 50 ns in Panel **d**, respectively…………………………………………………………………..……………………...S29

Figure S25. Panels **a1**, **b1**, and **c1** illustrate the three different initial *non-optimized* geometries with twelve βCD/Q inclusion complexes in a 1:1 stoichiometry as shown in Figure **S4**, in a random arrangement in the simulation cell (see edge shown in white lines) containing the *B*-DNA in the central part. Color code: carbon atoms are in gray; oxygen in red; nitrogen in blue; and hydrogen in white. All atoms are in CPK representation. Panels **a2**, **b2**, and **c2** show the three final optimized geometries obtained after MD runs lasting 50 ns and the energy minimizations. All DNA atoms are in blue, the β-cyclodextrins are in green. Color code for quercetin drug molecules: carbon atoms are in gray; oxygen in red; nitrogen in blue; and hydrogen in white. Only quercetin molecules are in CPK representation. Panels **a3**, **b3**, and **c3** report the details of the arrangement of drug molecules only…...………...S30

Intermolecular Interactions Between *B*-DNA and βCD/Q Host–guest Complexes in a 16:16 stoichiometry

Figure S26. Initial non-optimized geometry with sixteen different βCD/Q inclusion complexes in a 1:1 stoichiometry as shown in Figure **S4**, in a random arrangement in the simulation cell (see edge shown in white lines) containing the DNA in the center. Color code of DNA and quercetin drug included in host–guest complexes: carbon atoms are in gray; oxygen in red; nitrogen in blue; and hydrogen in white. For clarity, all βCD atoms of the same kind of inclusion complex (Figure **S4**) are of the same color, yellow or green or light blue or blue. All atoms are in CPK representation………………………………..……….S31

Figure S27. Panel **a** shows the final optimized geometry obtained after an MD run lasting 50 ns starting from the initial geometry shown in Figure S24, without the edges of simulation box for clarity. Panel **b** shows a detail of the only DNA and 16 quercetin molecules as in the final adsorption geometry without the βCD atoms. Color code for Q molecules in CPK representation: carbon atoms are in gray; oxygen in red; and hydrogen in white. For clarity, all βCD atoms in stick representation are in green, and all DNA atoms in blue. Panel **c** shows all sixteen quercetin molecules colored by atoms and, for all sixteen βCDs colored in light blue for clarity, the best-fit planes with the arrow pointing towards the secondary rim of each βCD. Note the alignment along the DNA backbone with the formation of some βCD/Q dimers in 2:2 stoichiometry and also an aggregation of inclusion complexes at the ends of the DNA…………………..…S32

Figure S28. Panels **a** and **b** show the potential energy, van der Waals contribution and Coulomb energy calculated during an MD run lasting 50 ns starting from the optimized geometry shown in Panel **a** of **Figure S26**. Panels **c** and **d** display the relative concentration of all atoms in the simulation box calculated during

the MD run for all frames periodically saved every 20 ps from 0 to 20 ns in Panel **c**, and from 20 to 50 ns in Panel **d**, respectively…………………………………………………………………..………………..….S33

Intermolecular Interactions Between *B*-DNA and one Quercetin Molecule

Figure S29. Side view of the optimized geometries obtained after MD runs lasting 10 ns related to the adsorption of quercetin on the DNA surface starting from four different geometries with drug molecules parallel or perpendicular near to a minor or major groove. The animation of the four MD runs is shown in file.avi shown below. The DNA is colored in green and quercetin molecules by atoms (carbon atoms in gray, oxygen atoms in red and hydrogen atoms in white) are in CPK representation. Panels **e** and **f** show the distance of the quercetin c.o.m. and the line that define the axis along which the DNA fragment unfolds calculated during the four MD runs performed……………………………………….………………..S32

Intermolecular Interactions Between *B*-DNA and Quercetin Molecules at Different Concentrations

Figure S30. Potential energy and van der Waals contribution calculated during MD runs lasting 10 ns starting from the optimized geometry considering DNA in the central part of the simulation cell, and four, eight, twelve, and sixteen quercetin molecules in Panels **a**, **b**, **c** and **d**, respectively………….…………S35

Intermolecular Interactions Between *B*-DNA and one βCD molecule

Figure S31. Optimized geometries related to the interaction between double-stranded *B*-DNA and one in βCD obtained after six different MD runs lasting 10 ns starting from six different initial non-optimized geometries with the βCD primary rim parallel to a minor or major DNA groove, the βCD secondary rim parallel to a minor or major DNA groove, and the βCD best-fit plane perpendicular to the DNA axis near to a minor or major groove……………………………………………………………………………..S37

Table S4. Interaction energy (kJ/mol), intramolecular H-bonds in βCD and intermolecular H-bonds B-DNA/βCD in the **final optimized geometries** after a 10 ns MD run, as shown in Figure **S31** ……………S36

Intermolecular Interactions Between *B*-DNA and βCDs at Different Concentrations

Figure S32. Potential energy and van der Waals contribution calculated during MD runs lasting 20 ns starting from the optimized geometry obtained considering DNA in the central part of the simulation cell, and four, eight, twelve, and sixteen βCDs in Panels **a**, **b**, **c**, and **d**, respectively……………………….……S37

Intermolecular Interactions Between βCDs at Different Concentrations

Figure S33. Initial non-optimized geometries related to four, eight, twelve, and sixteen βCDs in the simulation cell. Color code: carbon atoms are in gray; oxygen in red; and hydrogen in white. All atoms are in CPK representation……………………………………………………………………………..…S38

Figure S34. Potential energy and van der Waals contribution calculated during MD runs lasting 20 ns starting from the non-optimized geometries shown in Figure **S33** related to four, eight, twelve, and sixteen βCDs in Panels **a**, **b**, **c**, and **d**, respectively…………………………………………...…………………..S38

Figure S35. Solvent-accessible surface area (SASA) colored by atoms of the final optimized geometries related to four, eight, twelve, and sixteen βCDs in the simulation cell after MD runs lasting 20 ns. The color code is the same as in Figure **S33**…………………………………………………………..………………..….S39

Intermolecular Interactions Between βCDs and Quercetin Molecules at Different Concentrations

Figure S36. Initial non-optimized geometries related to four, eight, twelve, and sixteen βCDs and quercetin molecules in the simulation cell. Color code: all βCD atoms are in light blue, quercetin carbon atoms are in gray; oxygen in red; and hydrogen in white. All atoms are in CPK representation………………………….S40

Figure S37. Potential energy and van der Waals contribution calculated during MD runs lasting 20 ns starting from the non-optimized geometries shown in Figure **S33** related to four, eight, twelve, and sixteen βCDs in Panels **a**, **b**, **c**, and **d**, respectively……………………………………………………………………….S40

Figure S38. Detail of some inclusion complexes in the final optimized geometries related to twelve (Panel **a**) and sixteen (Panel **b**) βCDs and quercetin molecules in the simulation cell after MD runs lasting 20 ns. The color code for quercetin molecules is the same as in Figure **S36** ~~**S34**~~. The βCDs involved in βCD/Q 1:1 stoichiometry are colored in light blue, βCDs involved in βCD/Q in 1:2 and 2:1 stoichiometry are colored in yellow and in green, respectively, and, finally, the βCDs involved in βCD/Q 2:2 stoichiometry in Panel a are colored in blue. The βCDs are in stick representation, encapsulated quercetin molecules in ball and stick, Q molecules not included in stick representation. The hydrogen atoms are omitted for clarity……..S41

Figure S39. SASA colored by atoms of the final optimized geometries related to four, eight, twelve, and sixteen βCDs and quercetin molecules in the simulation cell after MD runs lasting 20 ns. The color code is the same as in Figure **S36** …………………………………………………………………………….....S42

Figure S40. Encapsulated quercetin molecules that interact with each other in the hydrophobic βCD cavities in the final optimized geometries shown in Figure **S39** related to four, eight, twelve, and sixteen βCDs and quercetin molecules in the simulation cell after MD runs lasting 20 ns, in this figure without the representation of βCDs. The color code is the same as in Figure **2**…………………………………….....S43

Concentration profile……………………………………………………………………………………S44

Structure of βCD and Quercetin

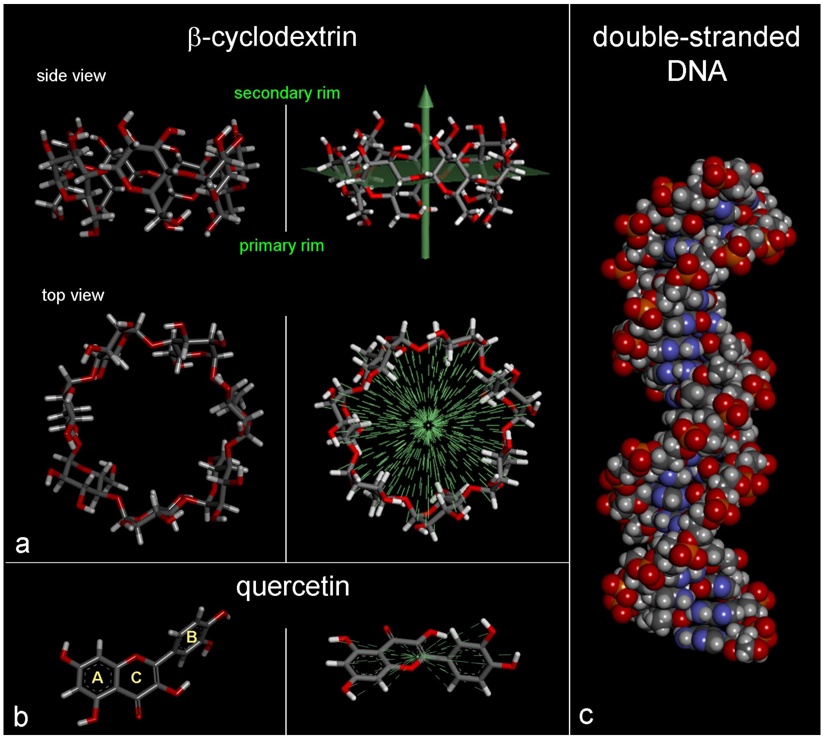

Figure S1. Panel **a** illustrates the side view and top view of the βCD optimized geometry, with indication of the primary and secondary rims. On the left, the plane of best fit and the arrow pointing towards the secondary rim; below, the βCD c.o.m.

Panel **b** displays the optimized geometry of quercetin, with indication of the A, B and C rings (discussed later in the article) and the structure with its c.o.m..

Panel **c** shows the double-stranded DNA structure in a CPK representation. βCD and the drug molecule are represented by sticks. Color code: carbon atoms are in gray; oxygen in red; nitrogen in blue; and hydrogen in white.

Table S1. Intramolecular H-bonds in the optimized β-cyclodextrin and quercetin molecule.

	Intramolecular H-bonds
βCD	29
Quercetin	1

Inclusion Complexes of βCD/Q in a 1:1 Stoichiometry

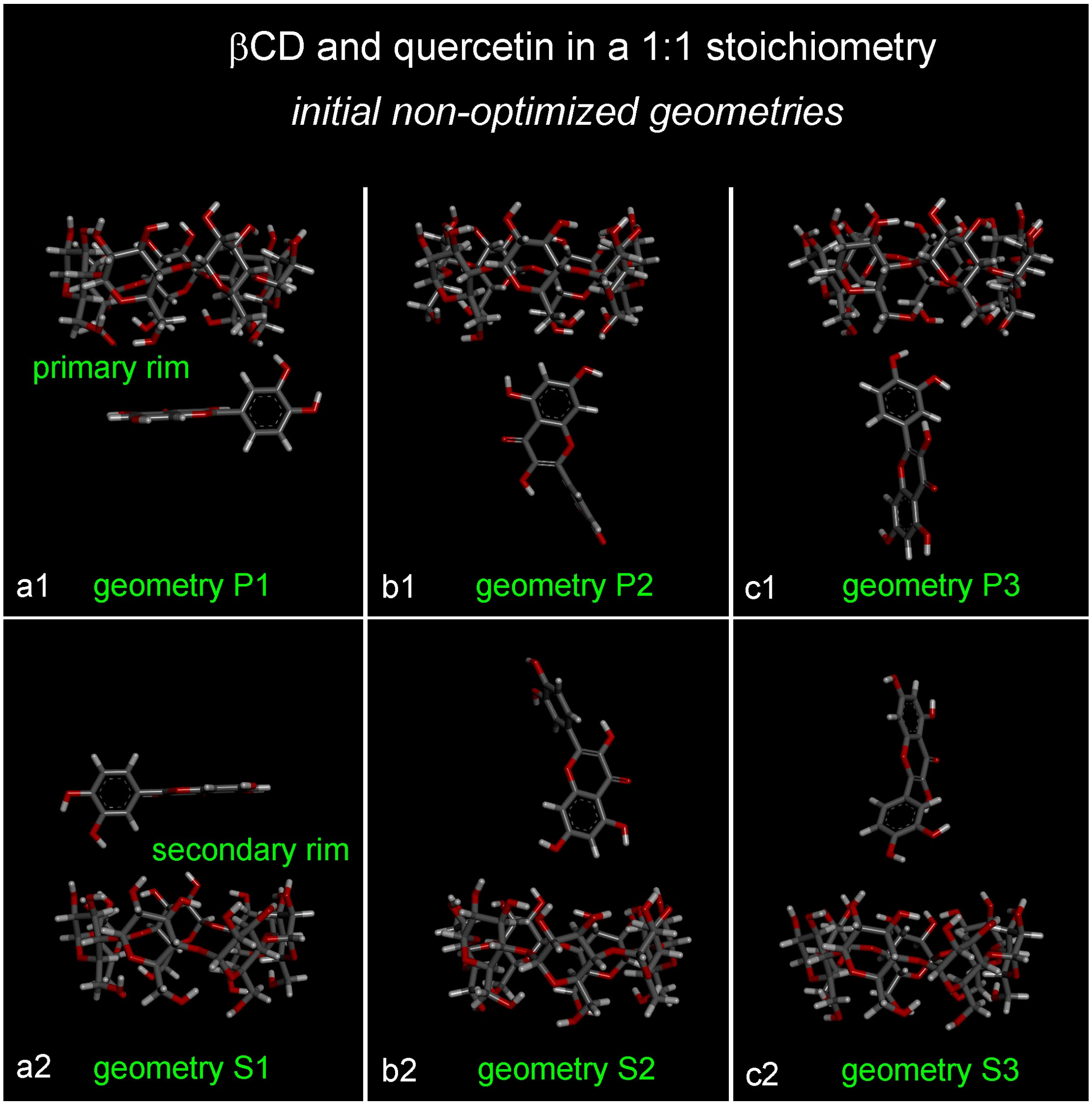

Figure S2. Side view of the initial *non-optimized* geometries of β-cyclodextrin and quercetin in a 1:1 stoichiometry with the drug molecule initially parallel or perpendicular to the βCD primary rim (Panels **a1**, **b1**, and **c1**, respectively) and to the βCD secondary rim (Panels **a2**, **b2**, and **c2**, respectively), without any inclusion a priori. The color code is the same as in Figure **S1**.

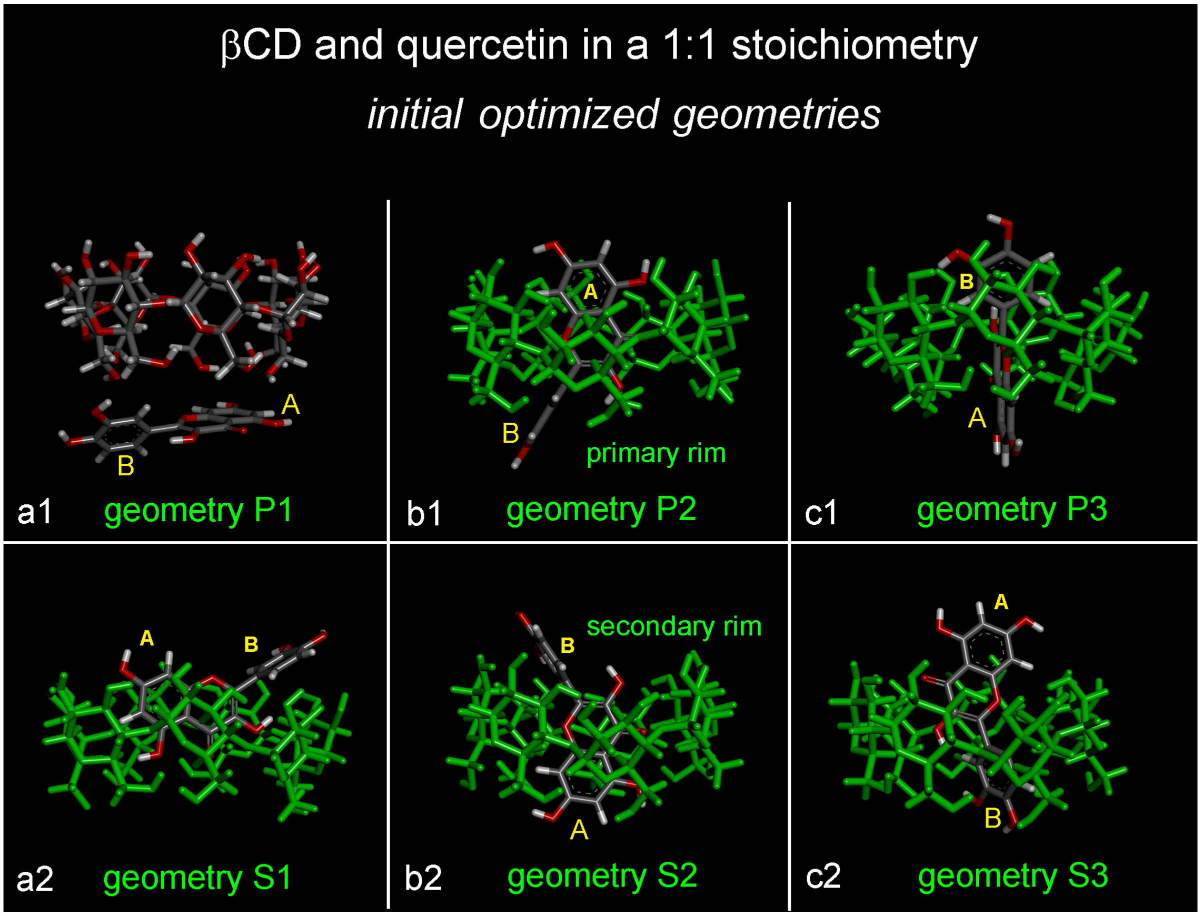

Figure S3. Side view of the initial optimized geometries of β-cyclodextrin and quercetin in a 1:1 stoichiometry with the drug molecule initially parallel or perpendicular to the βCD primary rim (Panels **a1**, **b1**, and **c1**, respectively) and to the βCD secondary rim (Panels **a2**, **b2**, and **c2**, respectively). The color code is the same as in Figure S1. When the drug inclusion process takes place, all atoms of βCD are in green.

Table S2. Interaction energy (kJ/mol), intramolecular H-bonds in βCD and quercetin molecules, and βCD/drug intermolecular H-bonds in the **initial optimized geometries** shown in Figure **S3**.

geometry	E_{int} (kJ/mol)	βCD intramolecular H-bonds	quercetin intramolecular H-bonds	βCD/quercetin intermolecular H-bonds
P1	-52.65	32	1	1
P2	-151.4	25	1	1
P3	-154.4	31	1	0
S1	-147.2	22	1	0
S2	-153.3	27	1	0
S3	-149.4	26	1	1

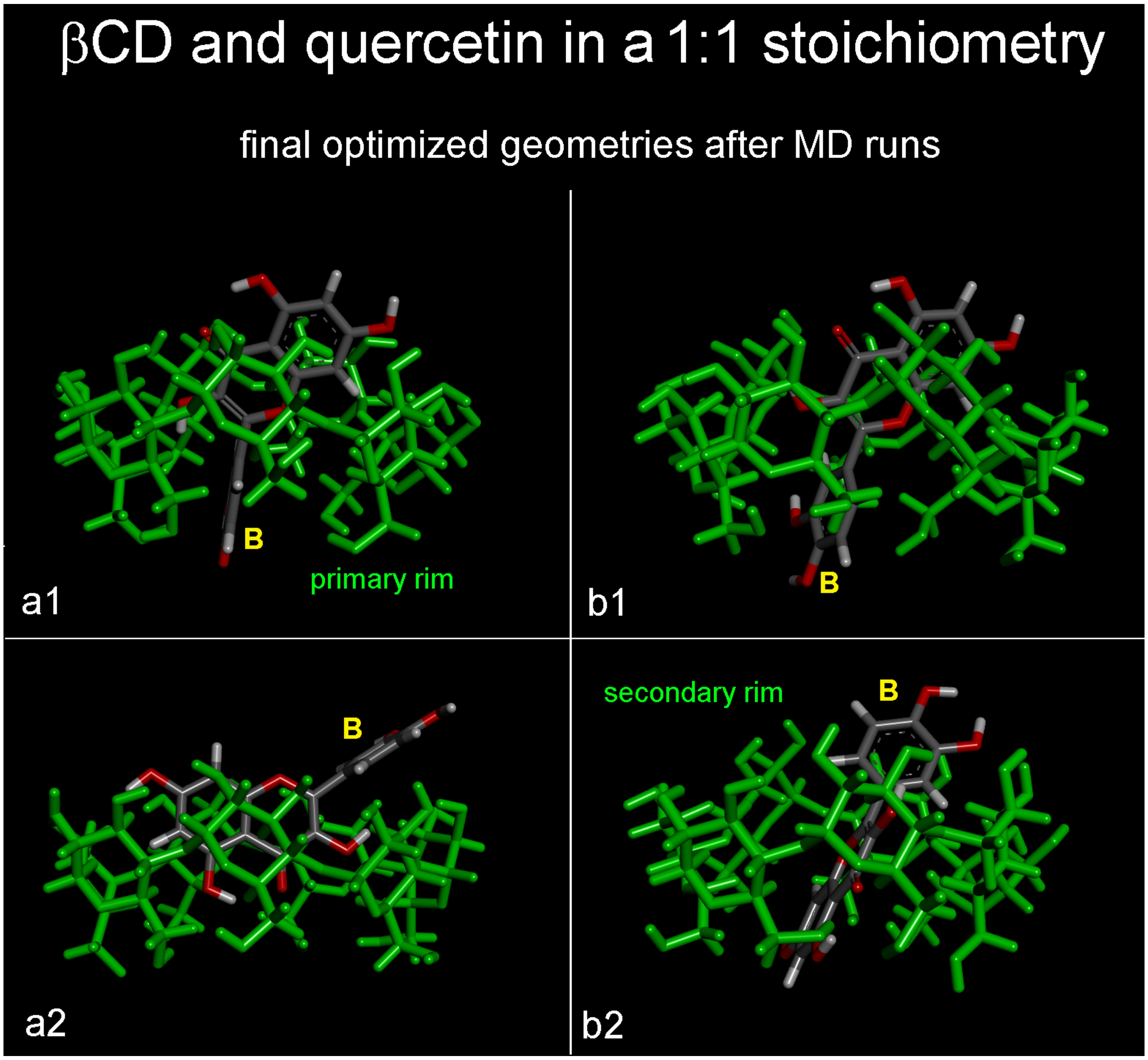

Figure S4. Side view of the final optimized geometries after an MD run lasting 5 ns of β-cyclodextrin and quercetin in a 1:1 stoichiometry starting from the geometries in Panels **a1**, **b1**, **a2** and **b2**, respectively. The color code for the quercetin molecule is the same as in Figure **S1**; all atoms of βCD are in green.

Table S3. Interaction energy (kJ/mol), intramolecular H-bonds in βCD and quercetin molecules, and βCD/drug intermolecular H-bonds in the **final optimized geometries** after a 5 ns MD run as shown in Figure **S4**.

geometry	E_{int} (kJ/mol)	βCD intramolecular H-bonds	quercetin intramolecular H-bonds	βCD/quercetin intermolecular H-bonds
P1	-171.1	10	3	1
P2	-170.6	11	3	1
S1	-149.5	14	1	0
S2	-168.2	12	3	3

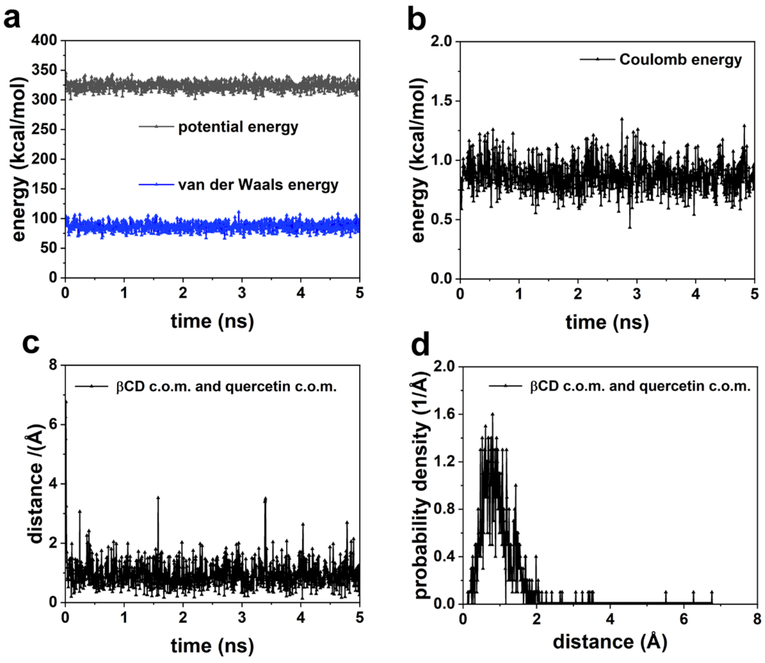

Figure S5. Panels **a** and **b** show the potential energy, van der Waals contribution and Coulomb energy calculated during an MD run lasting 5 ns starting from the geometry in Panel **a1** of **Figure S4 (geometry P1** in **Table S3)**. Panels **c** and **d** display the distance between the βCD and the quercetin center of mass (c.o.m.), and the distribution of these distances calculated during the MD run, respectively.

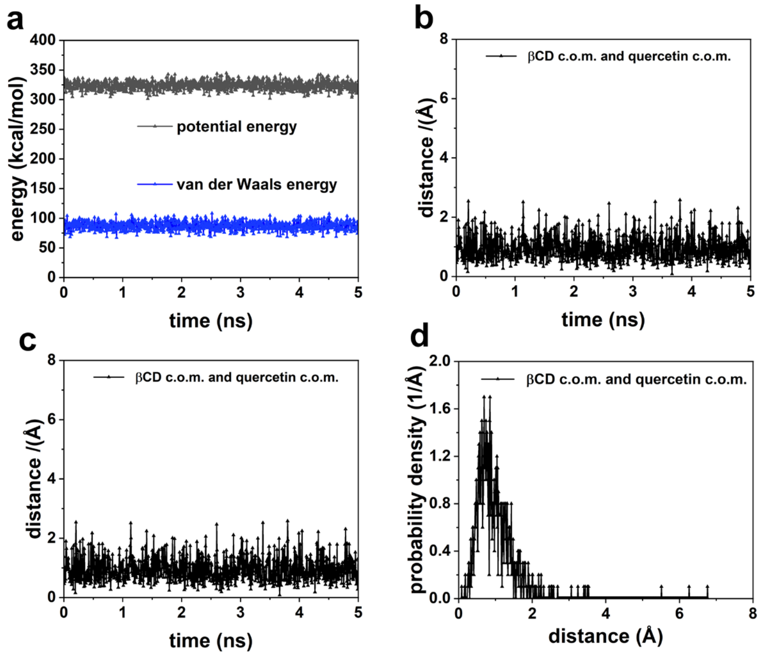

Figure S6. Panels **a** and **b** show the potential energy, van der Waals contribution and Coulomb energy calculated during an MD run lasting 5 ns starting from the geometry in Panel **b1** of **Figure S4 (geometry P2** in **Table S3)**. Panels **c** and **d** display the distance between the βCD and the quercetin center of mass (c.o.m.), and the distribution of these distances calculated during the MD run, respectively.

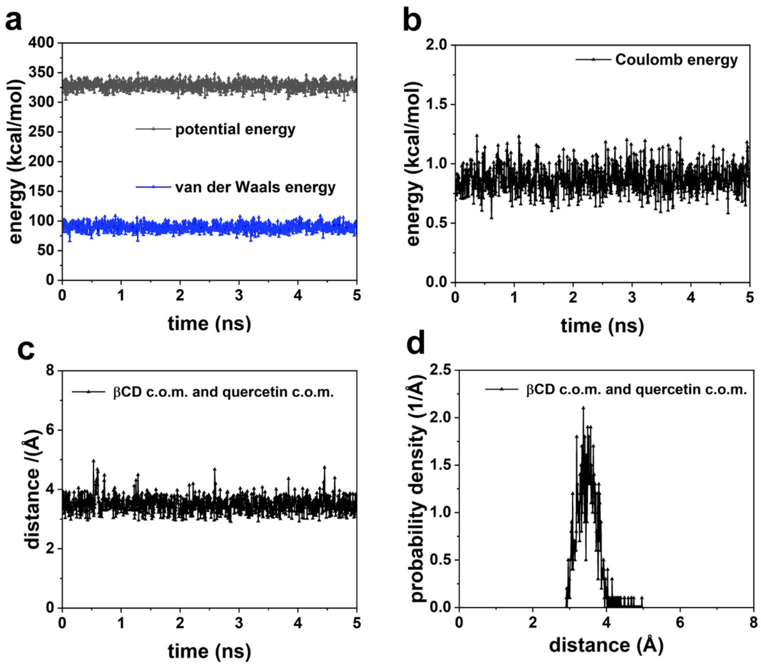

Figure S7. Panels **a** and **b** show the potential energy, van der Waals contribution and Coulomb energy calculated during an MD run lasting 5 ns starting from the geometry in Panel **a2** of **Figure S4 (geometry S1** in **Table S3)**.

Panels **c** and **d** display the distance between the βCD and the quercetin center of mass (c.o.m.), and the distribution of these distances calculated during the MD run, respectively.

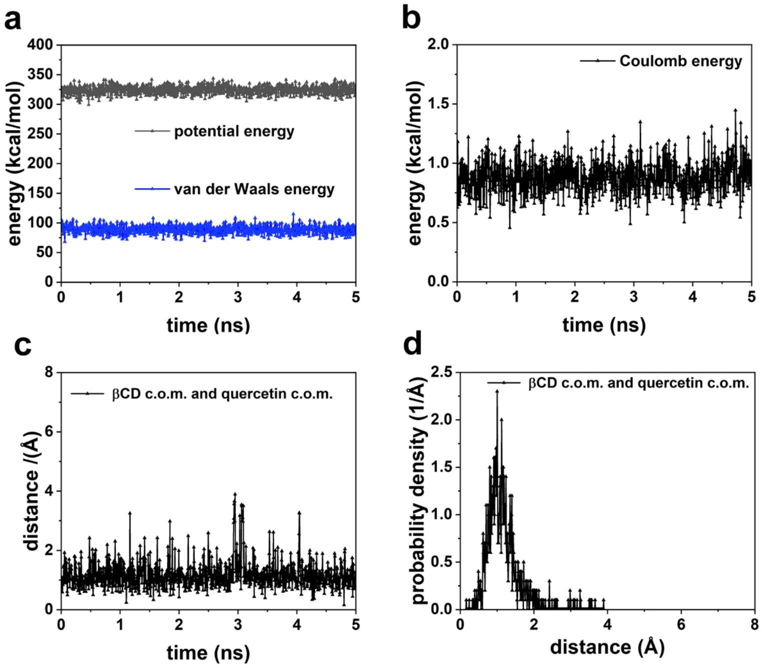

Figure S8. Panels **a** and **b** show the potential energy, van der Waals contribution and Coulomb energy calculated during an MD run lasting 5 ns starting from the geometry in Panel **b2** of **Figure S4 (geometry S2** in **Table S3)**.

Panels **c** and **d** display the distance between the βCD and the quercetin center of mass (c.o.m.), and the distribution of these distances calculated during the MD run, respectively.

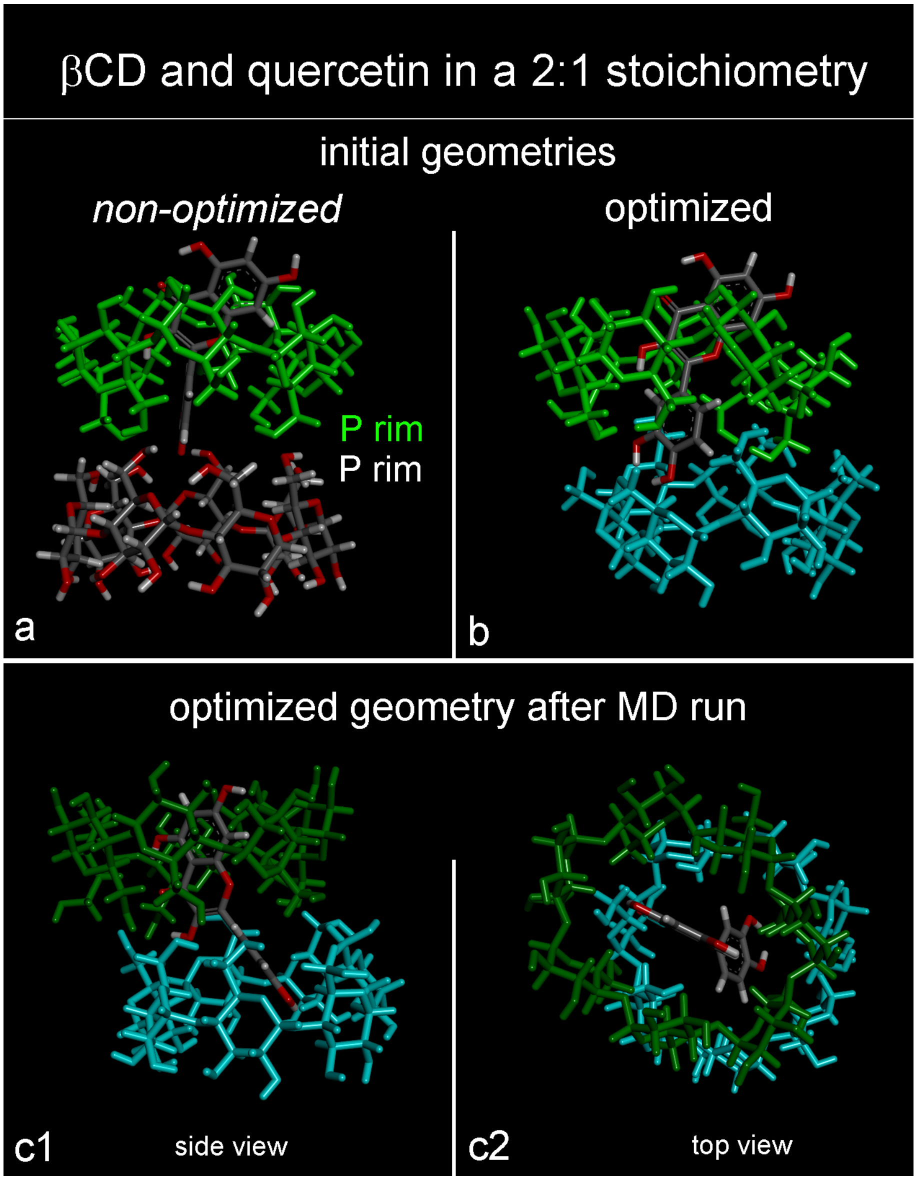

Figure S9. Panels **a** and **b** show the initial non-optimized and optimized geometries, respectively, obtained facing the geometry in Panel b1 of Figure S4 to the primary rim of the second βCD (colored by atoms; color code as in Figure S1 in Panel a, in light blue in the other Panels).
Panels **c1** and **c2** show the side view and top view of the final optimized geometries after an MD run lasting 5 ns of βCD and quercetin in a 1:1 stoichiometry starting from the geometries in Panel **a** with two βCDs facing their primary rims.

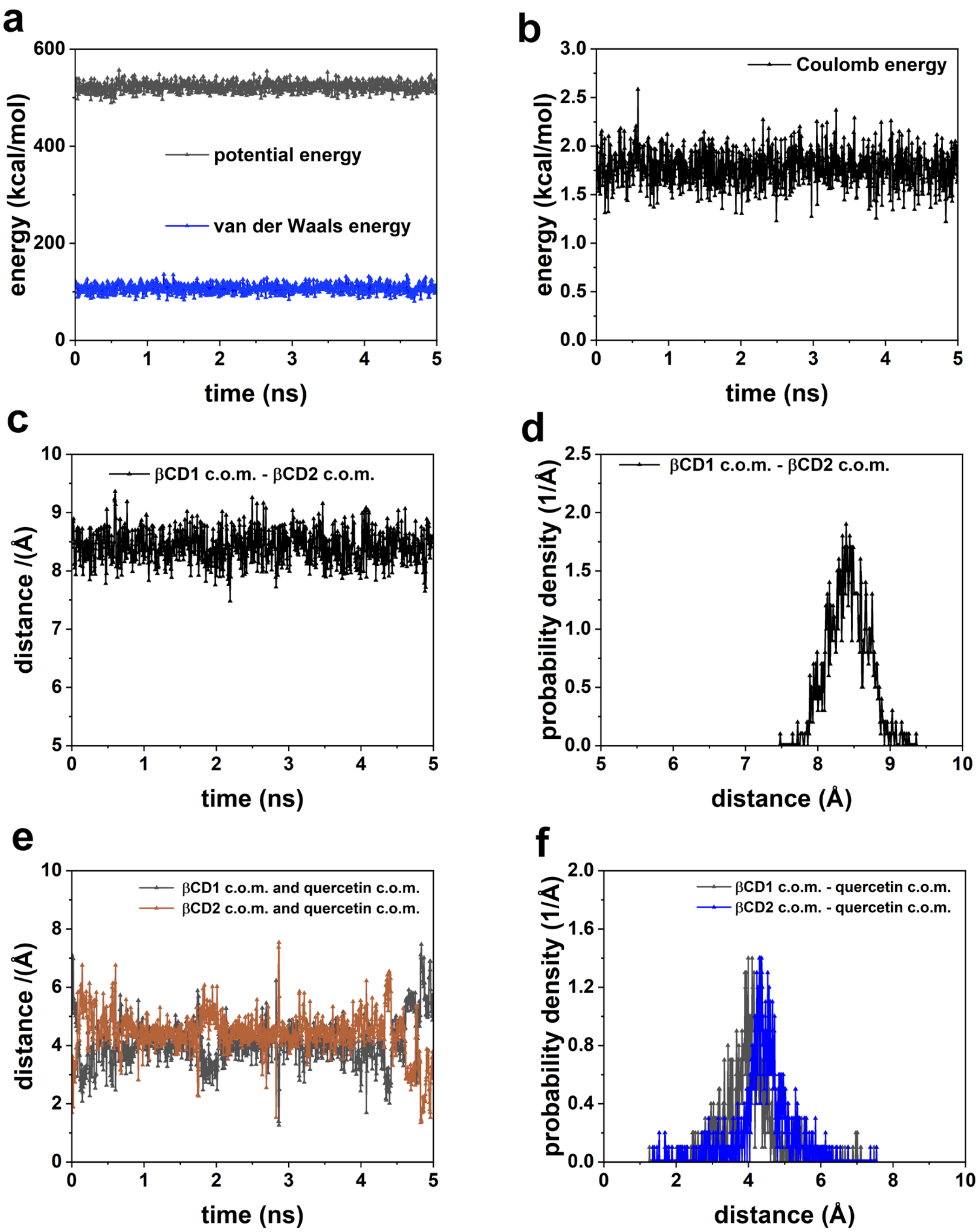

Figure S10. Panels **a** and **b** show the potential energy, van der Waals contribution and Coulomb energy calculated during an MD run lasting 5 ns starting from the optimized geometry in Panel **b** of **Figure S9**. Panels **c** and **d** display the distance between the c.o.m.s of two βCDs and the distribution of this distance calculated during the MD run, respectively.

Panels **e** and **f** display the distance between the c.o.m.s of two βCDs and the c.o.m.s of quercetin molecules, respectively, together with the distribution of these distances calculated during the MD run.

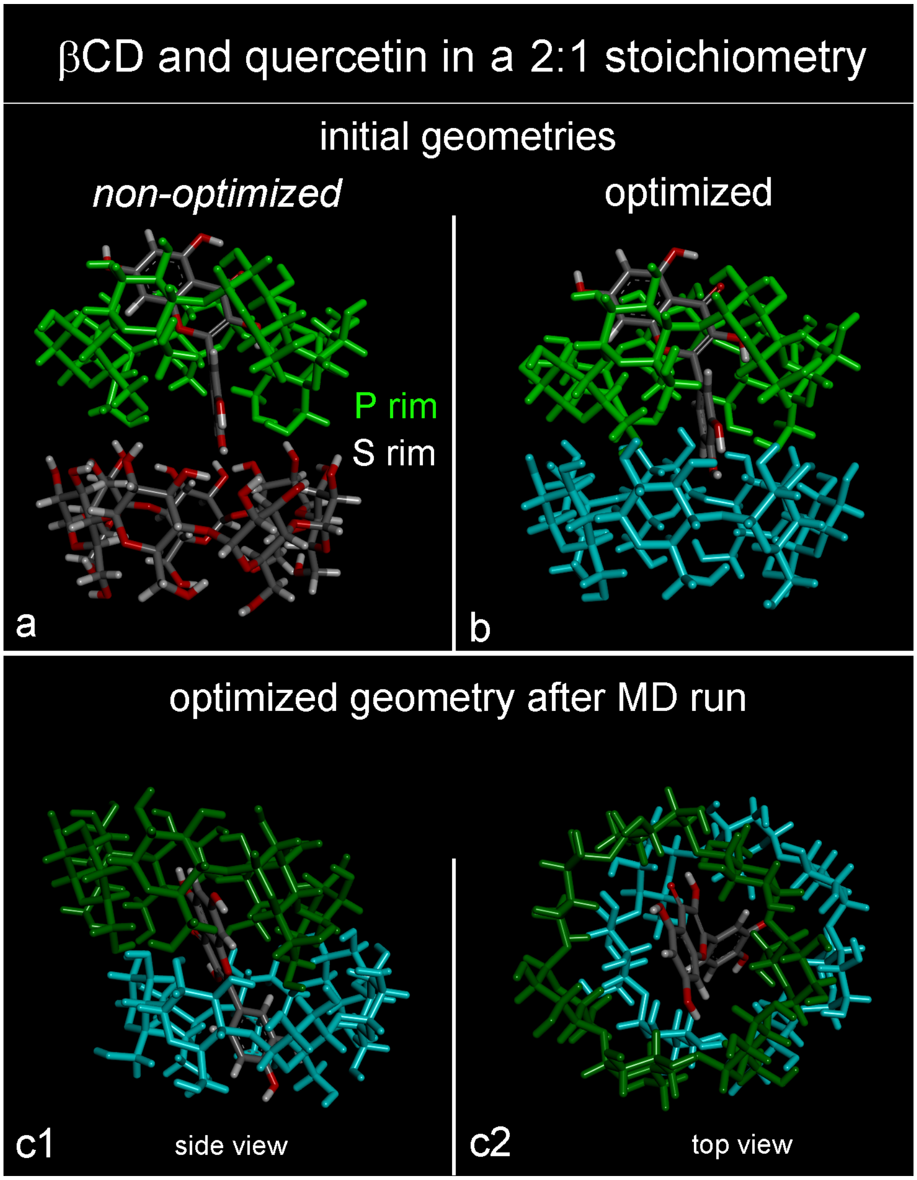

Figure S11. Panels **a** and **b** show the initial *non-optimized* and optimized geometries, respectively, obtained facing the geometry in Panel **b1** of Figure **S4** to the primary rim of the second βCD (colored by atoms, color code as in Figure **S1** in Panel **a**, in light blue in the other Panels).
Panels **c1** and **c2** show the side view and top view of the final optimized geometries after an MD run lasting 5 ns of βCD and quercetin in a 1:1 stoichiometry starting from the geometries in Panel **a** with two βCDs facing their primary and secondary rims.

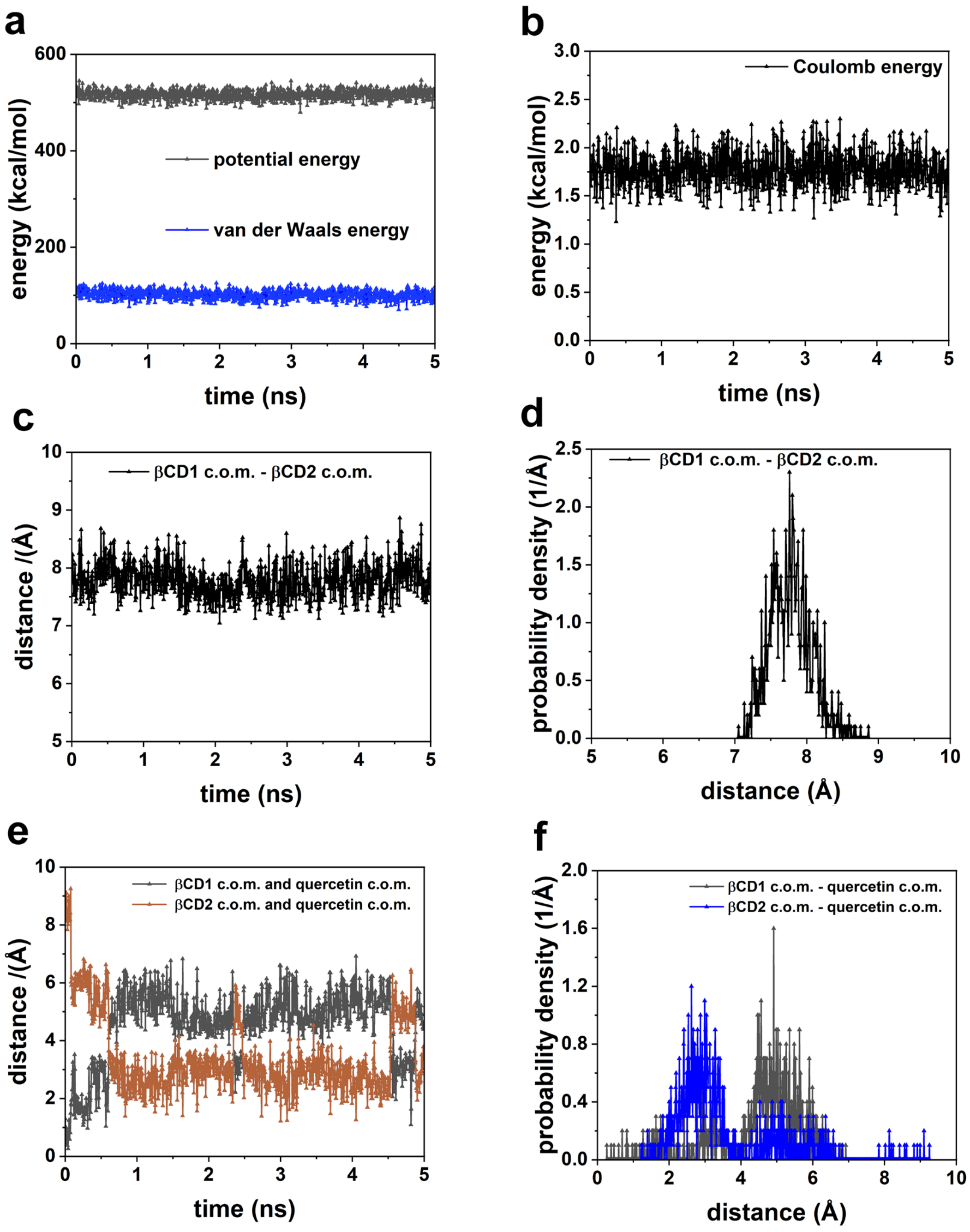

Figure S12. Panels **a** and **b** show the potential energy, van der Waals contribution and Coulomb energy calculated during an MD run lasting 5 ns starting from the optimized geometry in Panel **b** of **Figure S11**. Panels **c** and **d** display the distance between the c.o.m.s of two βCDs and the distribution of this distance calculated during the MD run, respectively. Panels **e** and **f** display the distance between the c.o.m.s of two βCDs and the c.o.m.s of quercetin molecules, respectively, together with the distribution of these distances calculated during the MD run.

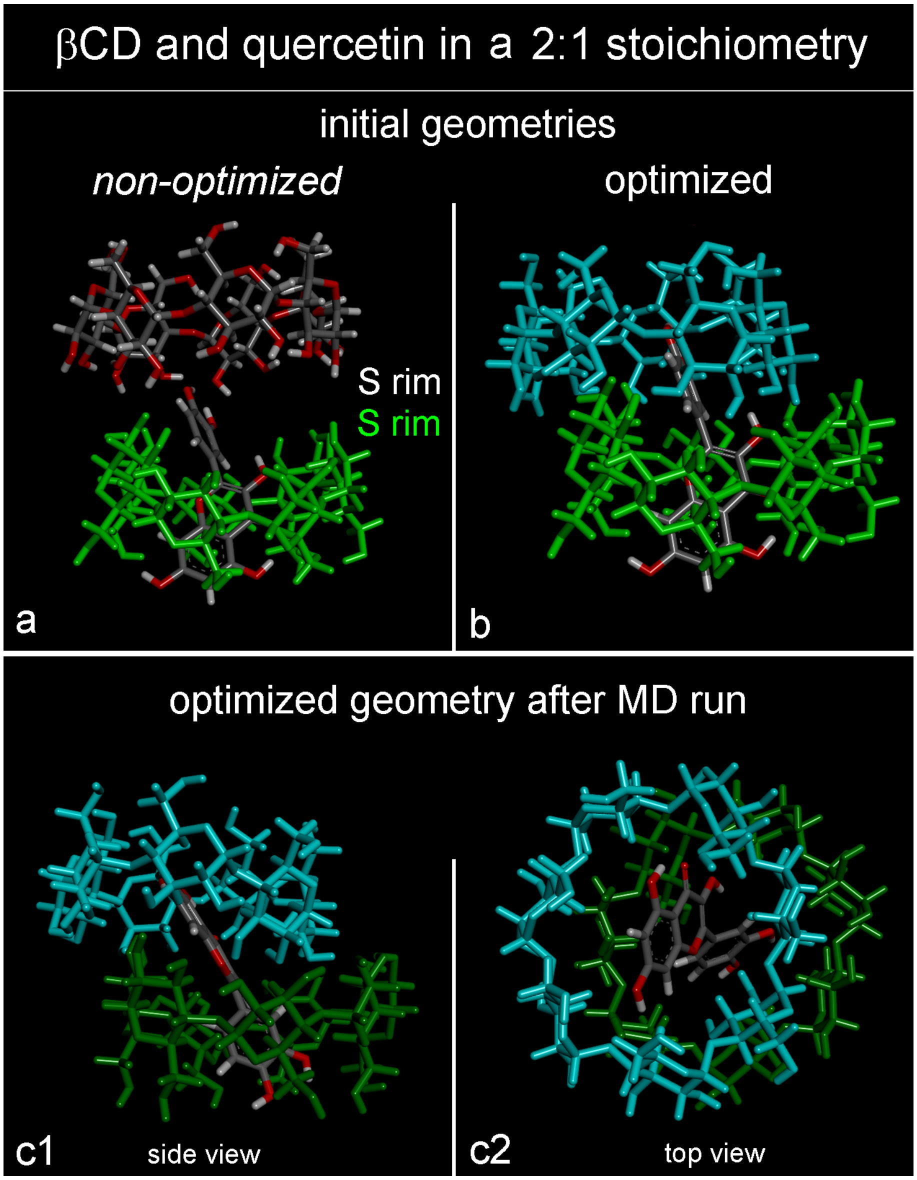

Figure S13. Panels **a** and **b** show the initial *non-optimized* and optimized geometries, respectively, obtained facing the geometry in Panel **b2** of Figure **S4** to the secondary rim of the second βCD (colored by atoms, color code as in Figure **S1** in Panel **a**, in light blue in the other Panels).
Panels **c1** and **c2** show the side view and top view of final optimized geometries after an MD run lasting 5 ns of βCD and quercetin in a 1:1 stoichiometry starting from the geometries in Panel **a** with two βCDs facing their secondary rims.

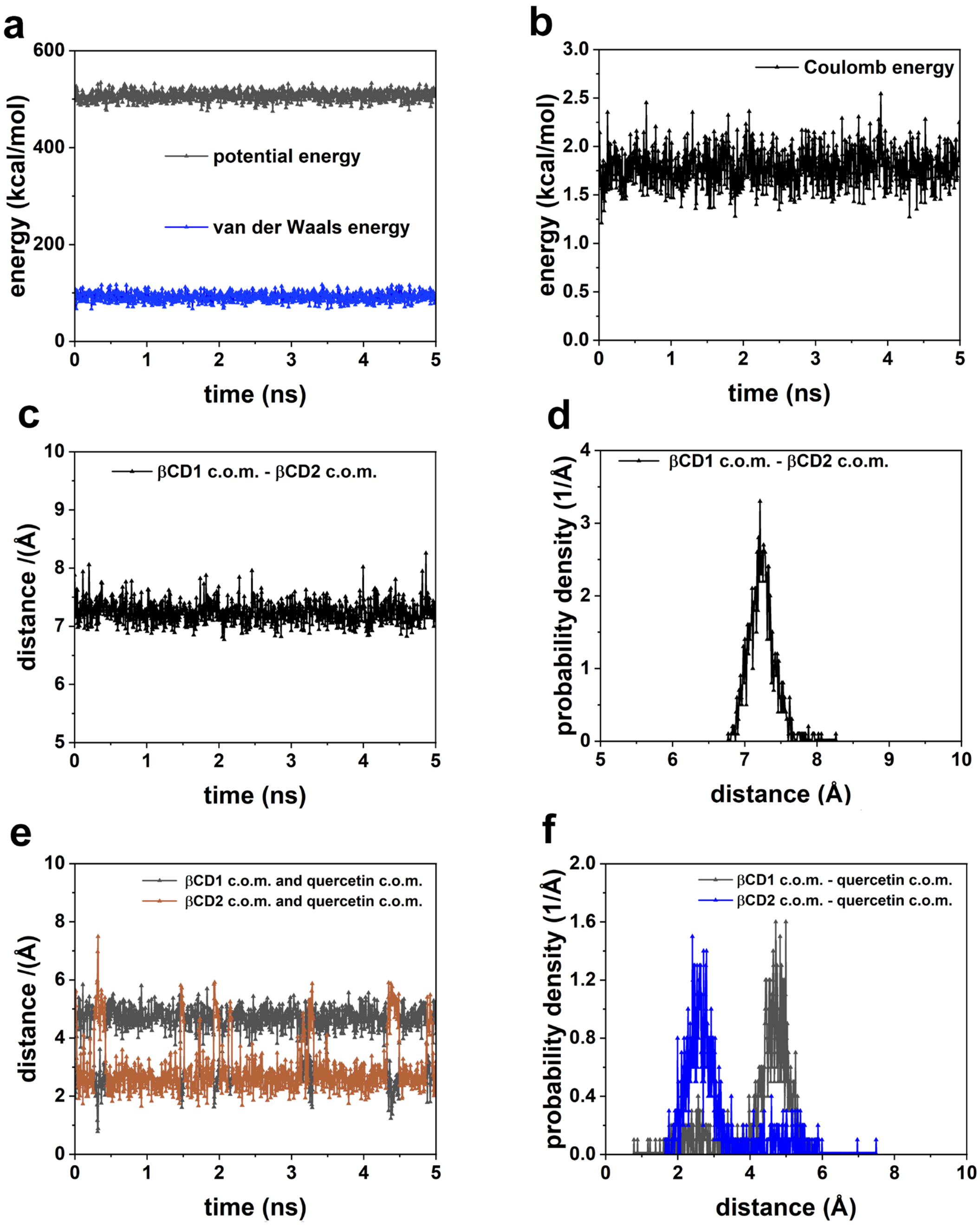

Figure S14. Panels **a** and **b** show the potential energy, van der Waals contribution and Coulomb energy calculated during an MD run lasting 5 ns starting from the optimized geometry in Panel **b** of **Figure S13**. Panels **c** and **d** display the distance between the c.o.m.s of two βCDs and the distribution of this distance calculated during the MD run, respectively. Panels **e** and **f** display the distance between the c.o.m.s of two βCDs and the c.o.m.s of quercetin molecules, respectively, together with the distribution of these distances calculated during the MD run.

Intermolecular Interactions Between *B*-DNA and βCD/Q Host–guest Complexes in a 4:4 Stoichiometry

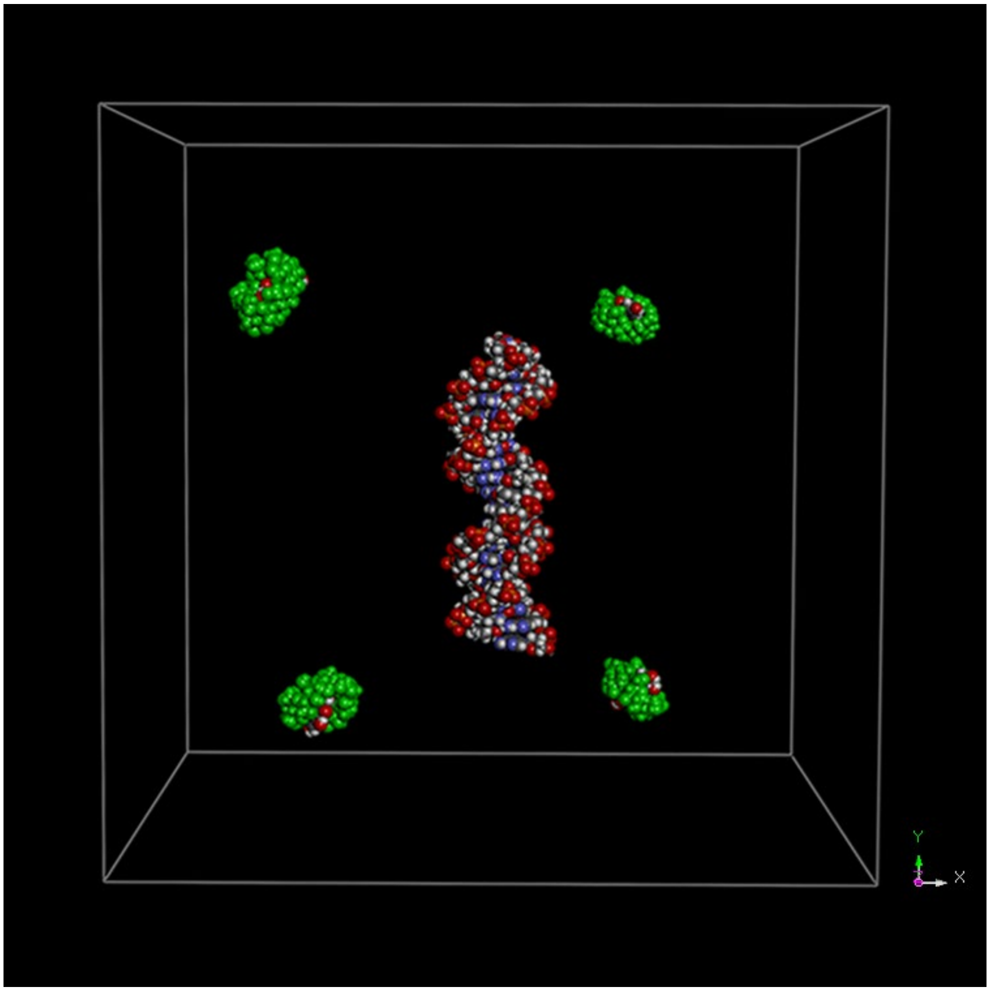

Figure S15. Initial *non-optimized* geometry with four different βCD/Q inclusion complexes in a 1:1 stoichiometry as shown in Figure **S4**, in a random arrangement in the simulation cell (see edge shown in white lines) containing the DNA in the center. Color code: carbon atoms are in gray; oxygen in red; nitrogen in blue; and hydrogen in white. For clarity, all βCD atoms are in green. All atoms are in CPK representation.

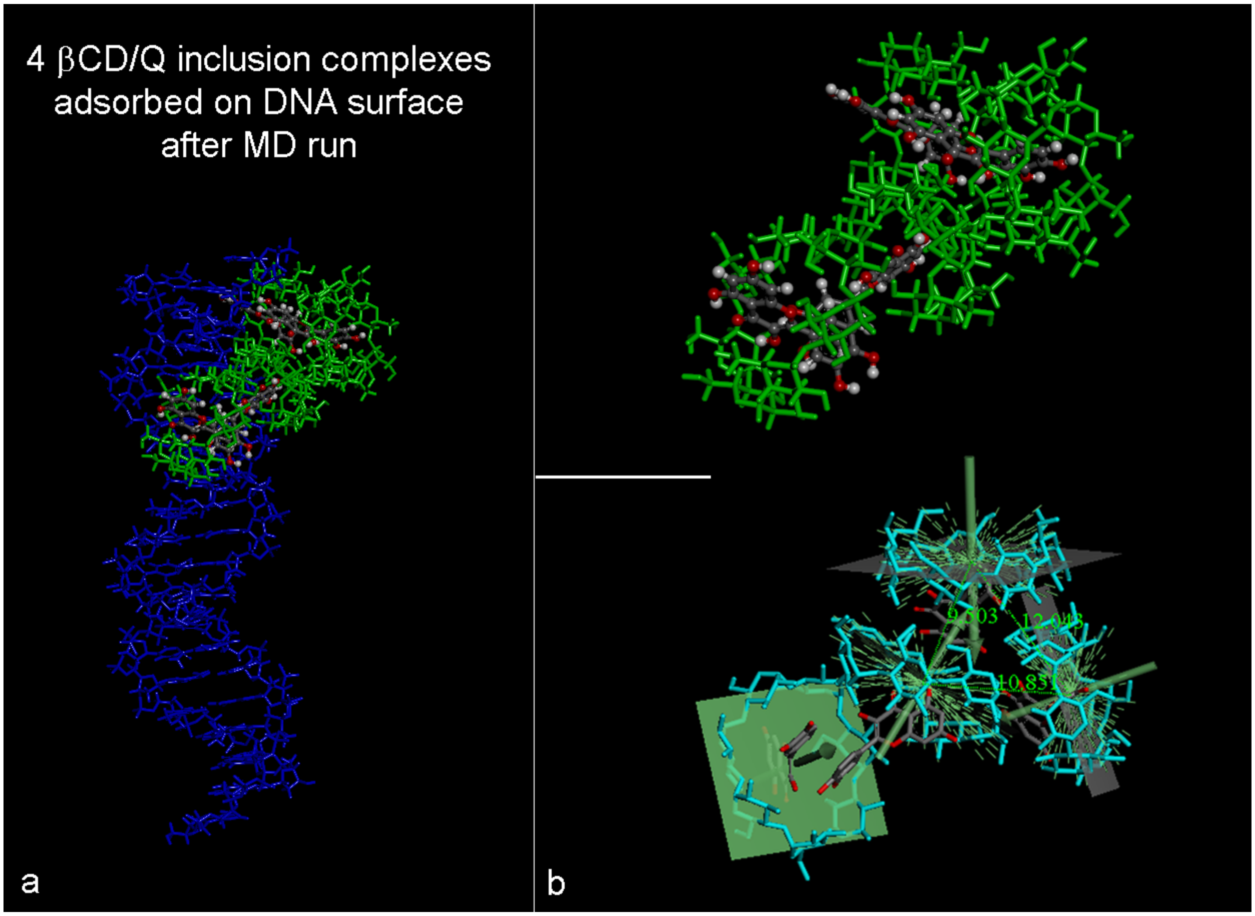

Figure S16. Panel **a** shows the final optimized geometry obtained after an MD run lasting 50 ns starting from the initial geometry shown in Figure S15, without the edges of simulation box for clarity.

Panel **b** (top) shows a detail of the only four βCD/Q inclusion complexes as in the final adsorption geometry in a major groove of the DNA. Color code for Q molecules in *ball and stick* representation: carbon atoms are in gray; oxygen in red; and hydrogen in white. For clarity, all βCD atoms in stick representation are in green and all DNA atoms in blue.

Panel **b** (below) shows all four quercetin molecules colored by atoms and, for all four βCDs colored in light blue for clarity, the best-fit planes with the arrow pointing towards the secondary rim of each βCD and for the three βCDs facing the secondary rims. It also shows the three centers of mass of the three CDs defining a nearly equilateral triangle, with sides equal to 10.6 Å, 12.0 Å, 9.50 Å. All inclusion complexes are in a βCD/Q 1:1 stoichiometry. Apart from the three βCDs that face the secondary rims, two CDs face the secondary rim and one primary rim, with a π-π interaction between two B aromatic rings of two quercetin molecules forming, respectively, two host–guest complexes of the P1 and P2 inclusion complex types, as detailed in Table S3.

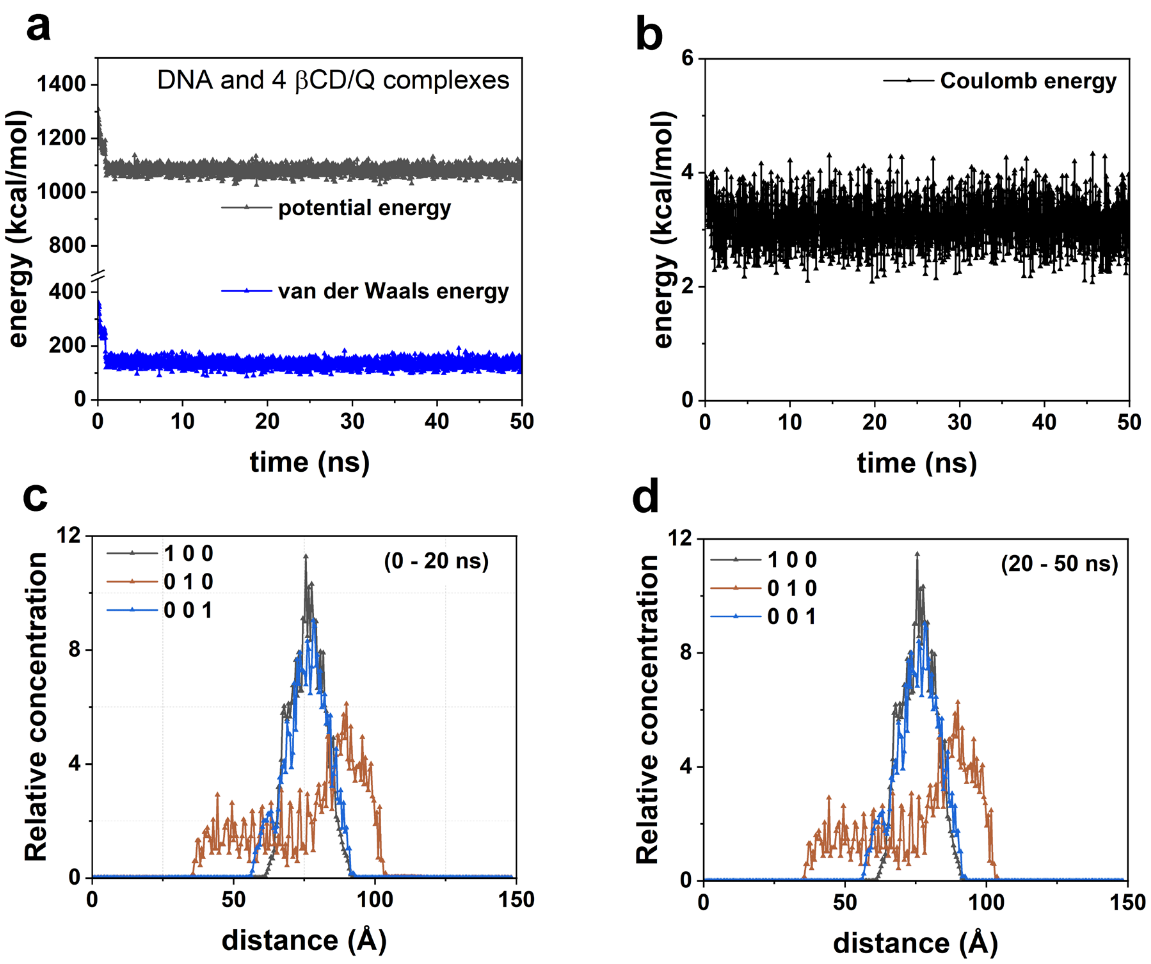

Figure S17. Panels **a** and **b** show the potential energy, van der Waals contribution and Coulomb energy calculated during an MD run lasting 50 ns starting from the optimized geometry shown in Panel **a** of **Figure S14**.

Panels **c** and **d** display the relative concentration of all atoms in the simulation box calculated during the MD run for all frames, periodically saved every 20 ps from 0 to 20 ns in Panel **c**, and from 20 to 50 ns in Panel **d**, respectively.

Intermolecular Interactions Between *B*-DNA and

βCD/Q Host–guest Complexes in a 8:8 Stoichiometry

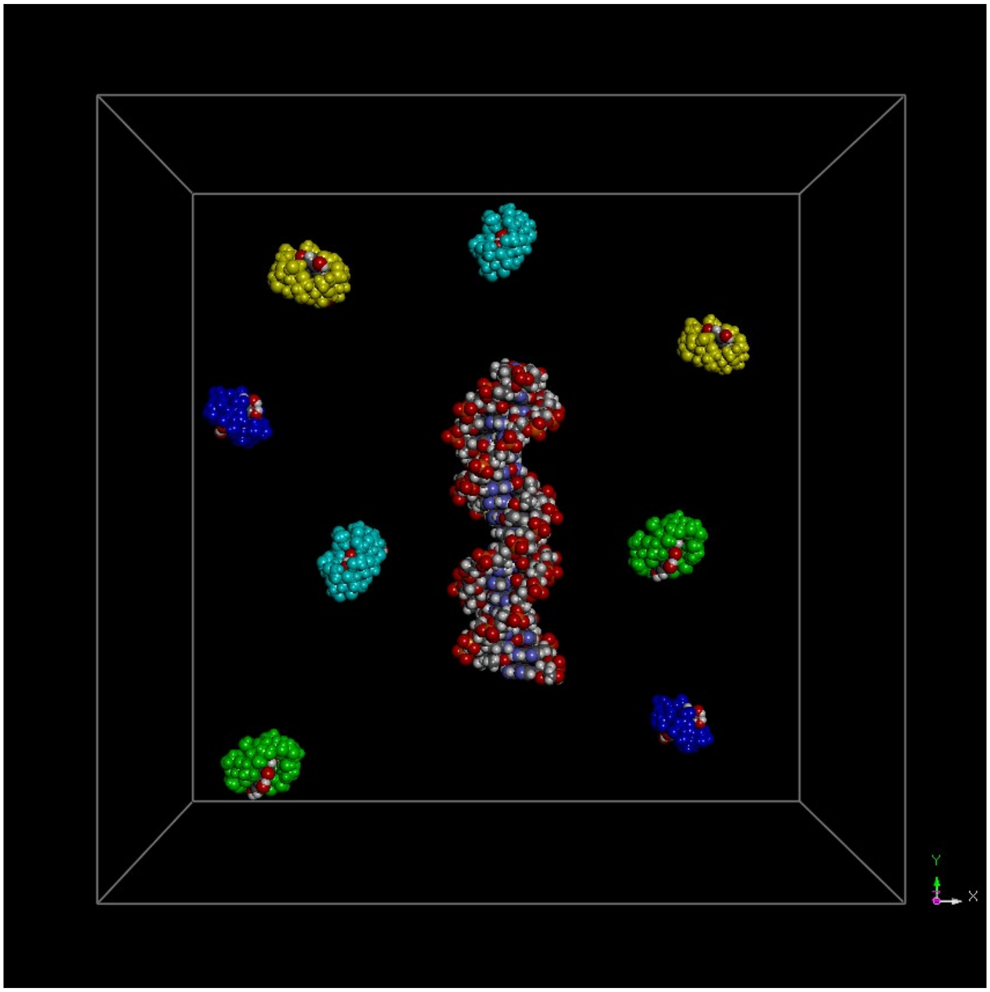

Figure S18. Initial non-optimized geometry with eight different βCD/Q inclusion complexes in a 1:1 stoichiometry as shown in Figure **S4**, in a random arrangement in the simulation cell (see edge shown in white lines) containing the DNA in the center. Color code of DNA and quercetin drug included in host–guest complexes: carbon atoms are in gray; oxygen in red; nitrogen in blue; and hydrogen in white. For clarity, all βCD atoms of the same kind of inclusion complex (Figure S4) are of the same color, yellow or green or light blue or blue. All atoms are in CPK representation.

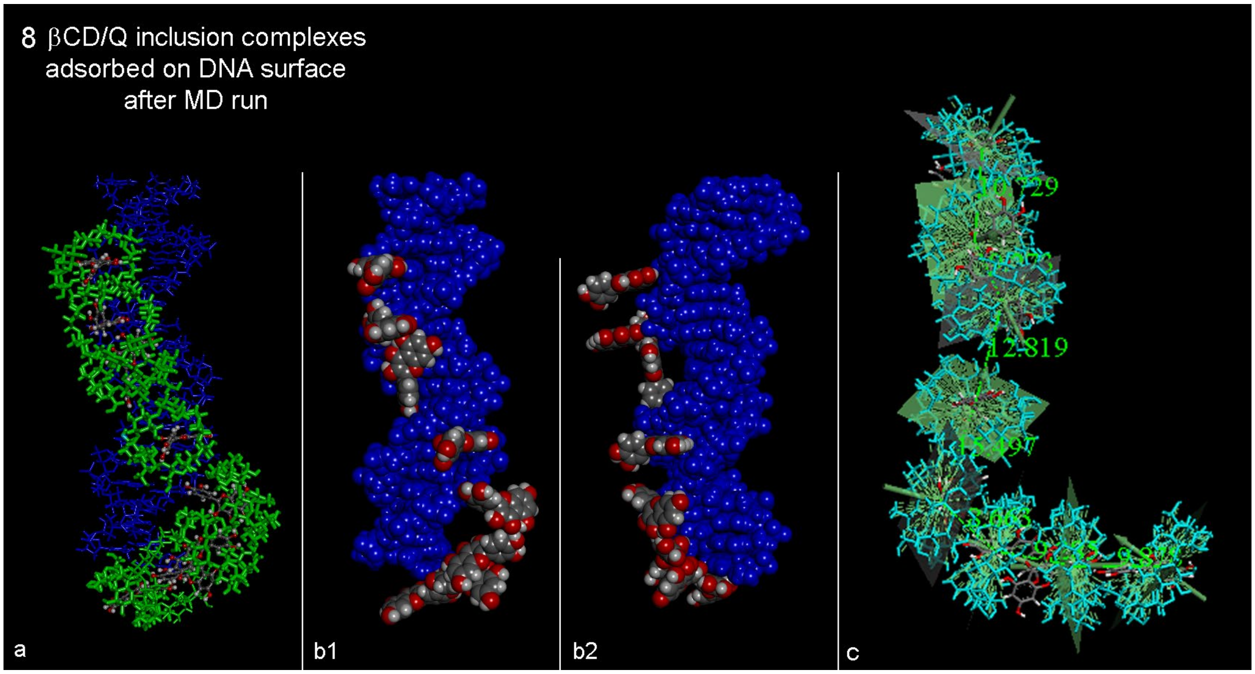

Figure S19. Panel **a** shows the final optimized geometry obtained after an MD run lasting 50 ns starting from the initial geometry shown in Figure S18, without the edges of simulation box for clarity.

Panels **b1** and **b2** show details of the only DNA and 8 quercetin molecules without the βCD atoms as in the final adsorption geometry. Color code for Q molecules: carbon atoms are in gray; oxygen in red; and hydrogen in white. For clarity, all DNA atoms are in blue and, in Panel **a**, all βCD atoms in stick representation are in green.

Panel **c** shows all eight quercetin molecules colored by atoms and, for all eight βCDs colored in light blue for clarity, the best-fit planes with the arrow pointing towards the secondary rim of each βCD. For all eight βCDs, the centers of mass are shown together with their distances in a particular alignment which follows the double-stranded DNA architecture. The distances calculated are equal to 9.878 Å, 9.285 Å, 8.663 Å, 15.497 Å, 12.819 Å, 9.273 Å, and 10.729 Å.

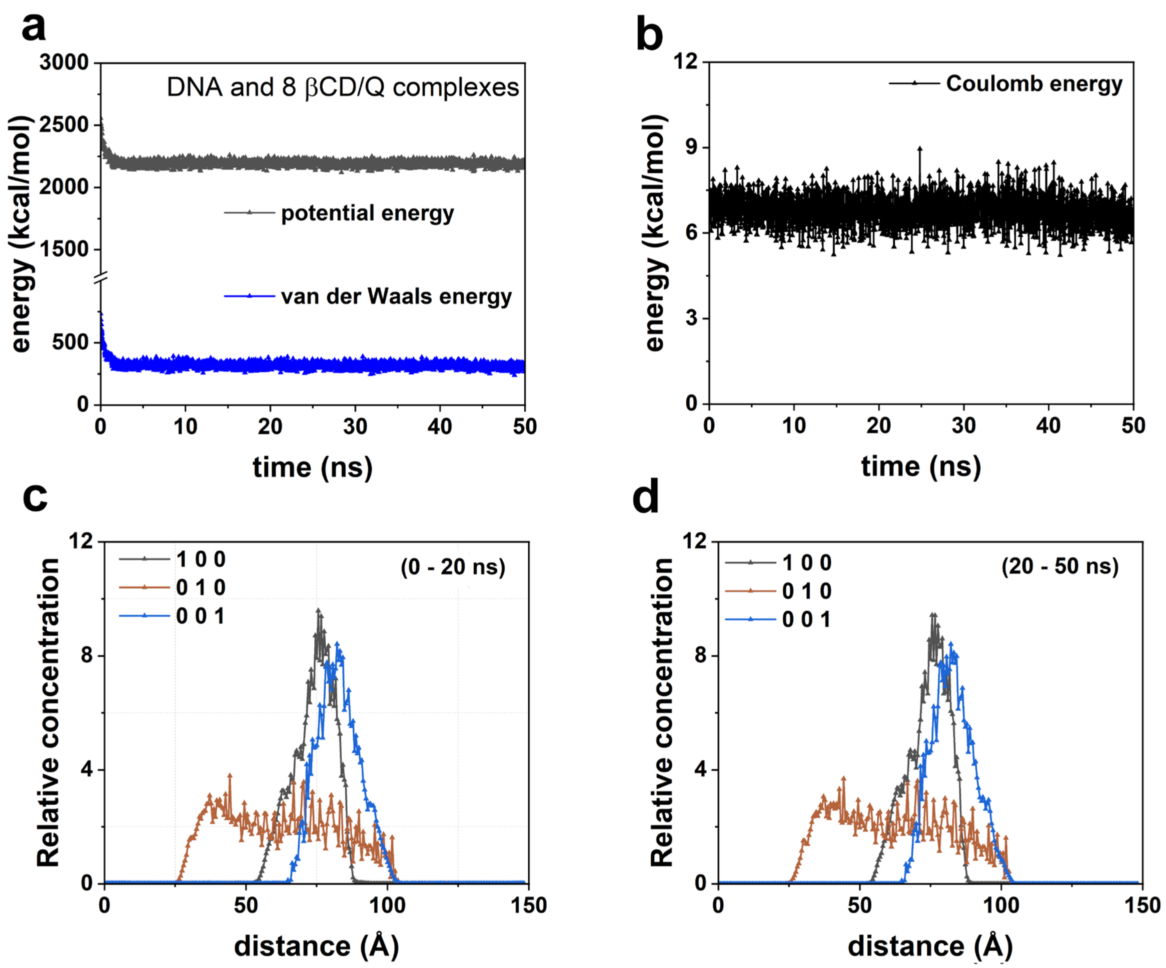

Figure S20. Panels **a** and **b** show the potential energy, van der Waals contribution and Coulomb energy calculated during an MD run lasting 50 ns starting from the optimized geometry shown in Panel **a** of **Figure S18**.

Panels **c** and **d** display the relative concentration of all atoms in the simulation box calculated during the MD run for all frames periodically saved every 20 ps from o to 20 ns in Panel **c**, and from 20 to 50 ns in Panel **d**, respectively.

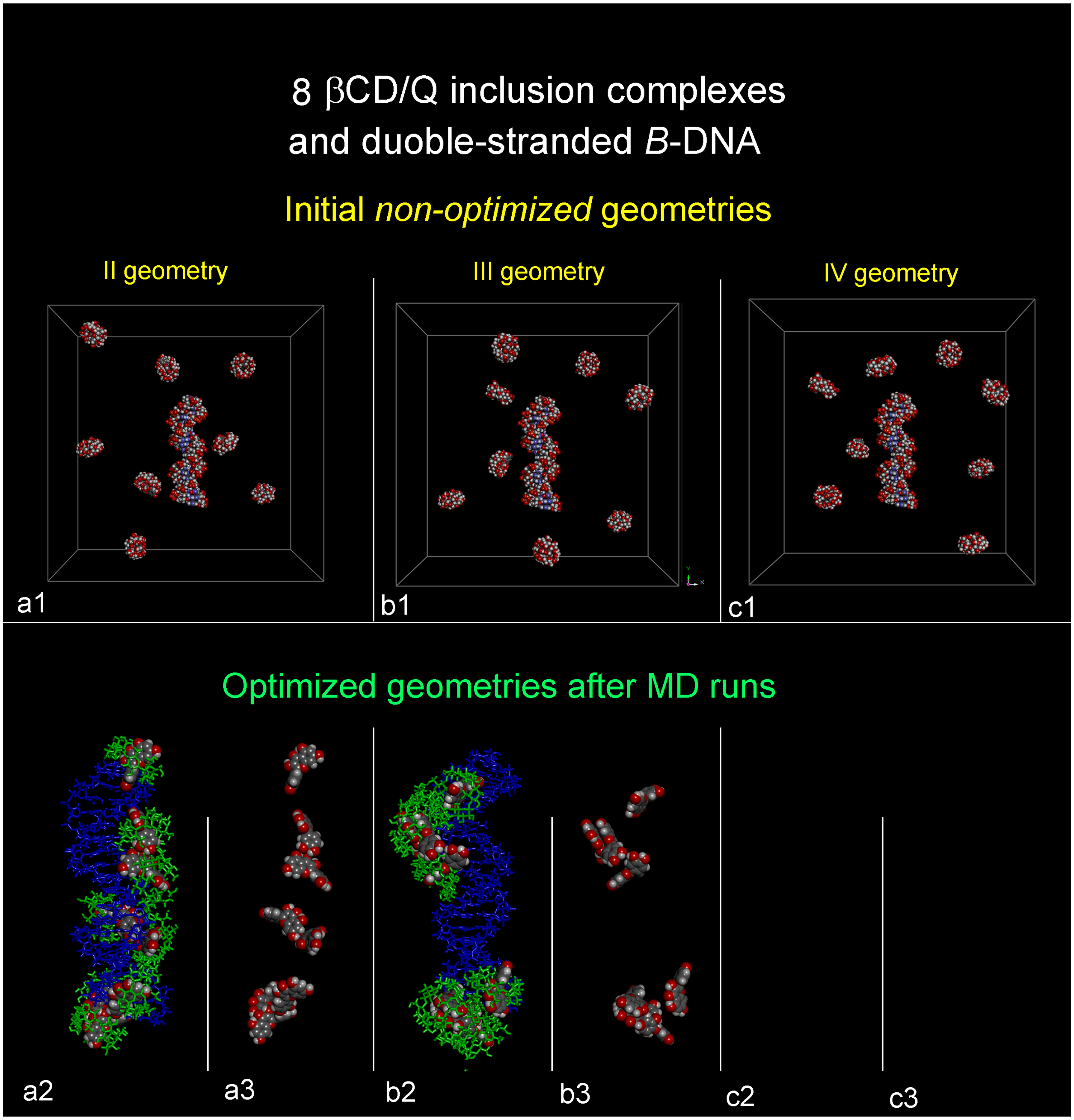

Figure S21. Panels **a1**, **b1**, and **c1** illustrate the three different initial *non-optimized* geometries with eight βCD/Q inclusion complexes in a 1:1 stoichiometry as shown in Figure **S4**, in a random arrangement in the simulation cell (see edge shown in white lines) containing the *B*-DNA in the central part. Color code: carbon atoms are in gray; oxygen in red; nitrogen in blue; and hydrogen in white. All atoms are in CPK representation. Panels **a2**, **b2**, and **c2** show the three final optimized geometries obtained after MD runs lasting 50 ns and the energy minimizations. All DNA atoms are in blue, the β-cyclodextrins are in green. Color code for quercetin drug molecules: carbon atoms are in gray; oxygen in red; nitrogen in blue; and hydrogen in white. Only quercetin molecules are in CPK representation. Panels **a3**, **b3**, and **c3** report the details of the arrangement of drug molecules only.

Intermolecular Interactions Between *B*-DNA and βCD/Q Host–guest Complexes in a 12:12 Stoichiometry

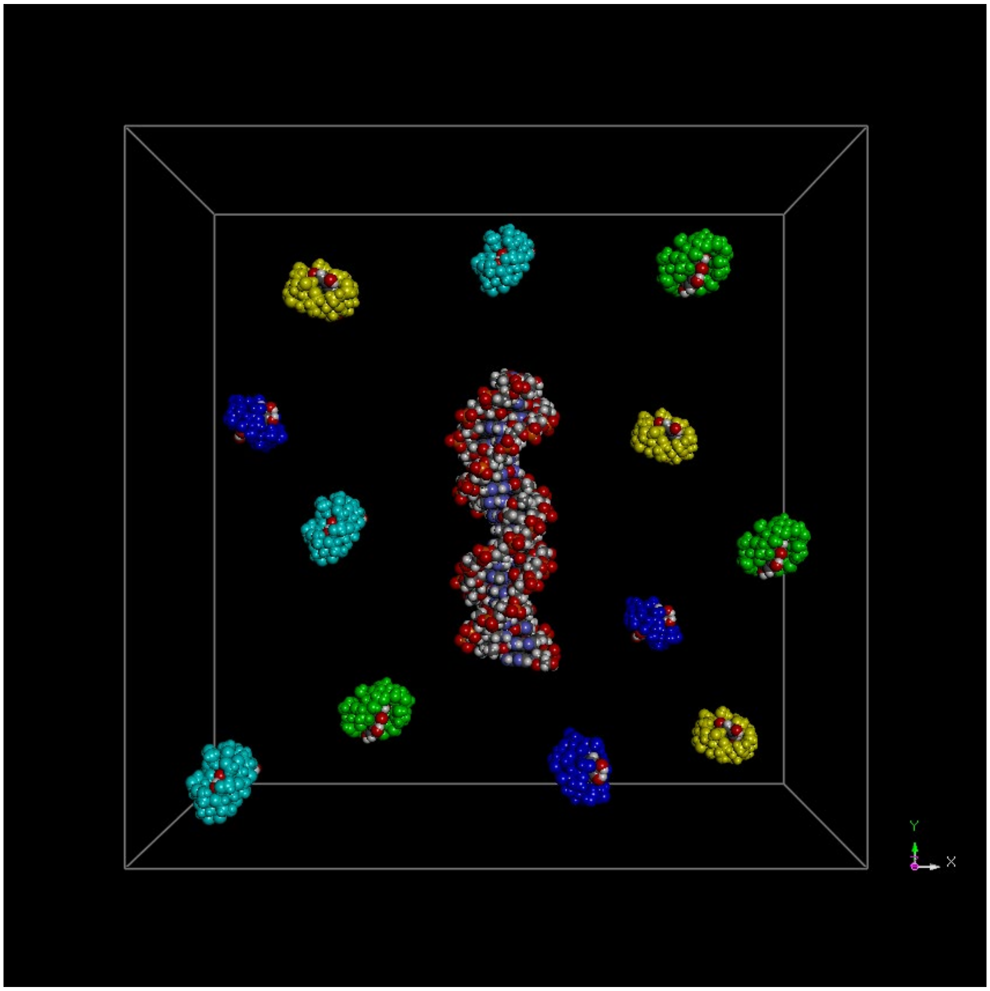

Figure S22. Initial non-optimized geometry with twelve different βCD/Q inclusion complexes in a 1:1 stoichiometry as shown in Figure **S4**, in a random arrangement in the simulation cell (see edge shown in white lines) containing the DNA in the center. Color code of DNA and quercetin drug included in host–guest complexes: carbon atoms are in gray; oxygen in red; nitrogen in blue; and hydrogen in white. For clarity, all βCD atoms of the same kind of inclusion complex (Figure **S4**) are of the same color, yellow or green or light blue or blue. All atoms are in CPK representation.

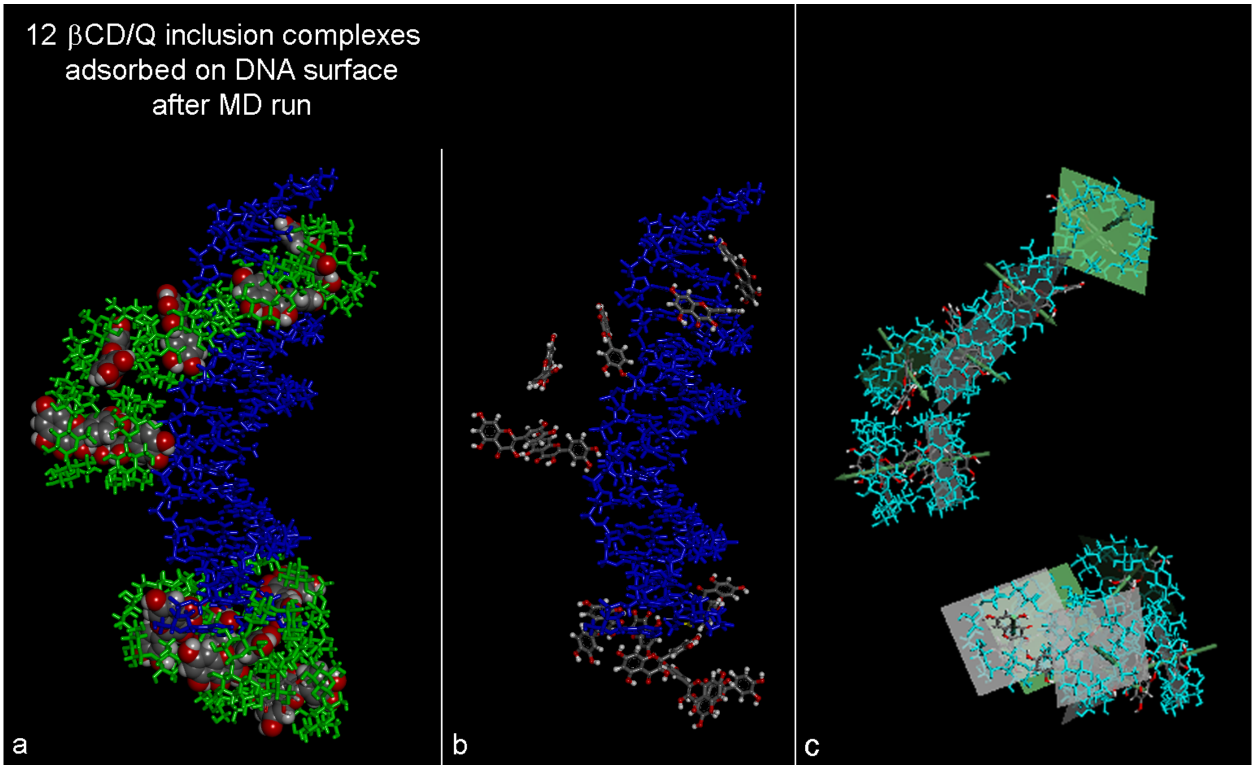

Figure S23. Panel **a** shows the final optimized geometry obtained after an MD run lasting 50 ns starting from the initial geometry shown in Figure S21, without the edges of the simulation box for clarity.
Panel **b** shows a detail of the only DNA and 12 quercetin molecules as in the final adsorption geometry without the βCD atoms. Color code for Q molecules in CPK representation: carbon atoms are in gray; oxygen in red; and hydrogen in white. For clarity, all βCD atoms in stick representation are in green and all DNA atoms in blue.
Panel **c** shows all twelve quercetin molecules colored by atoms and, for all twelve βCDs colored in light blue for clarity, the best-fit planes with the arrow pointing towards the secondary rim of each βCD. Note the alignment along the DNA backbone with the formation of some βCD/Q dimers in 2:2 stoichiometry, as well as an aggregation of inclusion complexes at the lower end of the DNA.

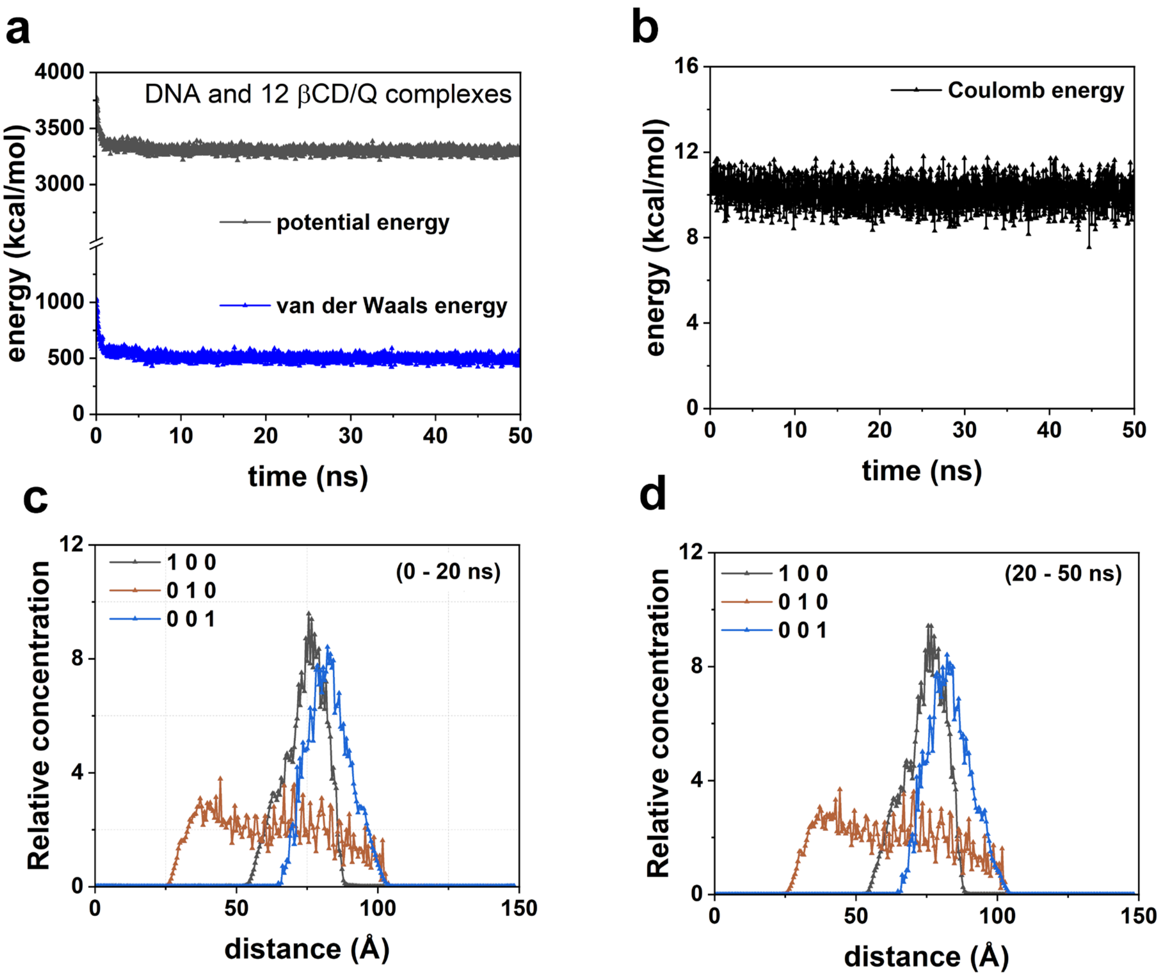

Figure S24. Panels **a** and **b** show the potential energy, van der Waals contribution and Coulomb energy calculated during an MD run lasting 50 ns starting from the optimized geometry shown in Panel **a** of **Figure S22**.

Panels **c** and **d** display the relative concentration of all atoms in the simulation box calculated during the MD run for all frames periodically saved every 20 ps from 0 to 20 ns in Panel **c**, and from 20 to 50 ns in Panel **d**, respectively.

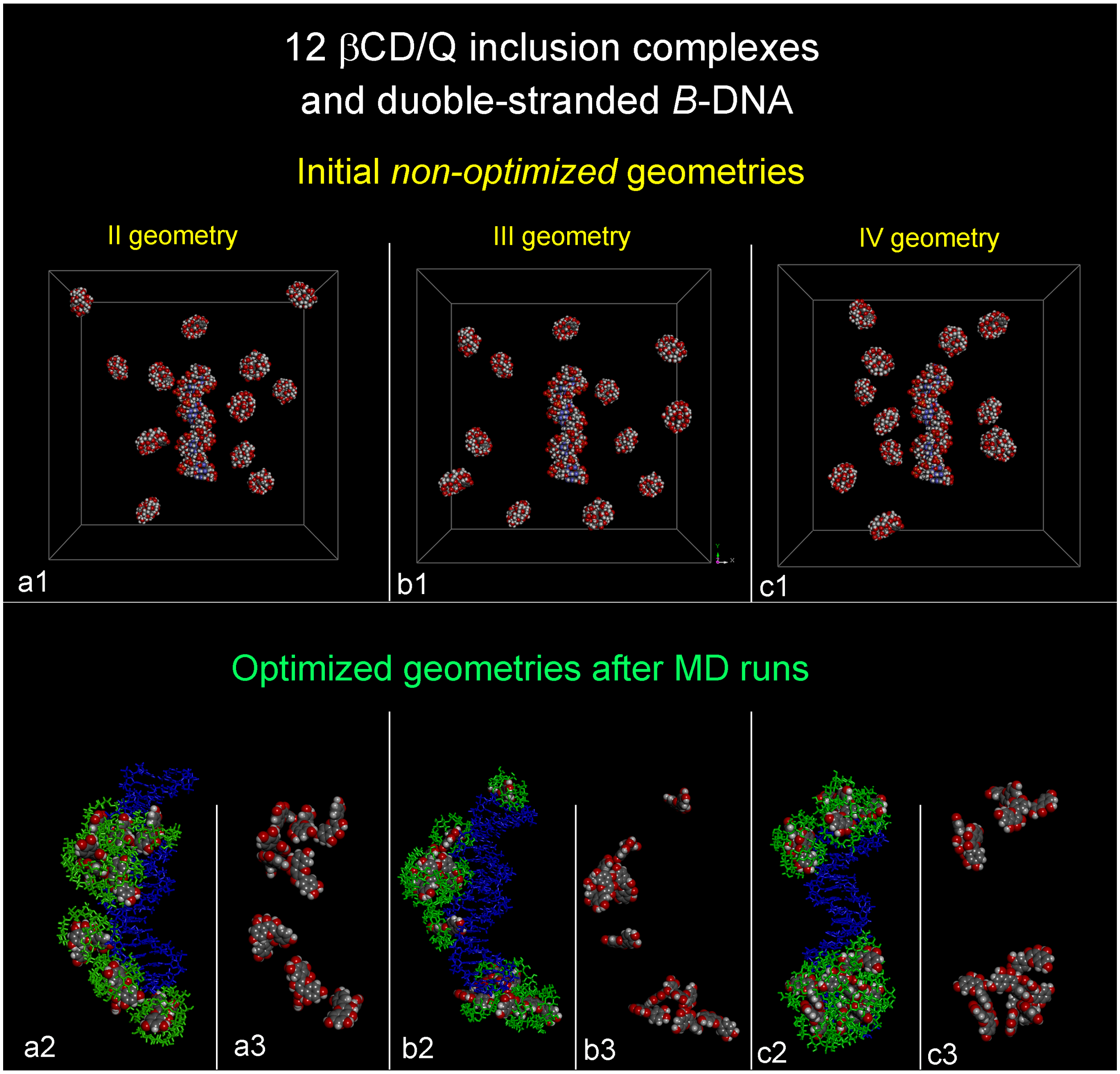

Figure S25. Panels **a1**, **b1**, and **c1** illustrate the three different initial *non-optimized* geometries with twelve βCD/Q inclusion complexes in a 1:1 stoichiometry as shown in Figure **S4**, in a random arrangement in the simulation cell (see edge shown in white lines) containing the *B*-DNA in the central part. Color code: carbon atoms are in gray; oxygen in red; nitrogen in blue; and hydrogen in white. All atoms are in CPK representation. Panels **a2**, **b2**, and **c2** show the three final optimized geometries obtained after MD runs lasting 50 ns and the energy minimizations. All DNA atoms are in blue, the β-cyclodextrins are in green. Color code for quercetin drug molecules: carbon atoms are in gray; oxygen in red; nitrogen in blue; and hydrogen in white. Only quercetin molecules are in CPK representation. Panels **a3**, **b3**, and **c3** report the details of the arrangement of drug molecules only……………………….……….……….………………...S30

Intermolecular Interactions Between B-DNA and βCD/Q Host–guest Complexes in a 16:16 Stoichiometry

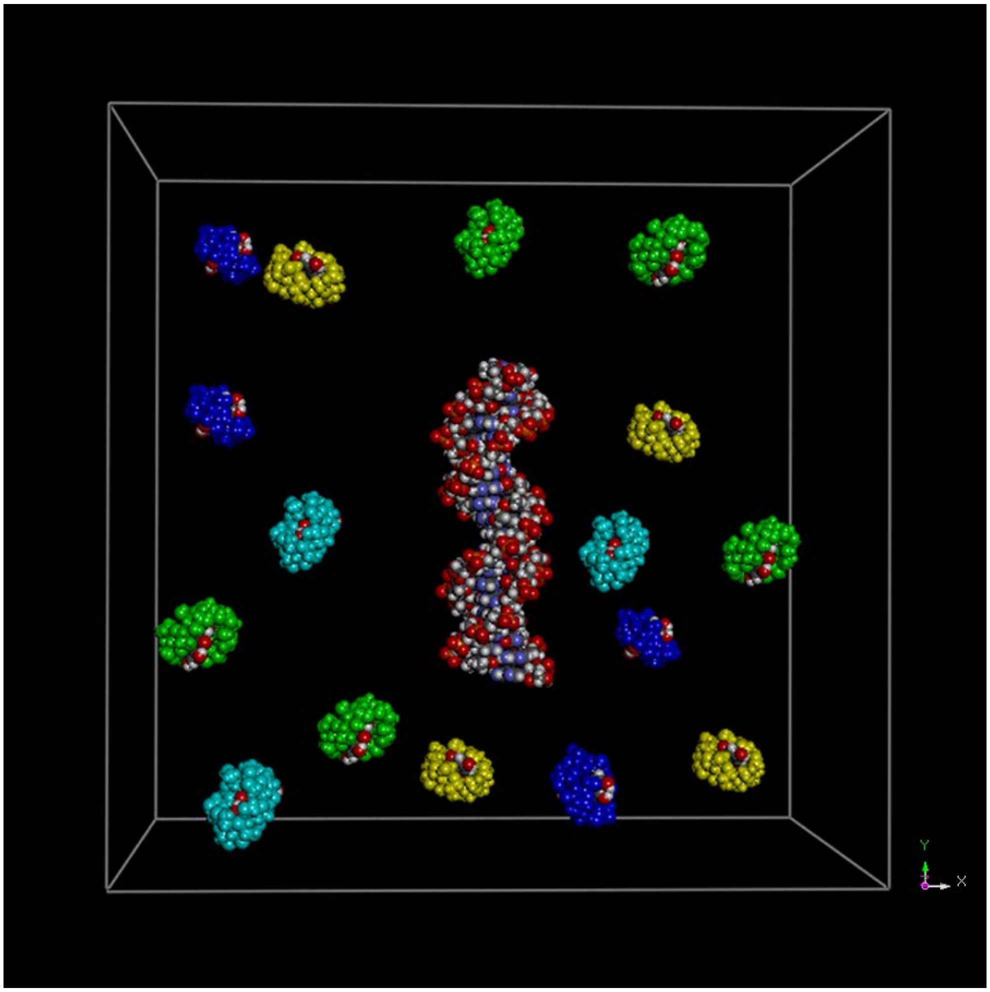

Figure S26. Initial non-optimized geometry with sixteen different βCD/Q inclusion complexes in a 1:1 stoichiometry as shown in Figure **S4**, in a random arrangement in the simulation cell (see edge shown in white lines) containing the DNA in the center. Color code of DNA and quercetin drug included in host–guest complexes: carbon atoms are in gray; oxygen in red; nitrogen in blue; and hydrogen in white. For clarity, all βCD atoms of the same kind of inclusion complex (Figure **S4**) are of the same color, yellow or green or light blue or blue. All atoms are in CPK representation.

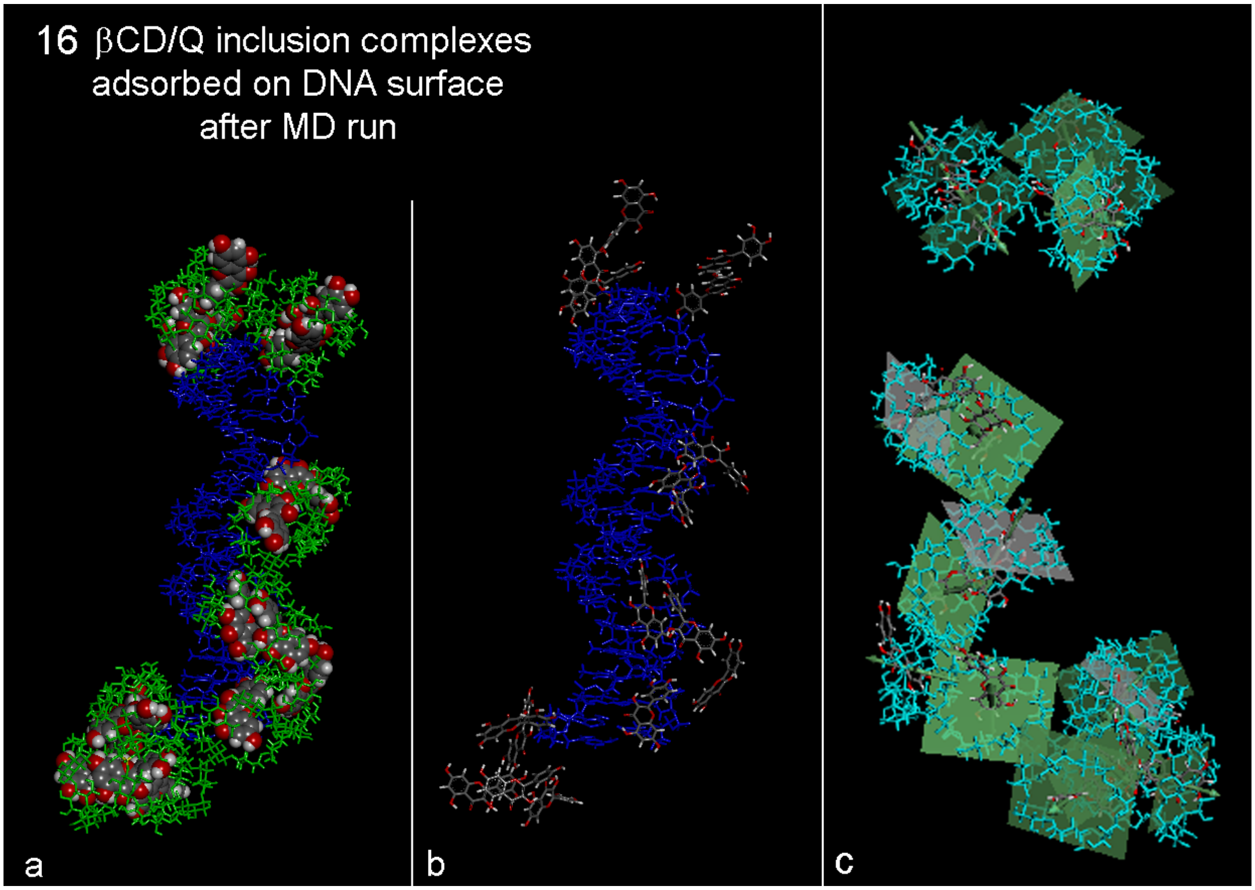

Figure S27. Panel **a** shows the final optimized geometry obtained after an MD run lasting 50 ns starting from the initial geometry shown in Figure S24, without the edges of simulation box for clarity.
Panel **b** shows a detail of the only DNA and 16 quercetin molecules as in the final adsorption geometry without the βCD atoms. Color code for Q molecules in CPK representation: carbon atoms are in gray; oxygen in red; and hydrogen in white. For clarity, all βCD atoms in stick representation are in green, and all DNA atoms in blue.
Panel **c** shows all sixteen quercetin molecules colored by atoms and, for all sixteen βCDs colored in light blue for clarity, the best-fit planes with the arrow pointing towards the secondary rim of each βCD. Note the alignment along the DNA backbone with the formation of some βCD/Q dimers in 2:2 stoichiometry and also an aggregation of inclusion complexes at the ends of the DNA.

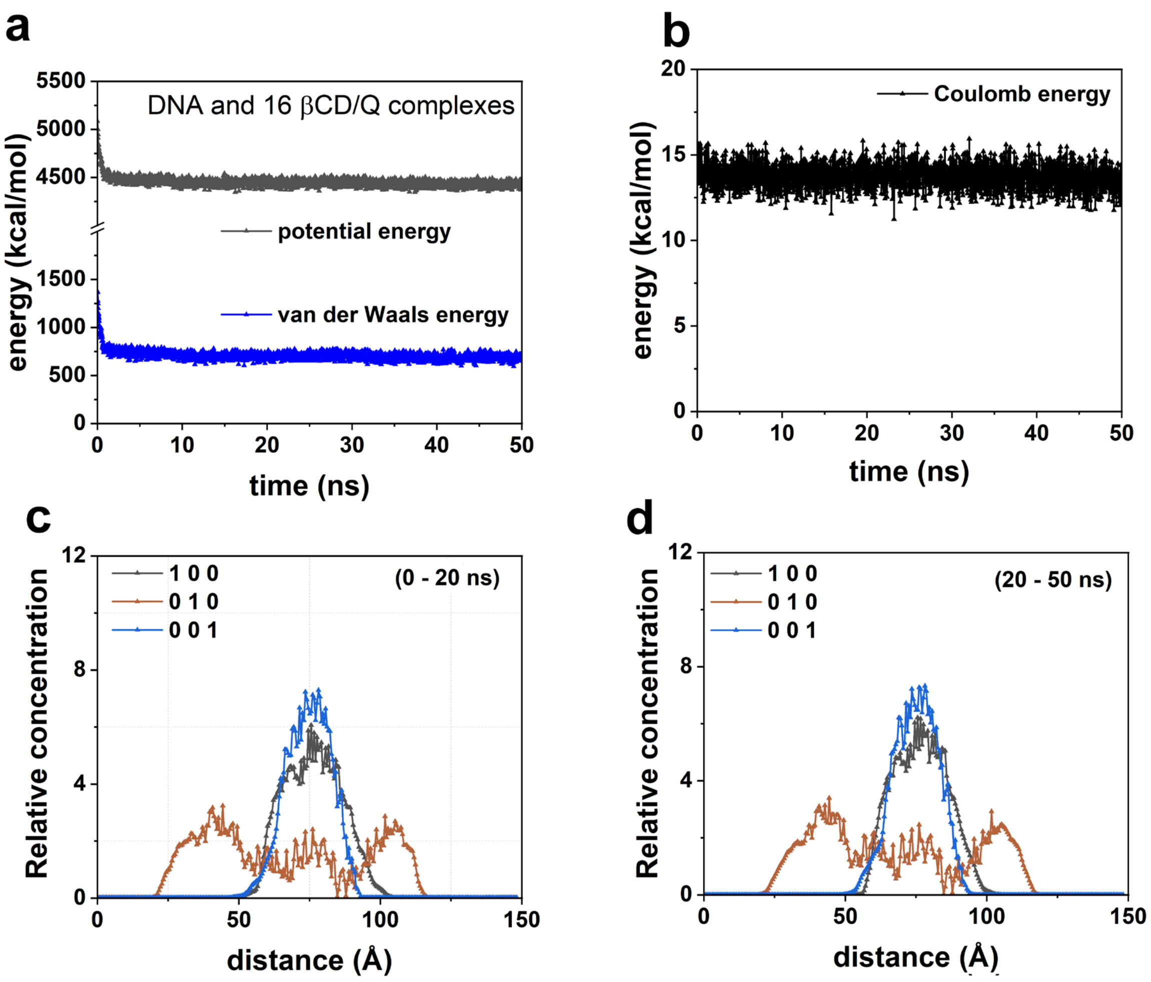

Figure S28. Panels **a** and **b** show the potential energy, van der Waals contribution and Coulomb energy calculated during an MD run lasting 50 ns starting from the optimized geometry shown in Panel **a** of **Figure S26**.

Panels **c** and **d** display the relative concentration of all atoms in the simulation box calculated during the MD run for all frames periodically saved every 20 ps from 0 to 20 ns in Panel **c**, and from 20 to 50 ns in Panel **d**, respectively.

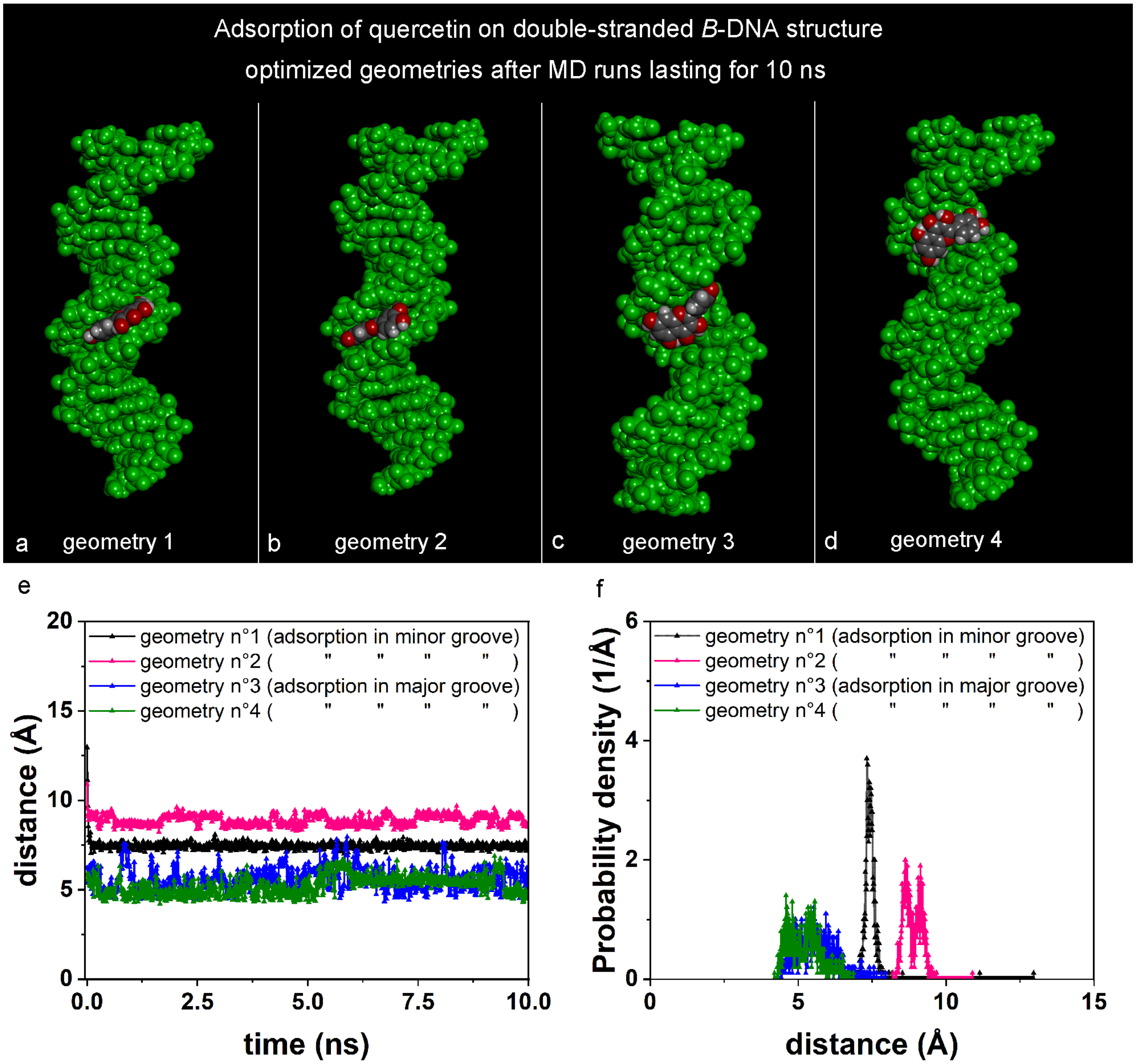

Figure S29. Side view of the optimized geometries obtained after MD runs lasting 10 ns related to the adsorption of quercetin on the DNA surface starting from four different geometries with drug molecules parallel or perpendicular near to a minor or major groove. The animation of the four MD runs is shown in file.avi shown below. The DNA is colored in green and quercetin molecules by atoms (carbon atoms in gray, oxygen atoms in red and hydrogen atoms in white) are in CPK representation. Panels **e** and **f** show the distance of the quercetin c.o.m. and the line that define the axis along which the DNA fragment unfolds calculated during the four MD runs performed.

dyn_DNA_1Q_minor G_1 geometry_10ns.avi
dyn_DNA_1Q_minor G_2 geometry_10ns.avi
dyn_DNA_1Q_major G_3 geometry_10ns.avi
dyn_DNA_1Q_major G_4 geometry_10ns.avi

Intermolecular Interactions Between *B*-DNA and Quercetin Molecules at Different Concentrations

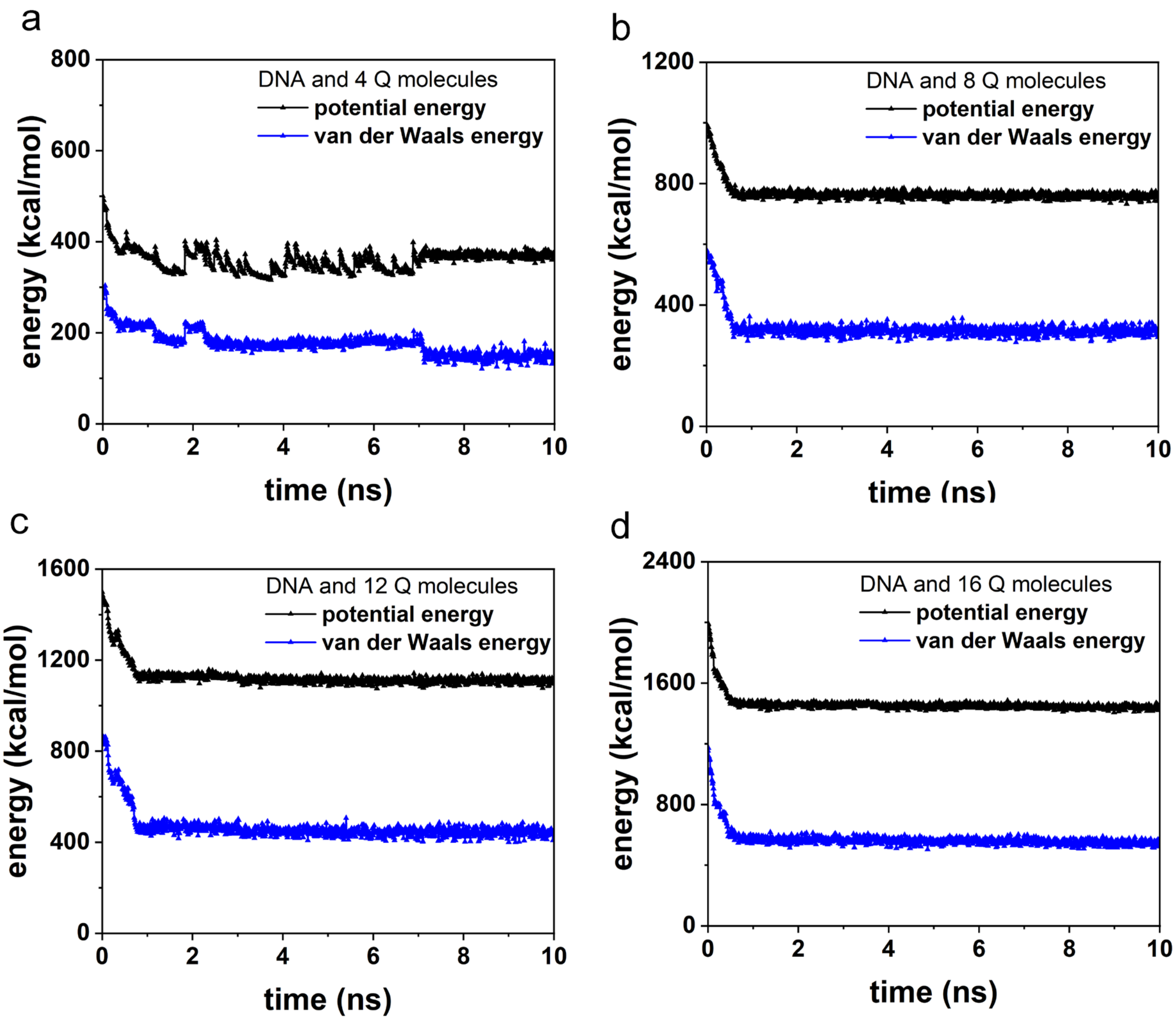

Figure S30. Potential energy and van der Waals contribution calculated during MD runs lasting 10 ns starting from the optimized geometry considering DNA in the central part of the simulation cell, and four, eight, twelve, and sixteen quercetin molecules in Panels **a**, **b**, **c**, and **d**, respectively.

Intermolecular Interactions Between *B*-DNA and one βCD molecule

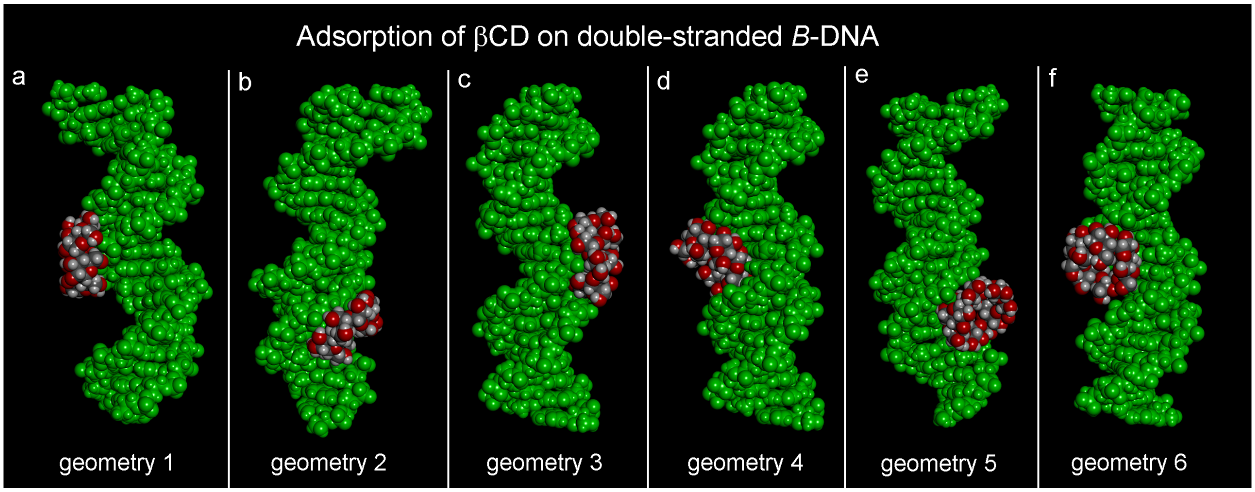

Figure S31. Optimized geometries related to the interaction between double-stranded *B*-DNA and one in βCD obtained after six different MD runs lasting 10 ns starting from six different initial non-optimized geometries with the βCD primary rim parallel to a minor or major DNA groove, the βCD secondary rim parallel to a minor or major DNA groove, and the βCD best-fit plane perpendicular to the DNA axis near to a minor or major groove.

dyn_DNA1cd_P_m_10ns.avi
dyn_DNA1cd_P_MJ_10ns.avi
dyn_DNA1cd_S_m_10ns.avi
dyn_DNA1cd_S_MJ_10ns.avi
dyn_DNA1cd_perp_m_10ns.avi
dyn_DNA1cd_perpMJ_10ns.avi

Table S4. Interaction energy (kJ/mol), intramolecular H-bonds in βCD and intermolecular H-bonds B-DNA/βCD in the **final optimized geometries** after a 10 ns MD run, as shown in Figure **S31**.

Optimized geometry	E_{int} (kJ/mol)	βCD intramolecular H-bonds	DNA/ βCD intramolecular H-bonds
Geometry 1<br>P rim near DNA	-177.5	3<br>(2 H-bonds between -**OH** βCD **primary** rim···**O** DNA phosphate group)	11
Geometry 2<br>S rim near DNA	-149.3	3<br>(3 H-bonds between -**OH** βCD **secondary** rim···**DNA bases**)	11
Geometry 3<br>S rim near DNA	-178.2	2 H-bonds<br>(2 H-bonds between -**OH** βCD **secondary** rim···**O** DNA phosphate group)	15
Geometry 4<br>S rim near DNA	-174.3	5 H-bonds<br>(4 H-bonds between -**OH** βCD **secondary** rim···**DNA bases**)	12
Geometry 5<br>P rim near DNA	-162.3	0<br>(primary rim near major groove of DNA)	12
Geometry 6<br>S rim near DNA	-184.8	4<br>(1 H-bonds between -**OH** βCD **secondary** rim···**O** DNA phosphate group)<br>(3 H-bonds between -**OH** βCD **secondary** rim···**DNA bases**)	16

Intermolecular Interactions Between *B*-DNA and βCDs at Different Concentrations

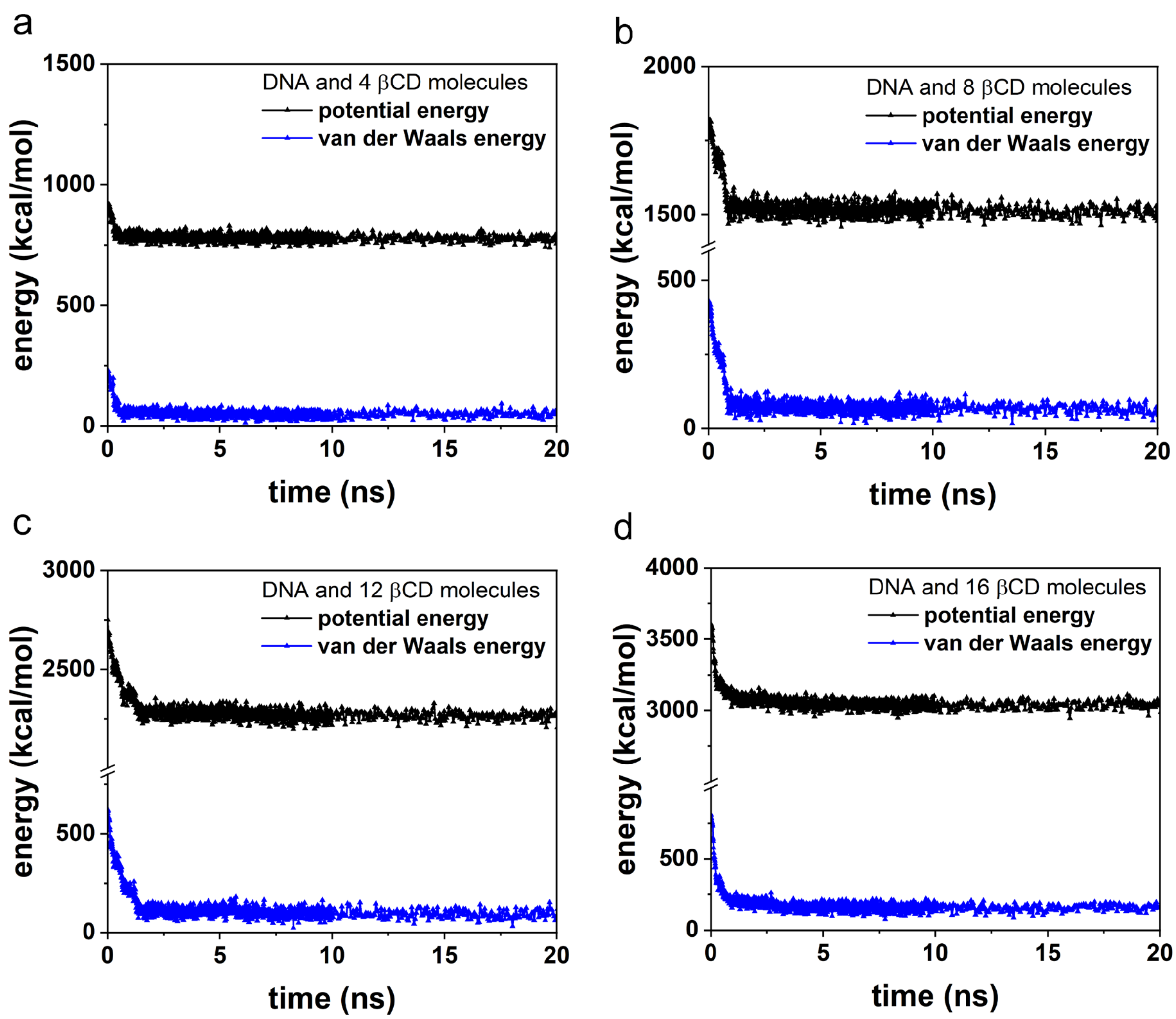

Figure S32. Potential energy and van der Waals contribution calculated during MD runs lasting 20 ns starting from the optimized geometry obtained considering DNA in the central part of the simulation cell, and four, eight, twelve, and sixteen βCDs in Panels **a**, **b**, **c**, and **d**, respectively.

Intermolecular Interactions Between βCDs at Different Concentrations

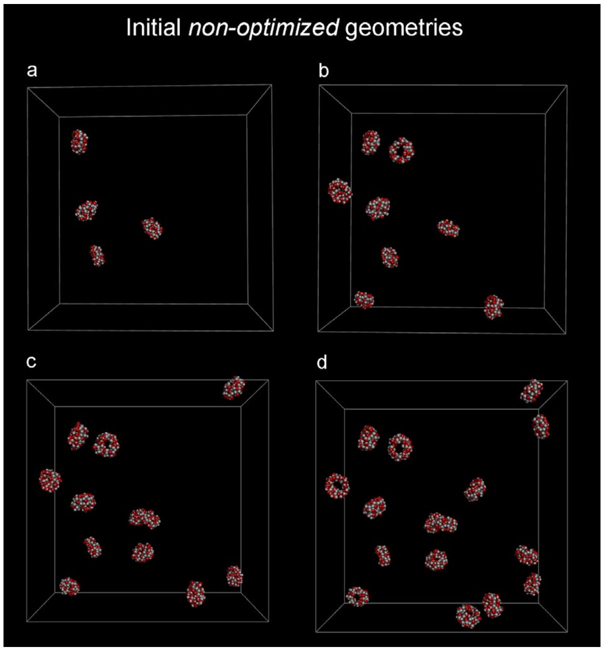

Figure S33. Initial non-optimized geometries related to four, eight, twelve, and sixteen βCDs in the simulation cell. Color code: carbon atoms are in gray; oxygen in red; and hydrogen in white. All atoms are in CPK representation.

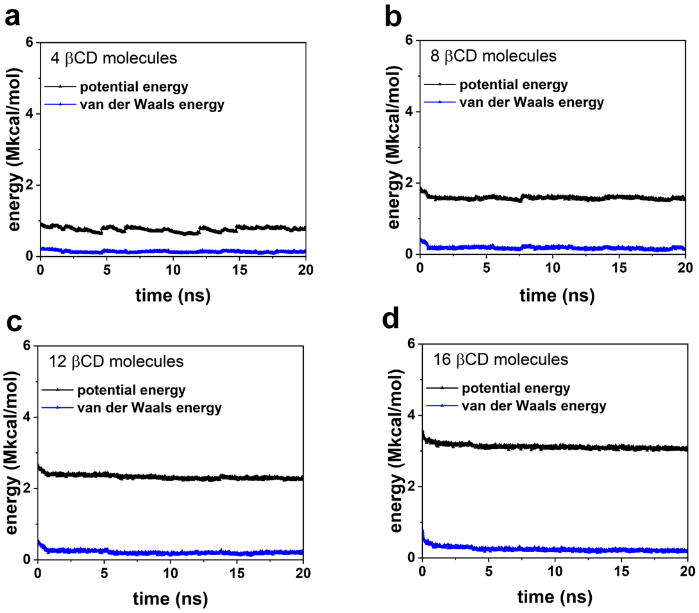

Figure S34. Potential energy and van der Waals contribution calculated during MD runs lasting 20 ns starting from the non-optimized geometries shown in Figure **S33** related to four, eight, twelve, and sixteen βCDs in Panels **a**, **b**, **c**, and **d**, respectively.

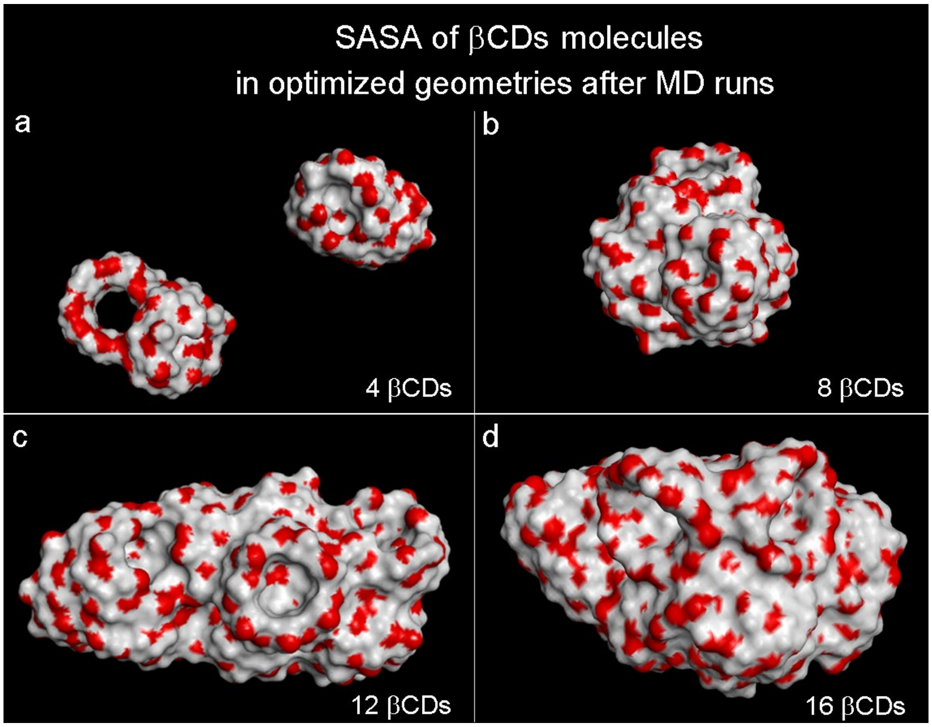

Figure S35. Solvent-accessible surface area (SASA) colored by atoms of the final optimized geometries related to four, eight, twelve, and sixteen βCDs in Panels **a**, **b**, **c**, **d** respectively in the simulation cell after MD runs lasting 20 ns. The initial non-optimized geometries related to four, eight, twelve, and sixteen βCDs in the simulation cell are reported in Figure **S33**. The color code is the same as in Figure **S33**.

dyn_4Bcd_20ns.avi
dyn_8Bcd_20ns.avi
dyn_12Bcd_20ns.avi
dyn_16Bcd_20ns.avi

Intermolecular Interactions Between βCDs and Quercetin Molecules at Different Concentrations

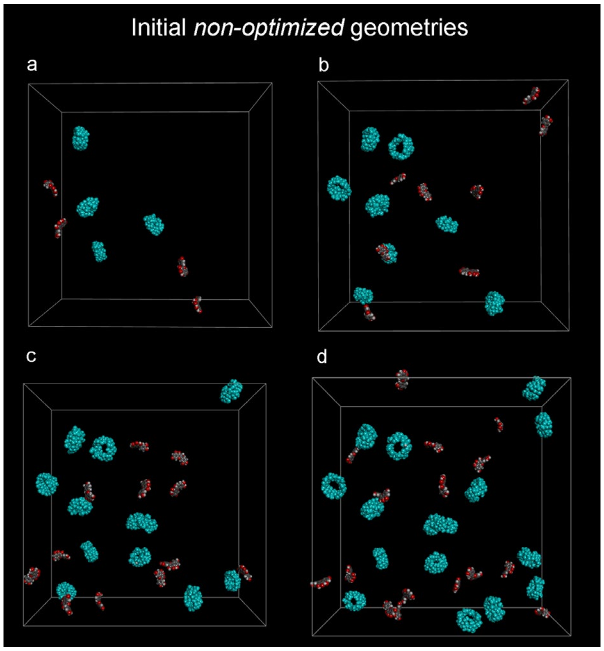

Figure S36. Initial non-optimized geometries related to four, eight, twelve, and sixteen βCDs and quercetin molecules in the simulation cell in Panels **a**, **b**, **c**, **d** respectively. Color code: all βCD atoms are in light blue, quercetin carbon atoms are in gray; oxygen in red; and hydrogen in white. All atoms are in CPK representation.

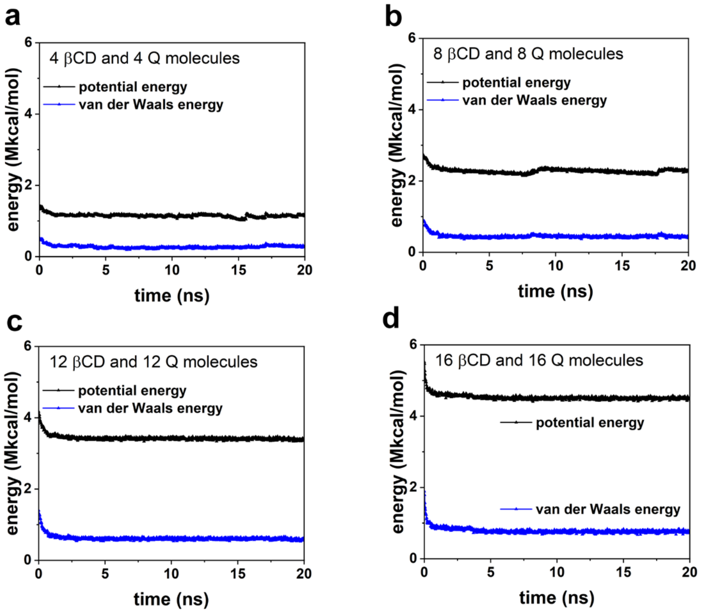

Figure S37. Potential energy and van der Waals contribution calculated during MD runs lasting 20 ns starting from the non-optimized geometries shown in Figure **S33** related to four, eight, twelve, and sixteen βCDs in Panels **a**, **b**, **c**, and **d**, respectively.

dyn_4Q_4Bcd_20ns.avi
dyn_8Q_8Bcd_20ns.avi
dyn_12Q_12Bcd_20ns.avi
dyn_16Q_16Bcd_20ns.avi

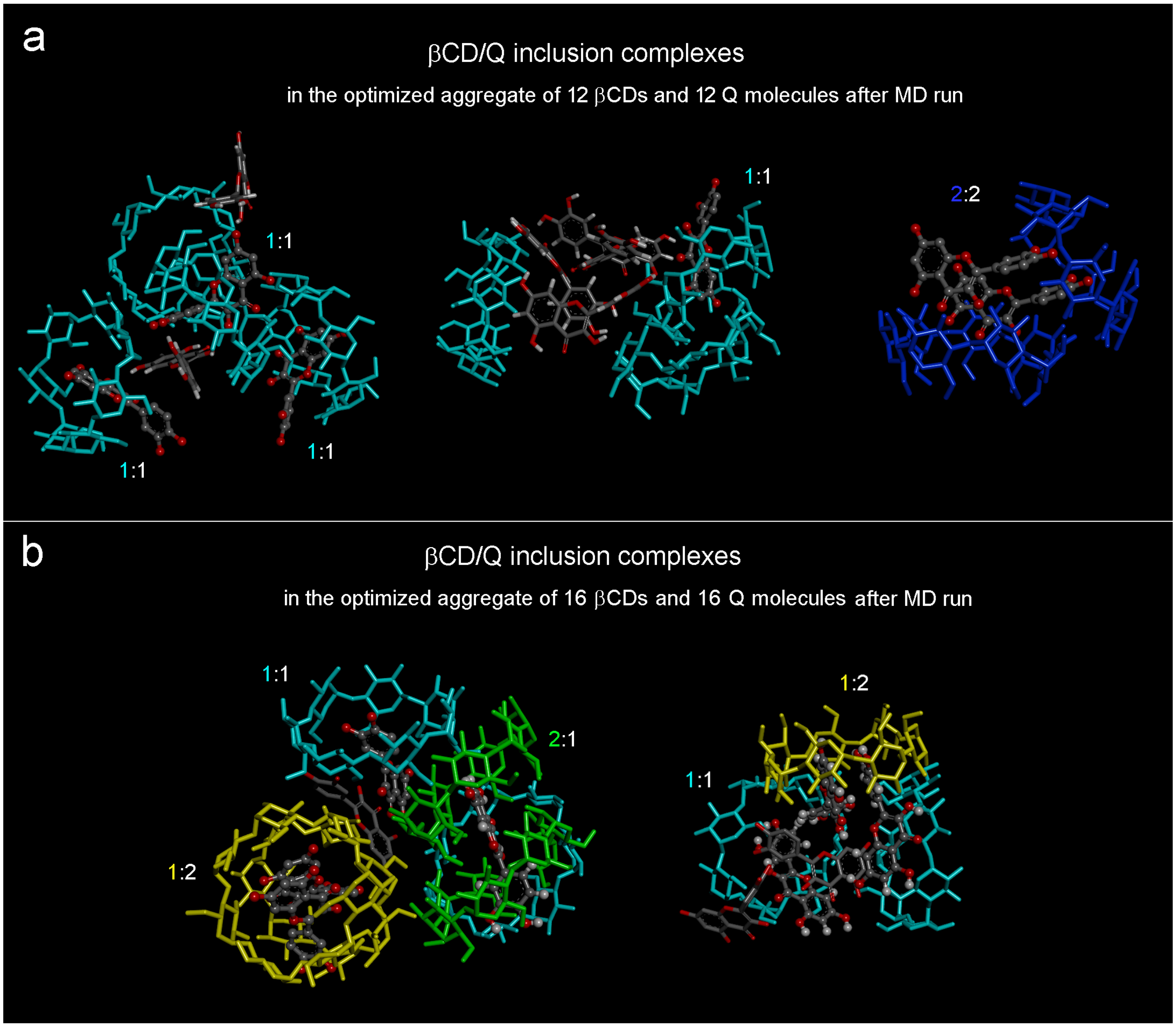

Figure S38. Detail of some inclusion complexes in the final optimized geometries related to twelve (Panel **a**) and sixteen (Panel **b**) βCDs and quercetin molecules in the simulation cell after MD runs lasting 20 ns. The color code for quercetin molecules is the same as in Figure **S36**. The βCDs involved in βCD/Q 1:1 stoichiometry are colored in light blue, βCDs involved in βCD/Q in 1:2 and 2:1 stoichiometry are colored in yellow and in green, respectively, and, finally, the βCDs involved in βCD/Q 2:2 stoichiometry in Panel **a** are colored in blue. The βCDs are in stick representation, encapsulated quercetin molecules in ball and stick, Q molecules not included in stick representation. The hydrogen atoms are omitted for clarity.

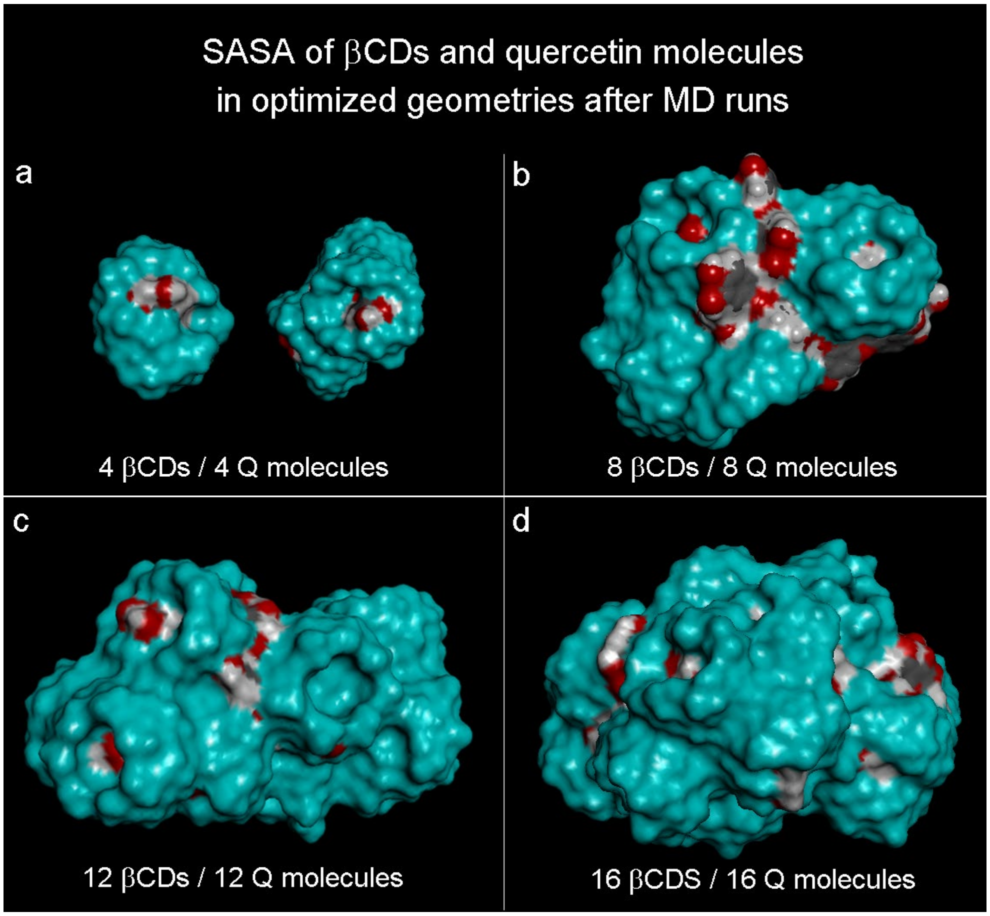

Figure S39. SASA colored by atoms of the final optimized geometries related to four, eight, twelve, and sixteen βCDs and quercetin molecules in the simulation cell after MD runs lasting 20 ns in Panels **a**, **b**, **c**, **d** respectively. The color code is the same as in Figure **S36**.

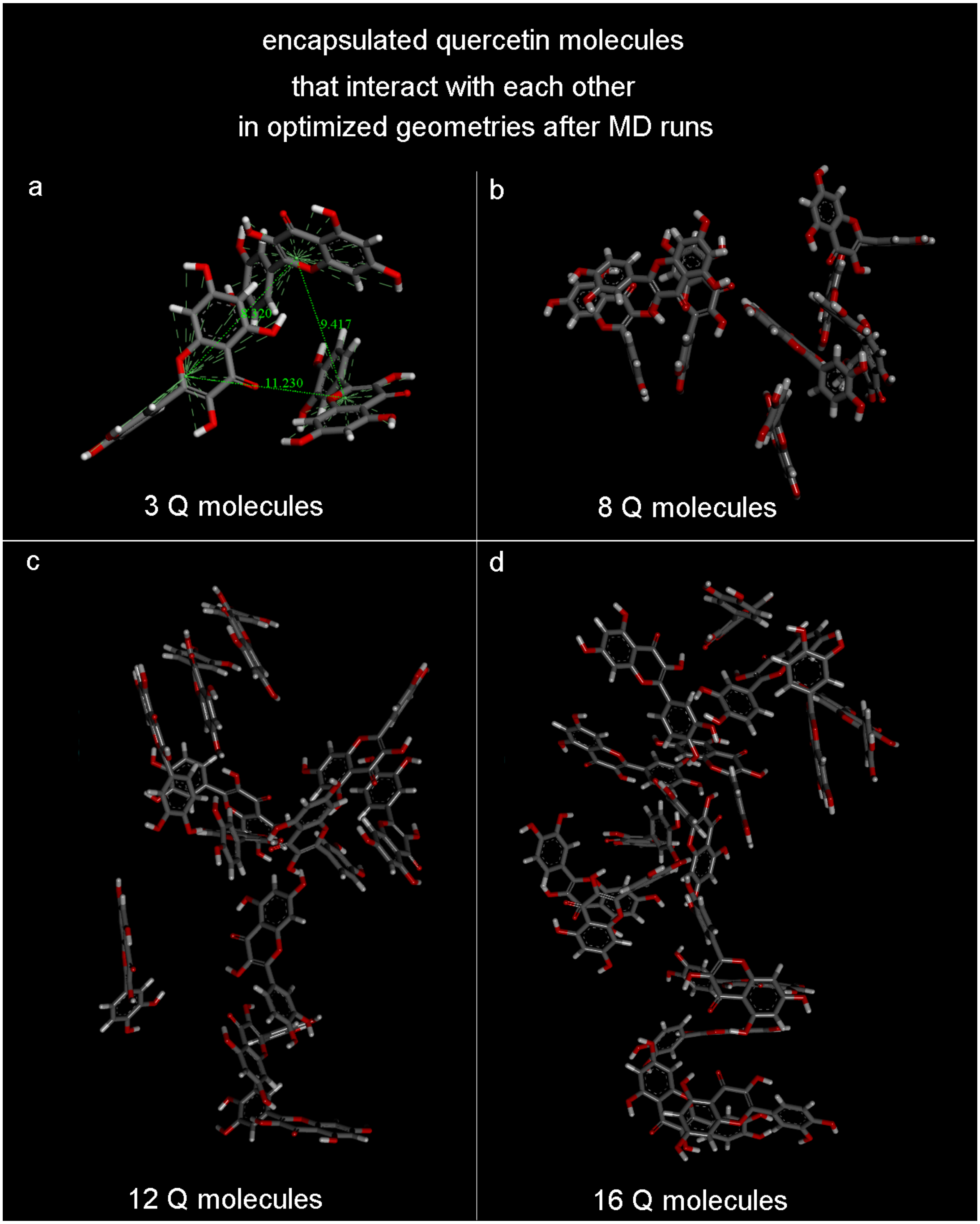

Figure S40. Encapsulated quercetin molecules that interact with each other in the hydrophobic βCD cavities in the final optimized geometries shown in Figure **S39** related to four, eight, twelve, and sixteen βCDs and quercetin molecules in the simulation cell in Panels **a**, **b**, **c**, **d** respectively after MD runs lasting 20 ns, in this figure without the representation of βCDs. The color code is the same as in Figure **2**.

Concentration profile

The Concentration profile is calculated for 3D periodic structures by computing the **profile of atom density** within evenly spaced slices parallel to the *bc*, *ca*, and *ab* planes. In practice, this is equivalent to taking the *a*, *b*, and *c* components of the fractional coordinates of each atom and independently generating a plot for each component. For 2D periodic structures, the *u* and *v* components of the fractional coordinates are used, resulting in two plots.

As an alternative to generating profiles along each axis, it is also possible to specify a particular direction in the form of an (*h k l*) vector. In this case, Forcite will produce a single plot of the concentration profile parallel to the plane defined by that vector.

Each direction is divided equally into a number of bins (potentially, a different number for each axis). For each atom in the unit cell, a contribution is added into the relevant cell in the binning vector corresponding to each axis. The contribution is a constant value determined so that the average value along each axis becomes 1.0 (i.e., a homogeneous structure would have values close to 1.0).

The relative concentration of a set of atoms in a slab is:

relative [set]slab = [set]slab/[set]bulk

where

[set]slab = (no. atoms in slab)/(volume of slab)
[set]bulk = (total no. atoms in system)/(volume of system)

The relative concentration is a dimensionless quantity, a value of 2 means that there are twice the number of atoms in the slab that if all atoms were distributed homogeneously across the system. The total number of atoms across all slabs is equal to the number of atoms in the entire system. So, the sum of the relative concentrations of all slabs is equal to the number of slabs.

For plotting, the bin values are converted from fractional positions to Cartesian values. To allow for the possibility of the unit cell changing, the values used for the unit cell dimensions are the average over all the frames under consideration.

As with other structural analyses, the concentration profile can be calculated for a subset of the atoms in the input structure by specifying a set in the input document or simply by manually selecting the atoms you wish to include in the calculation.
